# MEK1 as a Synthetic Lethal Target with Cabozantinib in Renal Cell Carcinoma: Insights from CRISPR/Cas9 Screening

**DOI:** 10.3390/genes17070789

**Published:** 2026-07-12

**Authors:** Hirofumi Yoshino, Ikumi Fukuda, Hideki Enokida, Naohiko Seki, Yusuke Goto

**Affiliations:** 1 Department of Urology, Graduate School of Medical and Dental Sciences, Kagoshima University, Kagoshima 890-8520, Japan; hyoshino@m3.kufm.kagoshima-u.ac.jp (H.Y.); urolabo2@m.kufm.kagoshima-u.ac.jp (I.F.); enokida@m.kufm.kagoshima-u.ac.jp (H.E.); 2 Department of Functional Genomics, Chiba University Graduate School of Medicine, Chiba 260-8670, Japan; naoseki@faculty.chiba-u.jp; 3 Department of Urology, Teikyo University Chiba Medical Centre, Chiba 299-0111, Japan

**Keywords:** cabozantinib, CRISPR/Cas9 screening, MEK1, synthetic lethality, RCC

## Abstract

**Background/Objectives**: Cabozantinib is a tyrosine kinase inhibitor that primarily targets MET. It has become an important drug in the treatment of renal cell carcinoma (RCC); however, many patients do not respond to cabozantinib treatment and there is no effective next-line therapy. In this study, we identified molecular-targeted drugs that exhibit synergistic effects with cabozantinib using CRISPR/Cas9 screening. **Methods**: A kinome-wide synthetic lethal CRISPR/Cas9 screen was used to identify target molecules using 786-o RCC cells. A library was generated, and treatment with vehicle or cabozantinib was carried out, followed by next-generation sequencing to identify candidate genes. A combination index based on the Chou–Talalay method was used to evaluate the synergistic effect of cabozantinib through cell viability assays. Xenograft assays were conducted to determine the effect in vivo. **Results**: CRISPR/Cas9-based screening revealed four genes (*MEK1*, *DCLK1*, *DYRK3*, and *FGFR1*) that were candidates for synthetic lethality by cabozantinib in RCC cells. We focused on MEK1 because the MEK1 inhibitor cobimetinib has been approved for melanoma treatment. In a cell proliferation assay using 786-o and A498 RCC cells, the combination of cobimetinib and cabozantinib exhibited a synergistic effect. A xenograft assay also revealed a significant synergistic effect of cobimetinib and cabozantinib. **Conclusions**: CRISPR/Cas9 screening identified MEK1 as a candidate for a synthetic lethal target with cabozantinib in RCC. The combined inhibition of MET/VEGFR and MEK1 suppressed compensatory MAPK reactivation and downregulated the PI3K-Akt pathway, including the survival-associated genes PPP2R3B and ATF6B, and produced significant tumor growth suppression in vivo. These findings highlight the potential of cabozantinib plus cobimetinib, an already-FDA-approved MEK inhibitor, as a readily translatable combination strategy to overcome cabozantinib resistance in RCC.

## 1. Introduction

Renal cell carcinoma (RCC) is a heterogeneous, aggressive malignancy that represents the most common form of kidney cancer in adults [1]. In clear-cell RCC (ccRCC), inactivation of the von Hippel–Lindau (VHL) tumor-suppressor gene through mutation, deletion, or methylation occurs in approximately 80% of cases and is a well-established driver of tumorigenesis [2]. Loss of VHL function results in the stabilization of hypoxia-inducible factors (HIF1α and HIF2α), and the persistent upregulation of vascular endothelial growth factor (VEGF) and extensive angiogenesis, which are required for tumor growth and progression [3]. The central role of the VHL/HIF/VEGF axis has driven the development of targeted therapies, including multi-tyrosine kinase inhibitors (TKIs), such as sorafenib, sunitinib, bevacizumab, pazopanib, and axitinib, which target VEGF and its receptor (VEGFR), as well as mTOR inhibitors, such as everolimus and temsirolimus [4]. More recently, immune checkpoint inhibitors (ICIs) targeting PD-1, PD-L1, and CTLA-4 have been developed, resulting in a dramatic shift in the treatment landscape for advanced RCC by targeting the tumor microenvironment [5]. Despite advances in targeted therapies, the prognosis for patients with advanced RCC remains poor because of the development of drug resistance and limited effective second-line options [6]. Notably, RCC is highly notorious for its intrinsic and acquired resistance to standard therapies. This resistance is largely driven by tumor heterogeneity, complex metabolic reprogramming, and the rapid activation of compensatory escape pathways that allow tumor cells to bypass targeted single-agent inhibition. Cabozantinib is a multi-targeted TKI that primarily inhibits MET, VEGFR, and AXL. It has become a central agent in the management of advanced RCC [4,7]. The therapeutic potential of cabozantinib extends to other aggressive malignancies; for example, it inhibits tumor progression and cellular invasion in preclinical models of triple-negative breast cancer (TNBC), particularly in MET-dependent contexts [8]; however, a substantial proportion of patients exhibit intrinsic or acquired resistance to cabozantinib, and no established molecular markers reliably predict response or guide subsequent therapy. Accumulating evidence suggests that a major mechanism underlying cabozantinib resistance is the reactivation of downstream mitogenic signaling cascades, particularly the mitogen-activated protein kinase (MAPK) pathway. Consequently, combining cabozantinib with an agent that blocks this specific escape route represents a highly rational approach to disrupting adaptive resistance mechanisms and enhancing therapeutic efficacy.

Synthetic lethality screening using CRISPR/Cas9 genome editing technology enables the unbiased identification of genes whose inhibition enhances the efficacy of existing drugs [9]. This method has the potential to uncover novel combinatorial strategies that can overcome resistance and improve patient outcomes. Previously, we demonstrated that in HNSCC, the combination of mTOR inhibition with the CDK4/6 inhibitor palbociclib disrupts the formation of the eIF4G–CCNE1 mRNA complex, thereby reducing CCNE1 protein expression and reversing adaptive resistance to palbociclib [10]. Consequently, HNSCC cells regain sensitivity to CDK4/6 inhibition, suggesting that the concurrent use of mTOR inhibitors and palbociclib represents a multifaceted therapeutic strategy to prevent palbociclib resistance. Therefore, CRISPR-based approaches may pave the way for more effective combination therapies.

In this study, we conducted a kinome-wide CRISPR/Cas9 synthetic lethal screen in cabozantinib-treated RCC cells with the aim of identifying kinases whose inhibition selectively enhances cabozantinib efficacy and thereby overcomes resistance. Candidate genes were prioritized based on the availability of clinically approved inhibitors in order to facilitate rapid translational application. We then validated the top candidate, MEK1, through pharmacological inhibition with the approved MEK1 inhibitor cobimetinib, using cell proliferation assays, combination index analysis, immunoblotting, RNA sequencing, and xenograft models, to provide preclinical proof-of-concept for cabozantinib plus cobimetinib as a combination strategy in RCC.

## 2. Materials and Methods

### 2.1. Cell Culture

The human RCC cell lines 786-o and A498 RCC were obtained from the American Type Culture Collection (Manassas, VA, USA) and cultured in RPMI 1640 medium supplemented with 10% fetal bovine serum at 37 °C in a 5% CO_2_ atmosphere. These cell lines were validated by short-tandem repeat testing by the Promega Company (Tokyo, Japan). Mycoplasma was also negative. The 786-o and A498 cell lines were selected because both are well-characterized ccRCC cell lines harboring loss-of-function VHL alterations, making them representative models of the predominant molecular subtype of RCC. In addition, both cell lines have been used extensively in prior preclinical studies of cabozantinib and other RTK-targeted therapies, allowing our findings to be interpreted in the context of the existing literature. The use of two independent cell lines with distinct genetic backgrounds also allowed us to confirm that the synergistic effect of cabozantinib plus cobimetinib was not restricted to a single cellular context.

### 2.2. DNA Extraction and CRISPR Screen

We used a human kinome CRISPR pooled library with a gRNA pooled library in lentiCRISPR v2 (one vector system), with 763 genes. The human kinome CRISPR pooled library (Brunello) was a gift from John Doench & David Root (Addgene #75314, Watertown, MA, USA). Four guide RNAs were used for one gene containing 3052 unique sgRNAs targeting 763 kinase genes. The library was amplified according to the Addgene protocol. The functional titer was measured for 786-o cells with the library, and the library was added to cells with a multiplicity of infection of 0.3. The cells were treated with two different groups. 786-o c ells were treated with vehicle or 5 µM cabozantinib until the cells reached different total population doublings. The cabozantinib concentration was determined by other experiments in which the IC50 was 9.73 μM in 786-o. The representation of the sgRNA in the library was maintained at 650 throughout the process. The cells were prepared with three biological replicates. DNA was isolated using the DNeasy Blood and Tissue kit (Qiagen, Holland, The Netherlands) based on the manufacturer’s protocol. The barcode was PCR-amplified and recovered from genomic samples, and the samples were sequenced by NGS to determine the abundance of the different sgRNA probes. The NGS data were analyzed by PinAPL-Py software for the relative abundance of each sgRNA [11]. Significantly altered hit sgRNAs were extracted with an adjusted *p*-value using Sidak’s correction method [11].

### 2.3. Synergy Determination Using the Chou–Talalay Method and Inhibitors

The Chou–Talalay method was used to determine the potential synergistic effects of selected drug combinations [12]. Briefly, 1000 cells per well were seeded into 96-well plates. Cells were treated with single inhibitors or their combinations, using cabozantinib at concentrations of 1–8 μM, and cobimetinib, DCLK1-In-1, and GSK626616 at concentrations of 0.25–1.25 μM, 0.55–5.79 μM, and 3.75–22.63 μM, respectively. Cell viability was measured after a 72 or 96 h treatment using the XTT assay (Roche Applied Science, Tokyo, Japan) based on the manufacturer’s instructions. Combination index (CI) values showing either synergy (<1) or antagonism (>1) were calculated using the Chou–Talalay method. Cabozantinib was obtained from MedChem Express (Monmouth Junction, NJ, USA). The MEK1 inhibitor cobimetinib, DCLK1 inhibitor DCLK1-In-1, FGFR1 inhibitor SSR128129E, and DYRK3 inhibitor GSK626616 were purchased from Selleck (Houston, TX, USA).

### 2.4. Immunoassays

Immunoblot analysis was performed as previously described [13] with diluted anti-p-MET antibodies (1:1000, #3077; Cell Signaling Technology, Danvers, MA, USA), anti-MET antibodies (1:1000, #8198; Cell Signaling Technology), anti-p-Erk1/2 antibodies (1:1000, #4370; Cell Signaling Technology), anti-Erk1/2 antibodies (1:1000, #4695; Cell Signaling Technology), anti-MEK1 antibodies (1:1000, #12671; Cell Signaling Technology), and anti-β-actin antibodies (1:5000, bs-0061R; Bios, Woburn, MA, USA).

### 2.5. In Vivo Tumor Xenograft Model

A mixture containing 100 µL 786-o cells (4 × 106 cells) and 100 µL Matrigel Matrix (Corning, Bedford, MA, USA) was injected subcutaneously into the flanks of female nude mice (BALB/c nu/nu, 6- to 8 weeks old). The mice were separated into the following four groups: vehicle, cabozantinib (5 mg/kg/day, gavage feeding, 5 times a week, 9 days after tumor injection), cobimetinib (2.5 mg/kg/day, gavage feeding, 5 times a week, 9 days after tumor injection), or a combination of the two. The dose was adjusted based on the weight of each mouse, and each volume of injection did not exceed 100 μL. All animal studies using RCC tumor xenografts and orthotropic implantation studies were approved by the animal care review board of Kagoshima University, with protocol #MD23054 (approval date: 29 November 2023), and all experiments adhere to all relevant ethical regulations for animal testing and research.

### 2.6. Gene Enrichment Analysis and RNA Sequencing

Gene expression analysis was carried out using the BioPlanet dataset in GeneCodis 4 (https://genecodis.genyo.es/, accessed on 3 July 2024). The gene expression dataset was derived from the CRISPR screening or RNA sequencing. For RNA sequencing, 786-o was seeded at 1 × 105 cells/well in 6-well plates and treated with vehicle, 10 μM cabozantinib, 2.5 μM cobimetinib, or a combination. After incubation for 6 h and 24 h, total RNA was extracted by lysing the cells in ISOGEN (Nippon Gene, Tokyo, Japan) based on the manufacturer’s protocol. Total RNA was extracted as described above and subjected to mRNA sequencing using the Illumina NovaSeq X plus system (performed by Gene-Nex, Tokyo, Japan).

### 2.7. Statistical and in Silico Analysis

The relationships among three variables and numerical values were analyzed using a Bonferroni-adjusted Mann–Whitney U test. Expert Stat View software, version 5.0 (Cary, NC, USA) was used for the analysis. The TCGA database of 522 RCC patients was used to assess clinical relevance; the Kaplan–Meier method was used to evaluate overall survival using data from the OncoLnc dataset (http://www.oncolnc.org/).

## 3. Results

### 3.1. CRISPR/Cas9 Screening Identifies MEK1 as a Synthetic Lethal Mechanism for Cabozantinib in ccRCC

**Figure 1:**
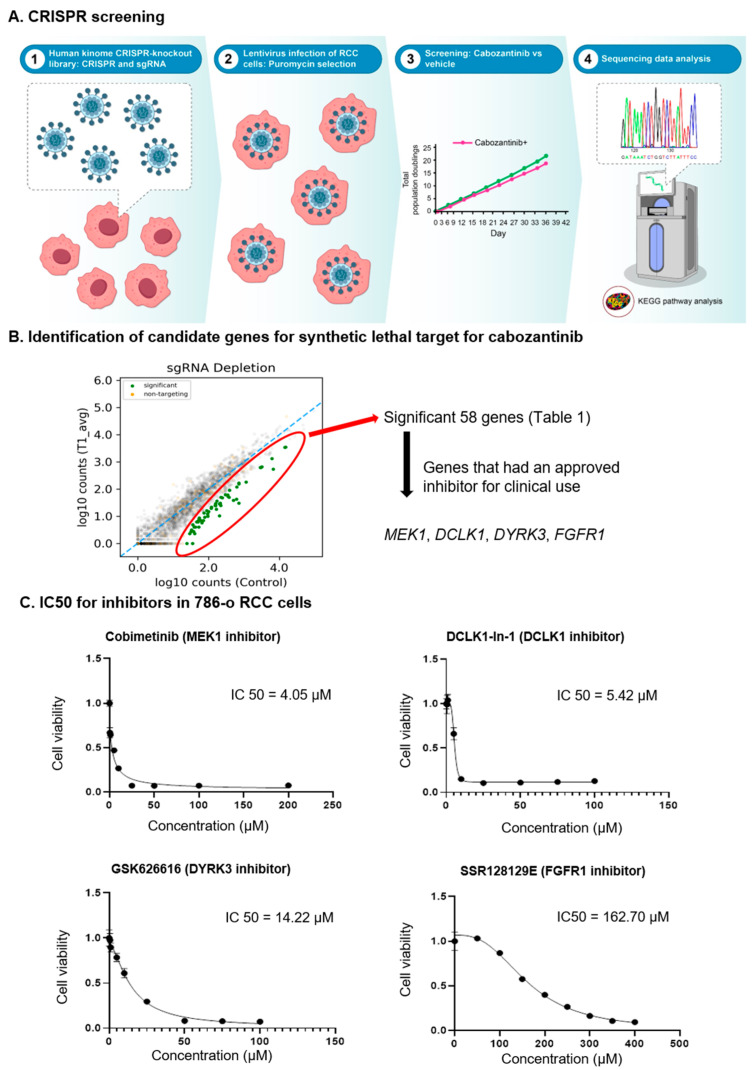
CRISPR/Cas9 Synthetic Lethal Screen.

To identify synthetic lethal targets for cabozantinib in ccRCC, CRISPR screening was conducted using a human kinome-wide CRISPR library, which targeted 763 genes consisting of four sgRNAs for each gene. Initially, we generated Cas9-expressing 786-o ccRCC cells with kinome library and treated 786-o-Cas9-kinome cells with vehicle or cabozantinib until the total population doubling reached 19. After extracting DNA from these cells, PCR was used to amplify the barcodes and next-generation sequencing (NGS) to identify depleted sgRNAs in cabozantinib-treated cells compared with vehicle-treated cells (Figure 1A). NGS revealed 58 significantly depleted sgRNAs, which are candidates for a synthetic lethal target with cabozantinib (Table 1). The results from NGS with sgRNA counts, fold-change, and *p*-values are listed in Supplementary Table S1, which includes 3152 sgRNAs (4 sgRNAs for each gene and 100 non-targeting control sgRNAs). To elucidate the molecular mechanisms underlying the putative target synthetic lethal target genes for cabozantinib, RNA sequencing and a subsequent pathway analysis were conducted. KEGG pathway analysis revealed that the differentially expressed genes were significantly enriched in several critical signaling networks (Supplementary Table S2). Of the 58 sgRNAs, we selected key candidate genes based on whether the target genes had an approved inhibitor for clinical use, because this study aimed to identify established molecules that are available for clinical use. Consequently, MEK1 (MAP2K1), DCLK1, DYRK3, and FGFR1 were selected for further analysis (Figure 1B).

### 3.2. Combination of Cabozantinib and Cobimetinib (MEK1 Inhibitor) Exhibits Synergy in ccRCC Cells In Vitro

**Figure 2:**
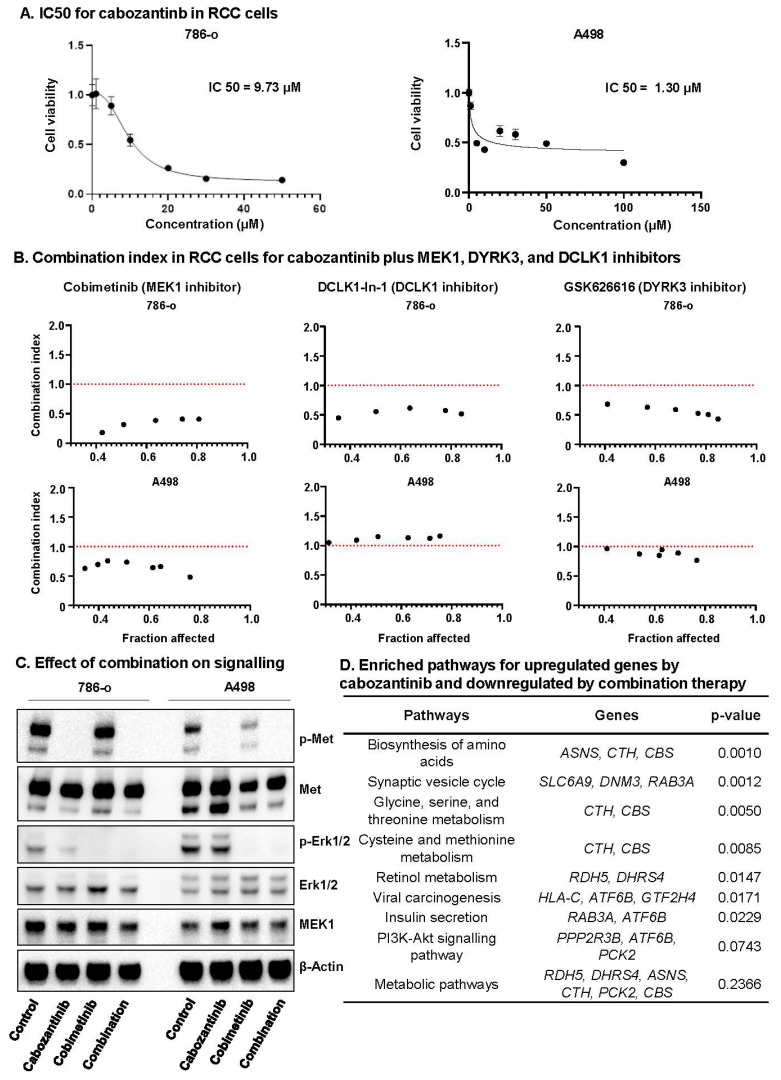
Synergistic effects of cabozantinib and cobimetinib in RCC cell lines.

Based on the results of CRISPR/Cas9 screening, four genes (MEK1, DCLK1, DYRK3, and FGFR1) were selected as candidates for synthetic lethal targets for cabozantinib in ccRCC. First, we determined the IC50 for the inhibitors of these four genes. As shown in Figure 1C, the IC50 for the MEK1 inhibitor in 786-o cells was 4.05 μM, while it was 5.42 μM for the DCLK1 inhibitor, 14.22 μM for the DYRK3 inhibitor, and 162.70 μM for the FGFR1 inhibitor. Because of its high IC50 value in ccRCC cells, we excluded the FGFR1 inhibitor from further analysis. Next, to validate the synthetic lethality, cell proliferation assays were performed to obtain combination index (CI) values for cabozantinib plus DYRK3, DCLK1, and MEK1 inhibitors. As shown in Figure 2A,B, all three inhibitors had a synergistic effect with cabozantinib, consistent with the results of the CRISPR screening. Among the three, the combination index (CI) values for cobimetinib were consistently the lowest across the affected fraction of both 786-o and A498 cells, indicating the strongest and most reproducible degree of synergy with cabozantinib. Because the MEK1 inhibitor showed the strongest synergistic effect in both cell lines, and because cobimetinib is already an FDA-approved agent, offering a direct path toward clinical translation through drug repositioning, we focused on the MEK1 inhibitor, cobimetinib, in subsequent experiments.

### 3.3. RNA Sequencing and Gene Enrichment Analysis to Identify the Mechanism Underlying the Effect of Cabozantinib Plus Cobimetinib

To confirm the basis for the synergistic effect in terms of signaling, a Western blot analysis was performed. As shown in Figure 2C, the combination with cabozantinib suppressed p-MET, whereas little effect was observed with cobimetinib alone. Furthermore, p-ERK was more strongly suppressed by cobimetinib and the combination compared with cabozantinib alone. Thus, the combination suppressed p-MET and p-ERK simultaneously. This inhibition of signaling may be responsible for the synergistic effect of cabozantinib and cobimetinib.

To evaluate the effect of synergy on gene expression, RNA sequencing was performed on 786-o cells treated with control, cabozantinib, cobimetinib, and the combination. The genes were filtered based on the following criteria: (1) expression ≥ 1 in the control to identify genes with relatively high expression under normal conditions; (2) upregulation in response to cabozantinib at 6 and 24 h relative to the control (log_2_ fold-change > 0) to detect genes consistently upregulated by cabozantinib; and (3) downregulated genes resulting from a combination treatment relative to cabozantinib at both time points (log_2_ fold-change <− 0.5) to identify genes that were upregulated by cabozantinib, but downregulated by the combination. A total of 57 genes were detected (Supplementary Table S3). KEGG pathway analysis was conducted on the 57 genes identified using these criteria. Among them, genes associated with the PI3K-Akt signaling pathway were one of the enriched pathways in the cabozantinib and cobimetinib combination (Figure 2D), which suggests that the PI3K-Akt pathway is upregulated by cabozantinib alone, but suppressed by the drug combination. Using the TCGA database for KIRC, we found that high expression of PPP2R3B and ATF6B—two key genes in the PI3K-Akt signaling pathway—was significantly associated with reduced overall survival compared with the low-expression group (Supplementary Figure S1). These results, combined with our functional data, suggest that the dual inhibition of MET/VEGFR and MEK/ERK axes suppresses a broad range of survival signals, including the PI3K-Akt pathway, which otherwise confer resistance.

### 3.4. Combination Therapy with Cabozantinib and Cobimetinib Is Effective Against ccRCC Xenograft Models

**Figure 3:**
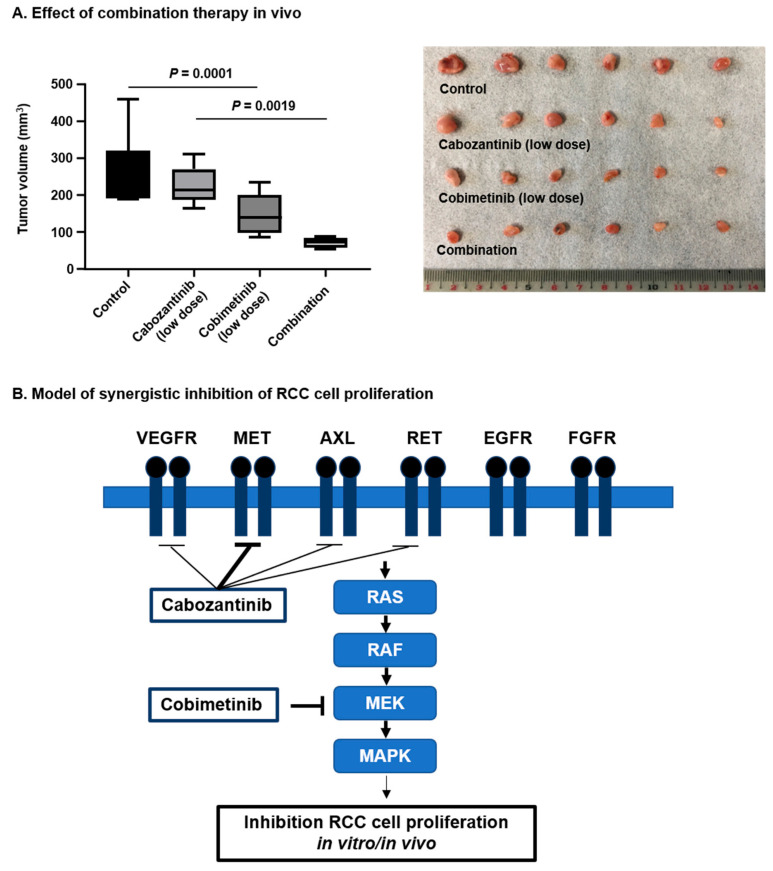
In vivo efficacy of the cabozantinib and cobimetinib combination.

Next, we asked whether the combination of cabozantinib and cobimetinib was effective in vivo. Using a 786-o xenograft model, cabozantinib (5 mg/kg/day), cobimetinib (2.5 mg/kg/day), or a combination was administered five times per week, nine days after tumor cells were injected. A relatively low dose of cabozantinib and cobimetinib was administered to assess synergy. Low-dose cabozantinib alone did not inhibit tumor growth; however, the combination significantly inhibited tumor growth compared with the control and cabozantinib groups (Figure 3A).

Based on these results, we proposed a model in which cobimetinib prevents compensatory MAPK activation, which is typically induced by cabozantinib monotherapy, leading to the synergistic inhibition of RCC cell proliferation (Figure 3B).

A. CRISPR Screening: RCC cells (786-o) were transduced with Cas9 and human kinome CRISPR-knockout library targeting 763 genes. Following infection and selection, the cells were treated with either vehicle or cabozantinib (5 µM). DNA was isolated from these cells and subjected to NGS. Screening outcomes showed the total number of depleted sgRNAs (*n* = 3152).

B. Identification of Candidate Genes for Synthetic Lethal Target for Cabozantinib: Significant hits were identified (*n* = 58), and four genes that had an approved inhibitor for clinical use were extracted—MEK1, DCLK1, DYRK3, and FGFR1.

C. IC50 for Inhibitors in 786-o RCC Cells: The IC50 for the MEK1 inhibitor in 786-o cells was 4.05 μM, while it was 5.42 μM for the DCLK1 inhibitor, 14.22 μM for the DYRK3 inhibitor, and 162.70 μM for the FGFR1 inhibitor.

A. IC50 for Cabozantinib in RCC Cells: The IC50 for cabozantinib was 9.73 μM in 786-o cells and 1.30 μM in A498 cells.

B. Combination Index in RCC Cells for Cabozantinib plus MEK1, DYRK3, and DCLK1 Inhibitors: Combination index (CI) curves for 786-o and A498 RCC cells treated with various doses of cabozantinib and the MEK inhibitor cobimetinib. CI values below 1 indicate synergy.

C. Effect of Combination on Signaling: Western blot showed that combination with cabozantinib suppressed p-MET whereas few effects were observed with cobimetinib alone. p-ERK was more strongly suppressed by cobimetinib and the combination compared with cabozantinib alone. β-actin served as the loading control.

D. Enriched Pathways for Genes Upregulated by Cabozantinib and Downregulated by Combination Therapy: RNA sequencing and pathway analysis showed several enriched pathways associated with cabozantinib resistance and the effects of combination therapy.

A. Effect of Combination Therapy In Vivo: Tumor size and image in xenograft studies of 786-o tumors treated with vehicle, cabozantinib (5 mg/kg/day), cobimetinib (2.5 mg/kg/day), or the combination. The mice were separated into the following four groups: vehicle, cabozantinib, cobimetinib, or a combination of cabozantinib and cobimetinib.

B. Model of Synergistic Inhibition of RCC Cell Proliferation: Schematic showing the simultaneous inhibition of MET/VEGFR and MEK. Cabozantinib-mediated RTK inhibition triggers compensatory MAPK signaling, which is blocked by cobimetinib. This dual blockade results in the inhibition of RCC cell proliferation.

## 4. Discussion

In this study, using a kinome-wide CRISPR/Cas9 screen, we identified MEK1 as a target to maximize the antitumor efficacy of cabozantinib in ccRCC. Cabozantinib is a multi-kinase inhibitor targeting MET, VEGFR2, and AXL. It is a cornerstone of RCC therapy, where it is used as second-line monotherapy and first-line treatment in combination with ICIs [4]. Its clinical importance is rapidly expanding beyond RCC. For example, the recent CONTACT-02 trial for metastatic castration-resistant prostate cancer indicated that cabozantinib plus atezolizumab resulted in a 35% reduction in the risk of disease progression or death [14]; however, a challenge remains. Once cabozantinib fails, subsequent therapies are extremely limited. Therefore, identifying strategic combinations to prevent or overcome resistance is of paramount importance. Our screening results indicate that MEK1 is a key target because of its vital role in the MAPK/ERK pathway and the immediate clinical potential offered by approved MEK inhibitors. Although this study focused on MEK1 because it shows the strongest and most consistent synergy with cabozantinib and is an approved inhibitor with immediate clinical availability, DCLK1 and DYRK3 also demonstrated reproducible synergistic effects with cabozantinib in both cell lines (Figure 2B) and represent additional candidates that warrant further mechanistic and in vivo validation in future studies. Such studies may reveal complementary or alternative combinatorial strategies for overcoming cabozantinib resistance in RCC, particularly in tumors where MEK/ERK signaling is not the dominant resistance mechanism.

Our results demonstrate that combining cabozantinib with the MEK inhibitor cobimetinib translates genetic insights into a potential therapeutic strategy. In 786-o and A498 RCC cells, the combination yielded robust synergy and enhanced cytotoxic effects compared with monotherapy. This is particularly significant because recent evidence suggests that cabozantinib monotherapy triggers the compensatory activation of downstream survival signals, such as the RAS/RAF/MEK/ERK cascade, which reduces drug effectiveness [15]. The simultaneous blockade of MET and its downstream effectors not only reinforces antitumor activity, but also mitigates these adaptive resistance mechanisms. Moreover, our xenograft studies corroborated these results, showing significant tumor suppression in vivo. A trend toward weight loss was observed in the combination group; however, there was no significant difference among groups (Supplementary Figure S2). This highlights the need for the careful optimization of dosage and administration intervals during clinical translation. Because cobimetinib is already FDA-approved for melanoma [16], this combination strategy offers a feasible approach for rapid clinical use in RCC through drug repositioning.

To elucidate the molecular mechanisms underlying this lethality, RNA sequencing revealed a subset of genes involved in the PI3K-Akt signaling pathway, including PPP2R3B and ATF6B [17,18]. These results suggest crosstalk between the MAPK and PI3K-Akt signaling pathways. These molecules, which are involved in the PI3K-Akt signaling pathway, likely induce cell death or cell-cycle arrest during dual inhibition; however, the precise molecular crosstalk and its generalizability among various RCC subtypes require further study. Beyond intracellular signaling, a major challenge for clinical translation is the establishment of robust biomarkers to guide patient stratification. Preclinical studies in TNBC have demonstrated that MET expression levels are an important determinant of cabozantinib sensitivity, which highlights MET as a potential predictive biomarker [8]. Furthermore, because of the increasing clinical use of cabozantinib in combination with ICIs, it is essential to understand the immunological consequences of MEK inhibition. Recent preclinical and early-phase clinical studies, such as selumetinib combined with pembrolizumab, as well as triple-combination strategies involving BRAF/MEK inhibitors plus anti-PD(L)1 antibodies in melanoma, have demonstrated that MEK inhibition can modulate the tumor microenvironment by altering T-cell infiltration, antigen presentation, and cytokine signaling [19,20]. These results suggest that MEK/ERK pathway inhibition may enhance the efficacy of the immune checkpoint blockade; however, MEK inhibitors can also exert transient immunosuppressive effects depending on dose and schedule, potentially reducing ICI responsiveness. Thus, it is important to determine whether MEK inhibition synergizes with, or interferes with, ICI-based regimens in RCC. Future studies using syngeneic mouse models are necessary to clarify whether MEK inhibitors exert favorable immunomodulatory effects, such as enhancing CD8^+^ T-cell activity, or induce adverse immune alterations that may compromise ICI efficacy. Integrating genomic profiling with functional screening approaches will be essential to dissect the complex interactions within the tumor microenvironment (TME) and to design combination strategies that are biologically rational and clinically feasible. A limitation of this study is that the effect of cabozantinib and cobimetinib combination therapy on non-malignant renal epithelial cells was not directly assessed in vitro. Although clinical experience with cabozantinib has not identified renal toxicity as a common dose-limiting adverse event, and our in vivo xenograft experiments did not reveal overt signs of systemic toxicity beyond a modest, non-significant trend toward weight loss in the combination group (Supplementary Figure S2), future studies incorporating normal renal cell lines or organoid models will be important to more directly evaluate the therapeutic index of this combination strategy.

In conclusion, our work provides compelling preclinical evidence that MEK1 inhibition significantly enhances the antitumor effects of cabozantinib in RCC. These results support the clinical study of combined cabozantinib and cobimetinib therapy as a novel strategy to overcome resistance and enhance outcomes for patients with advanced disease. By addressing the mechanisms of adaptive resistance and identifying potential molecular drivers, such as PPP2R3B and ATF6B, we provide a rational basis for the development of personalized and effective combination therapies, which will advance precision medicine in renal oncology.

## Figures and Tables

**Table 1:** Significant sgRNAs identified by CRISPR screening.

Gene	Fold-Change	*p*-Value
*DYRK3*	0.047	4.84 × 10^−16^
*AKT3*	0.074	3.70 × 10^−13^
*MAP3K15*	0.104	4.67 × 10^−13^
*DCLK1*	0.15	5.67 × 10^−13^
*CAMK1D*	0.137	2.28 × 10^−12^
*TESK1*	0.174	1.84 × 10^−9^
*MAPKAPK3*	0.12	2.86 × 10^−9^
*PIK3R3*	0.055	3.54 × 10^−9^
*GK2*	0.041	8.83 × 10^−9^
*PKDCC*	0.136	1.21 × 10^−8^
*PIM2*	0.216	1.48 × 10^−8^
*LIMK1*	0.085	1.59 × 10^−8^
*IP6K2*	0.157	2.05 × 10^−8^
*PFKL*	0.206	2.16 × 10^−8^
*CDK2*	0.067	2.27 × 10^−8^
*EIF2AK1*	0.211	2.33 × 10^−8^
*TESK2*	0.028	3.19 × 10^−8^
*Non-Targeting_Control_081*	0.058	3.36 × 10^−8^
*MAPK4*	0.109	4.58 × 10^−8^
*CDK12*	0.101	9.16 × 10^−8^
*CKS1B*	0.147	9.25 × 10^−8^
*NME8*	0.041	1.02 × 10^−7^
*IRAK4*	0.105	1.40 × 10^−7^
*MYLK4*	0.073	1.66 × 10^−7^
*EPHA1*	0.116	2.23 × 10^−7^
*Non-Targeting_Control_013*	0.217	2.53 × 10^−7^
*HIPK2*	0.046	2.76 × 10^−7^
*ULK3*	0.129	3.12 × 10^−7^
*PRPS1*	0.235	3.55 × 10^−7^
*FUK*	0.191	3.65 × 10^−7^
*BRSK2*	0.142	3.78 × 10^−7^
*DGUOK*	0.095	4.50 × 10^−7^
*ALDH18A1*	0.202	4.74 × 10^−7^
*MAPK1*	0.08	4.77 × 10^−7^
*TPR*	0.114	5.02 × 10^−7^
*PHKG1*	0.13	5.61 × 10^−7^
*VRK2*	0.17	6.43 × 10^−7^
*PIK3CB*	0.109	7.23 × 10^−7^
*PXK*	0.212	7.30 × 10^−7^
*SH3BP4*	0.075	7.77 × 10^−7^
*SYK*	0.23	7.84 × 10^−7^
*NEK8*	0.107	8.10 × 10^−7^
*PI4K2A*	0.079	8.50 × 10^−7^
*CSNK1G3*	0.159	9.85 × 10^−7^
*CKMT1B*	0.205	1.09 × 10^−6^
*LAMTOR3*	0.032	1.14 × 10^−6^
*MKNK1*	0.158	1.49 × 10^−6^
*RPS6KA2*	0.229	1.84 × 10^−6^
*ERN2*	0.176	1.99 × 10^−6^
*KSR2*	0.189	2.08 × 10^−6^
*EXOSC10*	0.216	2.13 × 10^−6^
*MYT1*	0.045	2.31 × 10^−6^
*DAPK3*	0.067	2.35 × 10^−6^
*MAP2K1*	0.138	2.37 × 10^−6^
*PRKAR2B*	0.165	2.41 × 10^−6^
*CLK4*	0.204	2.63 × 10^−6^
*PRKG1*	0.24	2.94 × 10^−6^
*FGFR1*	0.171	3.46 × 10^−6^

## Data Availability

The original contributions presented in this study are included in the article/Supplementary Materials. Further inquiries can be directed to the corresponding author.

## Supplementary Materials

The following supporting information can be downloaded at: https://www.mdpi.com/article/10.3390/genes17070789/s1, Figure S1: Overall survival in renal clear-cell carcinoma (KIRC) relative to PPP2R3B and ATF6B expression; Figure S2: Mouse weight in the xenograft assay; Figure S3: Uncropped WB; Table S1: Results from NGS with sgRNA counts by CRISPR screening for synthetic lethal target for cabozantinib in RCC cells; Table S2: Significantly enriched pathways based on the putative synthetic lethal target genes for cabozantinib; Table S3: Genes upregulated by cabozantinib treatment and downregulated by combination (cabozantinib + cobimetinib) treatment.

## Abbreviations

The following abbreviations are used in this manuscript:

RCC	Renal cell carcinoma
ccRCC	Clear-cell renal cell carcinoma
TKI	Tyrosine kinase inhibitor
CRISPR	Clustered regularly interspaced short palindromic repeats
CI	Combination index
NGS	Next-generation sequencing
VHL	von Hippel–Lindau
TNBC	Triple-negative breast cancer
