# Supplementary material for: MEK1 as a Synthetic Lethal Target with Cabozantinib in Renal Cell Carcinoma: Insights from CRISPR/Cas9 Screening
Source: Genes (Basel). 2026 Jul 12;17(7):789. doi: 10.3390/genes17070789 (PMC13409911; doi:10.3390/genes17070789)
Supplement: Supplementary file 1 [file genes-17-00789-s001.zip › genes-4412660 Supplementary Table 1.pdf]

Supplementary Table 1. Results from NGS with sgRNA counts by CRISPR screening for synthetic lethal target for cabozantinib in RCC cells

Gene	Counts	Control mean	Control stdev	Fold change	p-value	Significant
DYRK3	29.239	720.086	180.254	0.041	4.84E-16	True
AKT3	41.279	616.871	154.703	0.067	3.70E-13	True
MAP3K15	9.460	203.072	52.248	0.047	4.67E-13	True
DCLK1	541.358	6749.311	1672.699	0.080	5.67E-13	True
CAMK1D	37.409	500.595	125.918	0.075	2.28E-12	True
TESK1	24.079	220.532	56.573	0.109	1.84E-09	True
MAPKAPK3	17.200	164.693	42.739	0.104	2.86E-09	True
PIK3R3	49.019	380.304	96.137	0.129	3.54E-09	True
GK2	0.000	34.521	10.370	0.028	8.83E-09	True
PKDCC	4.730	63.605	17.649	0.074	1.21E-08	True
PIM2	181.456	1148.925	286.410	0.158	1.48E-08	True
LIMK1	9.030	94.663	25.372	0.095	1.59E-08	True
IP6K2	27.519	202.342	52.067	0.136	2.05E-08	True
PFKL	29.239	213.496	54.830	0.137	2.16E-08	True
CDK2	1.290	39.719	11.678	0.032	2.27E-08	True
EIF2AK1	53.319	354.830	89.830	0.150	2.33E-08	True
TESK2	3.010	52.253	14.817	0.058	3.19E-08	True
Non-Targeting_Control_081	6.020	70.969	19.483	0.085	3.36E-08	True
MAPK4	1.720	37.297	11.069	0.046	4.58E-08	True
CDK12	11.180	93.374	25.052	0.120	9.16E-08	True
CKS1B	8.170	76.597	20.883	0.107	9.25E-08	True
NME8	23.219	157.480	40.952	0.147	1.02E-07	True
IRAK4	60.199	346.124	87.674	0.174	1.40E-07	True
MYLK4	13.760	99.538	26.582	0.138	1.66E-07	True
EPHA1	118.247	624.357	156.556	0.189	2.23E-07	True
Non-Targeting_Control_013	24.079	151.588	39.491	0.159	2.53E-07	True
HIPK2	6.020	59.493	16.624	0.101	2.76E-07	True
ULK3	1.720	31.193	9.529	0.055	3.12E-07	True
PRPS1	659.176	3212.671	797.261	0.205	3.55E-07	True
FUK	18.490	117.916	31.143	0.157	3.65E-07	True
BRSK2	617.897	2995.459	743.493	0.206	3.78E-07	True
DGUOK	3.010	41.399	12.100	0.073	4.50E-07	True
ALDH18A1	27.949	158.497	41.204	0.176	4.74E-07	True
MAPK1	0.000	23.497	7.571	0.041	4.77E-07	True
TPR	626.496	2973.645	738.094	0.211	5.02E-07	True
PHKG1	46.869	245.452	62.745	0.191	5.61E-07	True
VRK2	1328.241	6154.338	1525.423	0.216	6.43E-07	True
PIK3CB	623.486	2877.704	714.345	0.217	7.23E-07	True
PXK	20.640	120.698	31.833	0.171	7.30E-07	True
SH3BP4	1.290	28.534	8.855	0.045	7.77E-07	True
SYK	152.647	720.975	180.474	0.212	7.84E-07	True
NEK8	5.160	49.254	14.068	0.105	8.10E-07	True
PI4K2A	60.629	300.863	76.467	0.202	8.50E-07	True
CSNK1G3	5.590	48.395	13.853	0.116	9.85E-07	True
CKMT1B	6.880	52.733	14.937	0.130	1.09E-06	True
LAMTOR3	3.010	37.961	11.237	0.079	1.14E-06	True
MKNK1	2.150	32.000	9.734	0.067	1.49E-06	True
RPS6KA2	3515.604	14966.759	3706.792	0.235	1.84E-06	True
ERN2	7.740	54.437	15.363	0.142	1.99E-06	True
KSR2	5.160	45.196	13.052	0.114	2.08E-06	True
EXOSC10	165.976	721.091	180.503	0.230	2.13E-06	True
MYT1	15.050	88.358	23.806	0.170	2.31E-06	True
DAPK3	35.259	172.653	44.712	0.204	2.35E-06	True
MAP2K1	3.870	35.403	10.592	0.109	2.37E-06	True
PRKAR2B	3452.395	14390.579	3564.169	0.240	2.41E-06	True
CLK4	48.589	225.044	57.691	0.216	2.63E-06	True
PRKG1	89.438	391.319	98.864	0.229	2.94E-06	True
FGFR1	12.040	72.866	19.955	0.165	3.46E-06	True
Non-Targeting_Control_007	0.860	18.358	6.246	0.047	4.55E-06	False
GAK	0.860	18.094	6.177	0.048	5.15E-06	False
CIT	3.870	32.449	9.847	0.119	5.72E-06	False
IPMK	0.000	17.340	5.980	0.055	7.40E-06	False
Non-Targeting_Control_008	0.000	17.335	5.979	0.055	7.42E-06	False
DGKQ	22.360	106.529	28.318	0.210	7.71E-06	False
MAP3K6	98.038	387.925	98.024	0.253	9.80E-06	False
POMK	153.937	589.364	147.893	0.261	1.05E-05	False
PDGFRA	7.740	46.613	13.407	0.166	1.06E-05	False
DCLK2	13.330	69.688	19.164	0.191	1.06E-05	False
JAK3	5.160	38.602	11.398	0.134	1.08E-05	False
PAPSS1	44.719	184.150	47.560	0.243	1.15E-05	False
PRKAB1	279.494	1041.779	259.887	0.268	1.22E-05	False

FASTK	10.750	57.290	16.075	0.188	1.26E-05	False
IKBKB	77.398	300.469	76.369	0.258	1.41E-05	False
TTK	68.369	266.496	67.957	0.257	1.50E-05	False
CHEK2	21.930	96.500	25.828	0.227	1.55E-05	False
HUNK	728.404	2611.561	648.465	0.279	1.68E-05	False
PRKACB	14.190	70.400	19.341	0.202	1.69E-05	False
PFKFB4	96.318	363.207	91.903	0.265	1.70E-05	False
RIOK3	32.249	135.129	35.411	0.239	1.71E-05	False
PRKD3	80.408	304.413	77.346	0.264	1.83E-05	False
LCK	48.589	189.333	48.845	0.257	2.04E-05	False
ROCK2	15.480	72.246	19.800	0.214	2.21E-05	False
TESK1	0.430	15.137	5.402	0.028	2.23E-05	False
GSAP	3.440	27.663	8.634	0.124	2.65E-05	False
MAPK9	288.524	990.959	247.307	0.291	3.22E-05	False
STK25	0.000	14.424	5.213	0.065	3.25E-05	False
TRIB2	0.000	14.297	5.179	0.065	3.47E-05	False
PAN3	76.108	272.611	69.471	0.279	3.79E-05	False
IKBKB	25.799	102.095	27.217	0.253	3.93E-05	False
TSSK2	15.910	68.255	18.807	0.233	4.05E-05	False
AURKB	18.060	78.010	21.234	0.232	4.25E-05	False
SIK3	582.207	1932.683	480.419	0.301	4.31E-05	False
EEF2K	9.890	47.432	13.612	0.209	4.36E-05	False
AURKA	85.998	298.820	75.961	0.288	4.37E-05	False
HUNK	3.870	26.135	8.245	0.148	4.45E-05	False
TAOK1	381.402	1268.177	315.930	0.301	4.47E-05	False
ULK1	89.438	308.803	78.433	0.290	4.91E-05	False
MKNK2	0.000	13.585	4.989	0.069	5.10E-05	False
SRM	52.889	187.121	48.296	0.283	5.27E-05	False
MLKL	15.910	65.179	18.041	0.244	6.54E-05	False
DLG1	28.809	106.634	28.344	0.270	6.79E-05	False
IPMK	89.438	299.617	76.158	0.299	6.90E-05	False
PHKG2	15.050	64.425	17.853	0.234	7.37E-05	False
VRK3	78.258	263.285	67.161	0.297	7.38E-05	False
TESK2	116.097	381.681	96.478	0.304	7.59E-05	False
MST1	8725.800	27259.106	6749.560	0.320	7.81E-05	False
PHKG1	0.430	12.785	4.775	0.034	7.94E-05	False
CPNE3	0.860	12.684	4.748	0.068	8.40E-05	False
LCK	89.438	294.117	74.796	0.304	8.50E-05	False
PLK5	36.119	129.308	33.968	0.279	8.56E-05	False
PCK1	531.468	1662.275	413.484	0.320	8.61E-05	False
DSTYK	4.730	27.522	8.598	0.172	9.19E-05	False
CIB4	15961.683	48822.931	12087.327	0.327	9.91E-05	False
SLAMF6	0.430	12.358	4.660	0.035	1.01E-04	False
PRKAA1	14.620	58.854	16.465	0.248	1.10E-04	False
TSSK1B	142.757	446.021	112.407	0.320	1.16E-04	False
TAF9	128.567	402.861	101.721	0.319	1.19E-04	False
STK26	7.740	36.458	10.858	0.212	1.20E-04	False
C17orf75	33.969	115.601	30.568	0.294	1.27E-04	False
GSK3A	30.529	106.313	28.264	0.287	1.30E-04	False
GALK1	41.279	138.495	36.246	0.298	1.39E-04	False
HIPK3	110.078	342.761	86.841	0.321	1.43E-04	False
PSKH1	22.790	81.328	22.059	0.280	1.45E-04	False
PIK3CD	17.630	66.281	18.316	0.266	1.45E-04	False
GAK	71.808	225.981	57.923	0.318	1.52E-04	False
TTN	2.150	19.224	6.471	0.112	1.56E-04	False
SGK1	646.706	1911.834	475.258	0.338	1.59E-04	False
GSAP	1.290	15.568	5.515	0.083	1.60E-04	False
EIF2AK3	0.000	11.550	4.441	0.080	1.61E-04	False
NEK5	0.430	11.501	4.428	0.037	1.66E-04	False
CAD	0.860	11.493	4.426	0.075	1.67E-04	False
PANK4	0.000	11.486	4.424	0.080	1.67E-04	False
AKT2	3.440	22.398	7.290	0.154	1.69E-04	False
CD2	144.047	436.329	110.007	0.330	1.71E-04	False
KALRN	0.000	11.430	4.408	0.080	1.73E-04	False
ALDH18A1	9.460	41.146	12.037	0.230	1.73E-04	False
SRPK1	3.440	22.322	7.270	0.154	1.74E-04	False
PANK1	3.870	22.176	7.232	0.175	1.84E-04	False
MAP3K9	225.315	665.242	166.677	0.339	1.92E-04	False
Non-Targeting_Control_026	253.694	745.591	186.568	0.340	1.94E-04	False
EPHB4	73.098	226.678	58.095	0.322	1.94E-04	False
PIP5K1A	0.430	11.103	4.319	0.039	2.10E-04	False
JAK1	1827.460	5208.851	1291.383	0.351	2.25E-04	False
ADRBK1	13.330	51.771	14.697	0.257	2.25E-04	False

CDK2	3.010	21.535	7.068	0.140	2.34E-04	False
PRKAA1	9.460	39.736	11.683	0.238	2.39E-04	False
PKD2	27.949	91.075	24.481	0.307	2.56E-04	False
STK31	14.620	53.896	15.228	0.271	2.59E-04	False
STK32B	353.452	1001.266	249.858	0.353	2.74E-04	False
Non-Targeting_Control_029	86.428	255.649	65.270	0.338	2.75E-04	False
CDK6	17.630	62.003	17.250	0.284	2.80E-04	False
HIPK4	64.929	193.806	49.953	0.335	2.80E-04	False
Non-Targeting_Control_031	0.000	10.611	4.184	0.086	2.82E-04	False
Non-Targeting_Control_017	555.978	1560.767	388.357	0.356	2.83E-04	False
MAPK3	7054.426	19639.124	4863.359	0.359	2.85E-04	False
FASTKD3	0.000	10.585	4.177	0.086	2.86E-04	False
WNK2	106.638	310.137	78.763	0.344	2.89E-04	False
TYK2	132.437	381.777	96.501	0.347	2.94E-04	False
KSR2	0.860	10.504	4.155	0.082	3.01E-04	False
STK17A	11.610	44.474	12.871	0.261	3.03E-04	False
DCAKD	11.610	44.435	12.861	0.261	3.06E-04	False
UCK2	11.180	44.260	12.818	0.253	3.17E-04	False
DDR1	100.188	289.753	73.716	0.346	3.30E-04	False
PIM3	8.600	35.480	10.612	0.242	3.30E-04	False
TPR	583.927	1615.262	401.846	0.362	3.31E-04	False
EPHA8	10.750	41.042	12.010	0.262	3.43E-04	False
SIK3	7.310	32.201	9.784	0.227	3.66E-04	False
MAP3K11	533.618	1463.336	364.239	0.365	3.69E-04	False
HIPK4	18.060	62.931	17.481	0.287	3.74E-04	False
STK24	151.357	424.397	107.053	0.357	3.78E-04	False
CHKA	50.739	150.281	39.167	0.338	3.78E-04	False
PFKFB2	3.870	20.220	6.728	0.191	3.87E-04	False
ROCK1	6.020	28.914	8.952	0.208	4.11E-04	False
CALM3	102.768	288.818	73.484	0.356	4.16E-04	False
ADRBK2	3.870	20.016	6.676	0.193	4.18E-04	False
MAPK12	142.327	394.701	99.701	0.361	4.35E-04	False
GRK7	1.720	13.535	4.976	0.127	4.40E-04	False
EPHA7	197.366	539.589	135.571	0.366	4.55E-04	False
MYLK2	57.619	165.919	43.043	0.347	4.65E-04	False
PANK4	394.731	1058.305	263.978	0.373	4.84E-04	False
PRKX	0.000	9.731	3.941	0.093	4.84E-04	False
NMRK2	9.890	36.727	10.926	0.269	4.86E-04	False
CDK5R1	0.860	9.612	3.908	0.089	5.22E-04	False
PDK2	37.409	111.022	29.432	0.337	5.32E-04	False
MYLK2	11.180	41.770	12.193	0.268	5.36E-04	False
PANK4	1824.020	4796.957	1189.425	0.380	5.39E-04	False
CKMT1A	2055.355	5388.455	1335.841	0.381	5.54E-04	False
CALM3	40.419	118.174	31.207	0.342	5.67E-04	False
MORN2	0.860	9.479	3.871	0.091	5.68E-04	False
AK3	95.028	261.792	66.792	0.363	5.73E-04	False
OBSCN	18.060	59.958	16.740	0.301	5.91E-04	False
TTK	2017.946	5231.540	1296.999	0.386	6.22E-04	False
ALPK3	82.558	225.742	57.863	0.366	6.30E-04	False
MPP5	0.000	9.311	3.824	0.097	6.32E-04	False
MARK3	15.910	51.534	14.638	0.309	6.43E-04	False
EPHB2	0.430	9.227	3.800	0.047	6.67E-04	False
IGFN1	17.630	56.474	15.871	0.312	6.78E-04	False
RNASEL	457.940	1185.849	295.550	0.386	6.82E-04	False
AK7	1.290	12.648	4.738	0.102	6.96E-04	False
FER	14.190	48.405	13.855	0.293	7.04E-04	False
UCK2	113.948	302.541	76.882	0.377	7.06E-04	False
RNASEL	21.500	66.531	18.378	0.323	7.14E-04	False
PDIK1L	111.798	296.962	75.501	0.376	7.17E-04	False
MAPK1	19.350	61.261	17.065	0.316	7.24E-04	False
CDK11A	57.189	157.667	40.998	0.363	7.77E-04	False
OSR1	42.139	119.383	31.506	0.353	7.87E-04	False
HK3	0.000	8.953	3.723	0.100	7.95E-04	False
IPPK	46.009	129.342	33.976	0.356	7.99E-04	False
SH3BP5	21.070	65.482	18.117	0.322	8.28E-04	False
LYN	15.050	50.049	14.266	0.301	8.37E-04	False
CDK13	198.226	510.668	128.412	0.388	8.48E-04	False
PDGFRL	622.196	1566.599	389.800	0.397	8.99E-04	False
MAP4K2	111.798	290.305	73.852	0.385	9.02E-04	False
TRIB3	4.730	20.814	6.882	0.227	9.20E-04	False
KALRN	25.799	74.363	20.327	0.347	9.75E-04	False
NRK	11.610	39.007	11.499	0.298	9.77E-04	False
SGK3	15.050	49.163	14.045	0.306	9.82E-04	False

PHKA2	3.010	17.851	6.113	0.169	9.90E-04	False
EIF2AK3	2312.490	5723.794	1418.849	0.404	1.01E-03	False
BMX	143.617	366.102	92.620	0.392	1.01E-03	False
TRIM33	14.620	46.474	13.372	0.315	1.01E-03	False
PKLR	36.119	101.075	26.964	0.357	1.05E-03	False
CPNE3	10.750	36.102	10.769	0.298	1.06E-03	False
PIM1	50.309	135.343	35.464	0.372	1.07E-03	False
MAST2	4.730	20.234	6.732	0.234	1.14E-03	False
LRGUK	10.320	35.708	10.669	0.289	1.16E-03	False
AURKA	10.750	35.635	10.651	0.302	1.18E-03	False
CAMK4	1.290	11.605	4.456	0.111	1.21E-03	False
PRKCB	41.279	111.393	29.524	0.371	1.25E-03	False
STK39	2233.371	5405.005	1339.938	0.413	1.27E-03	False
C8orf44-SGK3	3.440	17.220	5.949	0.200	1.28E-03	False
CDK10	9.460	32.763	9.927	0.289	1.28E-03	False
MARK1	0.430	8.175	3.503	0.053	1.32E-03	False
TXK	23.219	66.994	18.493	0.347	1.34E-03	False
IPPK	0.000	8.159	3.498	0.109	1.34E-03	False
NEK1	0.860	8.116	3.486	0.106	1.38E-03	False
RIOK2	700.885	1685.338	419.193	0.416	1.41E-03	False
CDK4	0.000	8.074	3.474	0.110	1.42E-03	False
RIOK3	6.450	24.806	7.906	0.260	1.42E-03	False
TRIO	36.119	97.731	26.134	0.370	1.44E-03	False
MAPK3	20.640	59.197	16.550	0.349	1.44E-03	False
CDKL1	40.849	107.290	28.506	0.381	1.44E-03	False
NEK11	61.919	157.360	40.922	0.393	1.46E-03	False
YES1	7.310	27.237	8.526	0.268	1.47E-03	False
PAK7	6.020	24.677	7.873	0.244	1.48E-03	False
ATMIN	0.000	7.969	3.444	0.112	1.52E-03	False
EPHA7	211.985	511.310	128.570	0.415	1.56E-03	False
PHKA2	42.139	110.253	29.241	0.382	1.68E-03	False
CROT	1.290	11.005	4.293	0.117	1.68E-03	False
FGR	684.115	1615.820	401.984	0.423	1.70E-03	False
RET	0.000	7.792	3.393	0.114	1.71E-03	False
CDK5	4.730	19.085	6.434	0.248	1.74E-03	False
STK17B	10.750	33.952	10.227	0.317	1.76E-03	False
Non-Targeting_Control_088	0.860	7.701	3.367	0.112	1.82E-03	False
MAPK7	0.000	7.696	3.365	0.115	1.83E-03	False
ZAP70	28.809	76.323	20.815	0.377	1.86E-03	False
CMPK2	2.150	13.602	4.994	0.158	1.90E-03	False
PACSIN1	0.000	7.637	3.348	0.116	1.90E-03	False
NME1	0.000	7.629	3.346	0.116	1.91E-03	False
MAP3K15	5.160	21.353	7.021	0.242	1.92E-03	False
EPHB4	78.258	192.251	49.567	0.407	1.92E-03	False
STYK1	0.000	7.586	3.334	0.116	1.97E-03	False
FASTKD1	6.450	23.738	7.633	0.272	1.99E-03	False
SH3BP5L	20.210	56.937	15.987	0.355	2.03E-03	False
PEAK1	125.127	297.820	75.713	0.420	2.15E-03	False
PIK3CA	5.160	21.019	6.935	0.245	2.16E-03	False
TTN	85.138	205.182	52.771	0.415	2.24E-03	False
TNIK	1.290	10.486	4.150	0.123	2.25E-03	False
CDK19	0.000	7.332	3.260	0.120	2.34E-03	False
PKDCC	195.216	453.550	114.271	0.430	2.37E-03	False
MPP2	23.649	62.649	17.411	0.377	2.42E-03	False
TTBK2	22.360	60.264	16.816	0.371	2.46E-03	False
PIK3CB	2.580	12.946	4.818	0.199	2.60E-03	False
Non-Targeting_Control_037	0.000	7.181	3.216	0.122	2.60E-03	False
TPD52L3	425.261	964.048	240.646	0.441	2.65E-03	False
NME3	21.500	57.449	16.114	0.374	2.65E-03	False
PIM1	89.438	210.306	54.040	0.425	2.67E-03	False
PDK3	27.949	70.887	19.462	0.394	2.71E-03	False
CMPK1	106.208	247.502	63.253	0.429	2.77E-03	False
DYRK3	4.730	17.718	6.079	0.267	2.93E-03	False
ROR2	59.769	141.519	36.996	0.422	2.94E-03	False
Non-Targeting_Control_086	40.849	99.145	26.485	0.412	2.96E-03	False
MYLK3	6.450	22.416	7.294	0.288	3.03E-03	False
CDKL4	5.590	19.945	6.657	0.280	3.13E-03	False
DCLK2	16.340	44.924	12.984	0.364	3.26E-03	False
PI4K2B	18.920	49.383	14.100	0.383	3.26E-03	False
Non-Targeting_Control_011	48.159	115.551	30.556	0.417	3.30E-03	False
ACVR1B	3.440	14.960	5.355	0.230	3.32E-03	False
IRAK2	1879.489	4125.497	1023.216	0.456	3.32E-03	False
TKFC	271.754	603.231	151.326	0.450	3.37E-03	False

AK5	322.063	714.635	178.904	0.451	3.37E-03	False
AURKB	76.538	176.336	45.624	0.434	3.42E-03	False
FLT3	21.930	55.678	15.672	0.394	3.46E-03	False
CD2	0.000	6.748	3.088	0.129	3.53E-03	False
MAP3K4	3.440	14.796	5.311	0.232	3.56E-03	False
CAMKV	45.579	107.980	28.678	0.422	3.57E-03	False
TRIM27	24.939	61.954	17.238	0.403	3.62E-03	False
MET	5.590	19.517	6.546	0.286	3.64E-03	False
PAK2	11.610	33.128	10.019	0.350	3.70E-03	False
MST1R	3.440	14.629	5.267	0.235	3.83E-03	False
KIAA1804	446.330	972.231	242.671	0.459	3.84E-03	False
CDC42BPA	28.379	70.201	19.292	0.404	3.85E-03	False
ROR2	2.150	12.114	4.594	0.177	3.90E-03	False
MAPK11	43.429	102.501	27.318	0.424	3.93E-03	False
ZAK	2.580	12.082	4.585	0.214	3.97E-03	False
MPP7	0.000	6.539	3.026	0.133	4.09E-03	False
HIPK3	0.000	6.472	3.006	0.134	4.29E-03	False
TAF1L	6.020	21.272	7.000	0.283	4.39E-03	False
ROS1	6.020	21.252	6.995	0.283	4.41E-03	False
AKT1	28.379	69.027	18.999	0.411	4.44E-03	False
MARK2	9.460	27.758	8.659	0.341	4.66E-03	False
MAP3K19	20.640	51.527	14.636	0.401	4.68E-03	False
BMPR1A	1.290	9.183	3.788	0.140	4.71E-03	False
CAMK2B	34.399	81.282	22.047	0.423	4.72E-03	False
PIK3CD	0.860	6.336	2.966	0.136	4.73E-03	False
PRKCD	201.666	433.790	109.379	0.465	4.74E-03	False
HIPK4	4.300	16.452	5.748	0.261	4.79E-03	False
Non-Targeting_Control_096	15.050	40.631	11.907	0.370	4.82E-03	False
PRKCA	0.000	6.308	2.957	0.137	4.83E-03	False
PCK2	0.000	6.302	2.955	0.137	4.85E-03	False
PRKCH	0.430	6.299	2.955	0.068	4.86E-03	False
TSSK3	0.000	6.288	2.951	0.137	4.90E-03	False
FRK	3.010	14.030	5.108	0.215	4.97E-03	False
ALK	13.330	36.140	10.778	0.369	5.01E-03	False
GLYCTK	3.870	13.960	5.089	0.277	5.12E-03	False
TAB1	420.961	886.311	221.402	0.475	5.22E-03	False
BMPR2	404.191	852.137	212.943	0.474	5.26E-03	False
DCK	73.958	161.753	42.011	0.457	5.28E-03	False
PRKACB	14.190	37.989	11.243	0.374	5.32E-03	False
EPHA6	22494.961	46741.674	11572.146	0.481	5.43E-03	False
TBK1	131.147	281.576	71.691	0.466	5.48E-03	False
STK19	6.880	20.589	6.824	0.334	5.49E-03	False
EPHB2	158.237	336.769	85.358	0.470	5.61E-03	False
EPHA10	0.430	6.101	2.895	0.070	5.61E-03	False
GK	193.066	408.211	103.046	0.473	5.74E-03	False
PRKCG	23.649	56.472	15.871	0.419	5.76E-03	False
NME8	2.580	11.303	4.374	0.228	5.84E-03	False
ETNK1	1.720	8.793	3.678	0.196	5.92E-03	False
HCK	1.290	8.779	3.674	0.147	5.97E-03	False
ITK	1.290	8.765	3.670	0.147	6.02E-03	False
PIK3CD	0.430	5.979	2.858	0.072	6.13E-03	False
Non-Targeting_Control_098	459.230	948.855	236.885	0.484	6.24E-03	False
DAPK2	7.310	22.297	7.264	0.328	6.37E-03	False
SCYL2	147.917	309.116	78.510	0.479	6.44E-03	False
AK4	0.000	5.902	2.835	0.145	6.49E-03	False
VRK3	131.577	276.258	70.374	0.476	6.49E-03	False
BMPR2	91.158	194.206	50.052	0.469	6.63E-03	False
ZAK	129.857	271.439	69.181	0.478	6.64E-03	False
AK9	33.539	75.943	20.720	0.442	6.67E-03	False
NME4	129.427	271.060	69.087	0.477	6.73E-03	False
AK4	10.320	28.477	8.841	0.362	6.73E-03	False
ROS1	0.860	5.834	2.814	0.147	6.82E-03	False
PIM1	100.188	211.603	54.361	0.473	6.88E-03	False
PRKAG1	182.316	377.609	95.469	0.483	6.90E-03	False
PAK7	742.164	1510.523	375.919	0.491	6.92E-03	False
Non-Targeting_Control_070	223.595	460.344	115.953	0.486	6.93E-03	False
PFKP	79.118	168.522	43.688	0.469	7.07E-03	False
ANKK1	21.930	50.949	14.491	0.430	7.08E-03	False
IKBKE	0.000	5.781	2.798	0.147	7.09E-03	False
SPHK2	0.000	5.775	2.796	0.148	7.12E-03	False
ADCK5	4.730	15.352	5.458	0.308	7.40E-03	False
FLT1	12.470	32.230	9.792	0.387	7.42E-03	False
STK4	331.953	672.003	168.351	0.494	7.58E-03	False

CAMK1	3086.473	6158.359	1526.418	0.501	7.93E-03	False
STK33	2.580	10.694	4.207	0.241	7.93E-03	False
PDGFRA	8.600	23.592	7.596	0.365	8.13E-03	False
PLXNA3	0.000	5.585	2.738	0.152	8.20E-03	False
GUK1	3.870	12.892	4.804	0.300	8.22E-03	False
CAMK1G	2.150	10.586	4.178	0.203	8.38E-03	False
EPHB1	0.000	5.551	2.728	0.153	8.41E-03	False
PDGFRL	0.000	5.545	2.725	0.153	8.45E-03	False
DDR1	13.330	33.598	10.137	0.397	8.61E-03	False
SRPK2	0.860	5.493	2.710	0.157	8.78E-03	False
EPHA1	2.580	10.481	4.149	0.246	8.84E-03	False
PKN1	179.736	360.690	91.280	0.498	8.93E-03	False
PCK2	1.290	8.081	3.476	0.160	9.03E-03	False
CARD11	301.853	599.142	150.314	0.504	9.12E-03	False
SIK2	2031.276	3979.052	986.966	0.510	9.39E-03	False
CERKL	162.536	325.075	82.462	0.500	9.46E-03	False
CLK3	6.020	18.915	6.390	0.318	9.56E-03	False
CKB	0.000	5.373	2.672	0.157	9.61E-03	False
CDK4	289.384	571.318	143.426	0.507	9.73E-03	False
GK5	0.000	5.353	2.666	0.157	9.76E-03	False
VRK3	45.149	95.705	25.631	0.472	9.92E-03	False
ROCK1	0.860	5.326	2.658	0.161	9.96E-03	False
OXSR1	78.258	159.797	41.526	0.490	1.00E-02	False
MPP4	102.338	206.234	53.031	0.496	1.01E-02	False
KSR2	104.488	209.671	53.883	0.498	1.03E-02	False
MTOR	1.720	7.852	3.410	0.219	1.04E-02	False
WNK1	0.860	5.269	2.640	0.163	1.04E-02	False
CDK7	101.048	203.139	52.265	0.497	1.06E-02	False
FGFR1	68.799	139.414	36.474	0.493	1.06E-02	False
EPHB3	0.000	5.227	2.627	0.161	1.07E-02	False
Non-Targeting_Control_080	570.168	1104.241	275.349	0.516	1.09E-02	False
N4BP2	3.440	12.246	4.630	0.281	1.10E-02	False
SIK1	248.105	484.232	121.867	0.512	1.10E-02	False
CDK11B	9.890	24.463	7.819	0.404	1.12E-02	False
AKT3	14.190	34.261	10.305	0.414	1.14E-02	False
CDK14	38.699	80.553	21.866	0.480	1.15E-02	False
STK16	433.861	835.383	208.795	0.519	1.15E-02	False
IP6K1	45.149	93.901	25.183	0.481	1.15E-02	False
EIF2AK3	1474.438	2816.595	699.218	0.523	1.17E-02	False
AK1	42.139	87.953	23.705	0.479	1.18E-02	False
ADCK1	44.289	91.704	24.637	0.483	1.18E-02	False
ABL2	24.079	53.345	15.090	0.451	1.19E-02	False
RAB11FIP5	71.378	142.870	37.330	0.500	1.21E-02	False
RPS6KA1	182.316	353.697	89.549	0.515	1.21E-02	False
PBK	0.860	5.068	2.577	0.170	1.21E-02	False
ULK2	11.180	28.079	8.740	0.398	1.22E-02	False
LCK	18.490	41.551	12.138	0.445	1.24E-02	False
TIE1	25.369	54.959	15.493	0.462	1.24E-02	False
MAP2K1	0.860	5.031	2.565	0.171	1.25E-02	False
KDR	0.000	5.030	2.565	0.166	1.25E-02	False
CSNK2B	0.000	5.028	2.565	0.166	1.25E-02	False
CRKL	3.010	11.948	4.549	0.252	1.26E-02	False
ALPK2	5.590	16.082	5.651	0.348	1.26E-02	False
CAD	0.000	5.011	2.559	0.166	1.27E-02	False
NEK3	8.170	22.053	7.201	0.370	1.27E-02	False
PIK3C2B	11.610	27.926	8.701	0.416	1.27E-02	False
MKNK2	0.000	4.968	2.545	0.168	1.31E-02	False
PNCK	4.730	13.909	5.076	0.340	1.32E-02	False
STKLD1	51.599	103.596	27.590	0.498	1.32E-02	False
CCL2	137.167	265.331	67.668	0.517	1.32E-02	False
CDKL1	4.730	13.892	5.071	0.340	1.33E-02	False
Non-Targeting_Control_044	0.860	4.929	2.533	0.174	1.35E-02	False
IGF1R	5.160	15.880	5.597	0.325	1.35E-02	False
TTBK2	0.000	4.920	2.531	0.169	1.36E-02	False
NUAK2	7.310	19.847	6.632	0.368	1.36E-02	False
Non-Targeting_Control_099	0.000	4.909	2.527	0.169	1.37E-02	False
FES	60.199	120.011	31.662	0.502	1.37E-02	False
SRC	181.886	346.236	87.702	0.525	1.38E-02	False
TBRG4	1.290	7.368	3.270	0.175	1.39E-02	False
LYN	3.440	11.721	4.488	0.293	1.39E-02	False
MPP1	8.170	21.706	7.112	0.376	1.40E-02	False
PRKAG3	0.000	4.869	2.514	0.170	1.41E-02	False
PRKACA	0.000	4.866	2.513	0.170	1.41E-02	False

PI4K2A	0.000	4.865	2.513	0.170	1.41E-02	False
OXSR1	501.369	939.707	234.620	0.534	1.44E-02	False
ITPKC	2.150	9.518	3.882	0.226	1.45E-02	False
CSNK1D	2.580	9.518	3.882	0.271	1.45E-02	False
PHKA1	3.870	11.607	4.457	0.333	1.47E-02	False
CSNK1G1	224.455	423.539	106.841	0.530	1.47E-02	False
LIMK2	64.929	126.378	33.241	0.514	1.47E-02	False
BLK	1.720	7.237	3.232	0.238	1.51E-02	False
STK32A	0.430	4.778	2.485	0.090	1.51E-02	False
Non-Targeting_Control_018	70.088	136.995	35.874	0.512	1.52E-02	False
CERKL	2.580	9.401	3.849	0.274	1.54E-02	False
ITPKC	127.277	241.806	61.842	0.526	1.55E-02	False
CKS2	0.860	4.740	2.473	0.181	1.56E-02	False
MAST4	10.320	25.109	7.984	0.411	1.58E-02	False
TWF2	0.000	4.714	2.465	0.175	1.59E-02	False
MAP3K11	2.580	9.314	3.825	0.277	1.61E-02	False
PIP4K2C	21.930	45.625	13.160	0.481	1.61E-02	False
PRKCE	149.637	280.991	71.546	0.533	1.61E-02	False
CDKL3	7.310	19.293	6.488	0.379	1.62E-02	False
CSNK1D	0.860	4.691	2.457	0.183	1.62E-02	False
PIK3R4	3.440	11.372	4.393	0.303	1.63E-02	False
MATK	2.580	9.270	3.812	0.278	1.65E-02	False
DGKH	60.629	117.197	30.964	0.517	1.65E-02	False
CDK15	83.418	159.087	41.350	0.524	1.67E-02	False
PEAK1	0.430	4.641	2.441	0.093	1.68E-02	False
AAK1	192.636	357.673	90.534	0.539	1.70E-02	False
RNASEL	0.860	4.616	2.434	0.186	1.72E-02	False
CIT	121.257	227.376	58.268	0.533	1.75E-02	False
SGK223	0.000	4.582	2.423	0.179	1.76E-02	False
STK39	2.580	9.132	3.774	0.283	1.77E-02	False
TRRAP	364.202	667.733	167.294	0.545	1.78E-02	False
TAF1L	193.496	356.941	90.352	0.542	1.80E-02	False
AKT1	0.000	4.542	2.410	0.180	1.82E-02	False
MAPKAPK3	30.529	61.233	17.058	0.499	1.83E-02	False
FPGT-TNNI3K	10.750	24.526	7.835	0.438	1.84E-02	False
SYK	6.880	16.976	5.885	0.405	1.84E-02	False
STK17A	0.860	4.511	2.400	0.191	1.86E-02	False
IPPK	7.740	18.842	6.371	0.411	1.86E-02	False
ADPGK	3.010	11.083	4.314	0.272	1.86E-02	False
ITPKB	3.440	11.068	4.310	0.311	1.88E-02	False
MARK3	0.000	4.499	2.396	0.182	1.88E-02	False
FASTKD3	14.620	31.827	9.690	0.459	1.88E-02	False
ADCK5	20.210	42.749	12.439	0.473	1.90E-02	False
MARK2	0.000	4.470	2.386	0.183	1.92E-02	False
RIPK3	35.689	69.652	19.155	0.512	1.96E-02	False
SBK3	0.000	4.435	2.375	0.184	1.98E-02	False
ITPKA	43.429	83.775	22.667	0.518	2.01E-02	False
MPP2	9.890	22.288	7.261	0.444	2.03E-02	False
GRK4	2.150	8.833	3.690	0.243	2.07E-02	False
TSSK3	0.000	4.371	2.354	0.186	2.08E-02	False
KSR1	35.689	69.054	19.006	0.517	2.09E-02	False
MAP4K5	110.938	202.619	52.136	0.548	2.09E-02	False
PIK3CG	12.900	27.639	8.628	0.467	2.11E-02	False
PRKX	39.129	76.082	20.755	0.514	2.11E-02	False
DYRK1B	13.760	29.452	9.089	0.467	2.12E-02	False
DGKD	4.730	12.752	4.766	0.371	2.12E-02	False
ACVR1B	0.860	4.333	2.342	0.198	2.14E-02	False
TSSK2	0.000	4.300	2.331	0.189	2.20E-02	False
ALPK3	0.000	4.296	2.330	0.189	2.21E-02	False
CDC42BPA	0.860	4.273	2.322	0.201	2.25E-02	False
CLK2	131.577	237.760	60.840	0.553	2.25E-02	False
FASTKD1	27.949	54.022	15.259	0.517	2.29E-02	False
EPHB4	10.320	23.657	7.612	0.436	2.30E-02	False
CAMK4	31.389	61.060	17.015	0.514	2.31E-02	False
PIM2	1.290	6.515	3.019	0.198	2.35E-02	False
ICK	22.790	44.968	12.995	0.507	2.36E-02	False
EPHA3	0.000	4.209	2.301	0.192	2.36E-02	False
Non-Targeting_Control_048	202.956	359.392	90.959	0.565	2.43E-02	False
PDXK	0.000	4.165	2.287	0.194	2.45E-02	False
ITK	25.369	49.896	14.228	0.508	2.48E-02	False
MYT1	7.310	17.917	6.131	0.408	2.49E-02	False
DDR2	40.849	76.033	20.743	0.537	2.51E-02	False
SBK3	178.876	315.955	80.204	0.566	2.52E-02	False

CDK3	18.490	37.491	11.118	0.493	2.52E-02	False
CARD11	34.829	65.467	18.113	0.532	2.54E-02	False
TTBK2	0.430	4.118	2.271	0.104	2.54E-02	False
GSG2	0.860	4.118	2.271	0.209	2.54E-02	False
CDK14	340.983	595.882	149.507	0.572	2.57E-02	False
AK1	20.640	40.867	11.967	0.505	2.58E-02	False
MAP4K3	2510.715	4342.554	1076.945	0.578	2.63E-02	False
STRADB	2.150	8.382	3.562	0.257	2.63E-02	False
MYO3A	157.807	277.798	70.755	0.568	2.64E-02	False
FES	1.720	6.316	2.960	0.272	2.67E-02	False
KDR	191.346	335.970	85.160	0.570	2.67E-02	False
TRIB1	10.320	23.075	7.463	0.447	2.67E-02	False
ACVR1C	0.000	4.010	2.235	0.200	2.77E-02	False
ANKK1	0.000	4.010	2.235	0.200	2.77E-02	False
IRAK4	5.590	13.930	5.081	0.401	2.80E-02	False
AK8	10.750	22.888	7.415	0.470	2.80E-02	False
CROT	24.079	47.017	13.508	0.512	2.91E-02	False
PAPSS1	15.050	31.456	9.596	0.478	2.92E-02	False
BCKDK	3.440	10.107	4.045	0.340	2.92E-02	False
LIMK2	12.470	26.206	8.264	0.476	2.94E-02	False
MUSK	3.010	10.079	4.038	0.299	2.96E-02	False
PIP4K2A	12.900	26.172	8.255	0.493	2.96E-02	False
PKN2	3.440	10.043	4.028	0.343	3.01E-02	False
MAP4K5	636.816	1085.179	270.630	0.587	3.03E-02	False
STK32A	1.290	6.107	2.897	0.211	3.04E-02	False
TWF2	1.290	6.098	2.894	0.212	3.06E-02	False
PRKG1	0.000	3.886	2.194	0.205	3.06E-02	False
AURKB	2.150	8.076	3.474	0.266	3.10E-02	False
RPS6KA3	0.860	3.862	2.186	0.223	3.12E-02	False
EPHA10	7.740	17.200	5.944	0.450	3.13E-02	False
TAOK1	14.620	29.365	9.066	0.498	3.15E-02	False
MORN1	3.870	9.906	3.990	0.391	3.21E-02	False
TK1	3.010	9.885	3.984	0.304	3.24E-02	False
TYRO3	0.860	3.802	2.165	0.226	3.28E-02	False
MYLK2	0.430	3.782	2.158	0.114	3.33E-02	False
BUB1B	9.460	20.442	6.786	0.463	3.38E-02	False
PDIK1L	372.802	628.164	157.498	0.593	3.39E-02	False
PLXNA3	3.440	9.752	3.947	0.353	3.45E-02	False
DGKK	24.939	45.753	13.192	0.545	3.49E-02	False
EIF2AK2	59.769	104.294	27.763	0.573	3.49E-02	False
NMRK1	191.346	324.238	82.255	0.590	3.49E-02	False
RPS6KA1	6.450	15.096	5.391	0.427	3.50E-02	False
ACVR1C	67.079	117.404	31.016	0.571	3.54E-02	False
MYO3A	16.340	32.186	9.781	0.508	3.54E-02	False
NEK8	28.379	52.310	14.832	0.543	3.56E-02	False
AK4	75.678	130.582	34.284	0.580	3.56E-02	False
SNX16	76.538	132.204	34.686	0.579	3.57E-02	False
PIK3R4	6.880	15.018	5.370	0.458	3.59E-02	False
SPHK1	0.000	3.681	2.124	0.214	3.62E-02	False
BRAF	25.369	47.162	13.544	0.538	3.62E-02	False
NME4	4.730	11.445	4.413	0.413	3.64E-02	False
PINK1	0.860	3.668	2.119	0.234	3.66E-02	False
MAP3K13	10.750	21.844	7.147	0.492	3.67E-02	False
DYRK3	17.630	33.631	10.145	0.524	3.70E-02	False
GALK2	3.870	9.559	3.893	0.405	3.77E-02	False
CHKA	3.870	9.535	3.887	0.406	3.81E-02	False
MAP2K4	0.000	3.614	2.101	0.217	3.82E-02	False
TXK	0.430	3.613	2.101	0.119	3.82E-02	False
MOK	5.590	13.087	4.856	0.427	3.83E-02	False
HUS1	5.590	13.078	4.854	0.427	3.85E-02	False
PHKB	64.499	110.921	29.407	0.581	3.88E-02	False
LRRK1	228.755	380.094	96.085	0.602	3.90E-02	False
FASTKD3	0.000	3.580	2.089	0.218	3.93E-02	False
LIMK2	6.450	14.713	5.289	0.438	3.99E-02	False
TEX14	0.000	3.560	2.082	0.219	4.00E-02	False
CKB	12.470	24.864	7.921	0.502	4.01E-02	False
CASK	4.300	11.200	4.346	0.384	4.03E-02	False
CDK13	14.190	28.135	8.754	0.504	4.07E-02	False
PDK2	46.009	80.717	21.907	0.570	4.08E-02	False
PAK6	0.000	3.533	2.073	0.221	4.08E-02	False
PHKB	0.430	3.532	2.073	0.122	4.09E-02	False
RIOK3	96.318	162.274	42.140	0.594	4.10E-02	False
PIP4K2C	3.870	9.378	3.843	0.413	4.10E-02	False

Non-Targeting_Control_004	0.000	3.526	2.071	0.221	4.11E-02	False
EGFR	2.150	7.540	3.320	0.285	4.12E-02	False
PTK6	0.430	3.522	2.069	0.122	4.12E-02	False
COASY	36.549	64.212	17.800	0.569	4.13E-02	False
CARD11	0.860	3.509	2.065	0.245	4.17E-02	False
NIM1K	0.000	3.497	2.060	0.222	4.21E-02	False
TAF1	15.480	29.602	9.126	0.523	4.23E-02	False
ERBB3	0.000	3.490	2.058	0.223	4.23E-02	False
CHEK2	3.010	9.270	3.813	0.325	4.31E-02	False
CDKL2	11.610	22.888	7.415	0.507	4.31E-02	False
RIPK2	158.237	261.375	66.688	0.605	4.33E-02	False
CIB4	4.300	10.984	4.287	0.391	4.41E-02	False
PIK3R1	33.109	58.656	16.415	0.564	4.44E-02	False
SRC	128.567	212.003	54.460	0.606	4.45E-02	False
BRDT	14.620	27.693	8.642	0.528	4.46E-02	False
C17orf75	0.000	3.425	2.036	0.226	4.46E-02	False
PRKACG	1.290	5.495	2.710	0.235	4.48E-02	False
TSSK6	0.860	3.419	2.033	0.252	4.49E-02	False
PIK3C2B	0.000	3.412	2.031	0.227	4.51E-02	False
ABL1	4.730	10.925	4.271	0.433	4.52E-02	False
DSTYK	3.010	9.163	3.783	0.328	4.53E-02	False
ACVRL1	6.450	14.338	5.190	0.450	4.54E-02	False
CAMKK2	39.129	68.032	18.752	0.575	4.59E-02	False
BRD4	3.440	9.131	3.773	0.377	4.60E-02	False
DDR2	1.720	5.444	2.694	0.316	4.63E-02	False
Non-Targeting_Control_014	44.289	75.759	20.674	0.585	4.72E-02	False
BAZ1B	7.740	15.898	5.602	0.487	4.73E-02	False
PXK	0.860	3.355	2.011	0.256	4.73E-02	False
STK32B	1.290	5.393	2.679	0.239	4.78E-02	False
TNK2	12.040	24.081	7.721	0.500	4.80E-02	False
MPP7	12.900	24.064	7.717	0.536	4.82E-02	False
Non-Targeting_Control_092	300.563	482.862	121.528	0.622	4.89E-02	False
TEC	0.000	3.304	1.993	0.232	4.94E-02	False
GUCY2D	6.450	14.092	5.124	0.458	4.94E-02	False
PLXNA4	4.300	10.699	4.209	0.402	4.96E-02	False
PRKAG2	176.296	284.915	72.518	0.619	4.97E-02	False
TSSK3	0.000	3.277	1.984	0.234	5.05E-02	False
RIPK2	1.290	5.309	2.652	0.243	5.05E-02	False
WEE1	1.290	5.305	2.651	0.243	5.06E-02	False
CAMKK1	0.000	3.263	1.979	0.235	5.11E-02	False
RPS6KB1	0.860	3.256	1.976	0.264	5.13E-02	False
Non-Targeting_Control_043	0.000	3.253	1.975	0.235	5.15E-02	False
SGK1	2.150	7.110	3.195	0.302	5.19E-02	False
TLK2	0.000	3.236	1.969	0.236	5.22E-02	False
BMPR1A	12.040	23.714	7.627	0.508	5.22E-02	False
ATR	12.470	23.713	7.627	0.526	5.22E-02	False
PIK3CB	327.653	520.652	130.883	0.629	5.24E-02	False
STKLD1	45.579	76.046	20.746	0.599	5.28E-02	False
LRPPRC	1.720	5.239	2.631	0.328	5.28E-02	False
CMPK2	16.340	30.048	9.240	0.544	5.31E-02	False
PRKACG	24.939	42.754	12.440	0.583	5.33E-02	False
PLK2	5.160	12.177	4.611	0.424	5.39E-02	False
NME5	13262.631	20812.812	5153.886	0.637	5.40E-02	False
AATK	0.430	3.187	1.952	0.135	5.44E-02	False
STK11	9.460	18.685	6.331	0.506	5.47E-02	False
MAPKAPK2	28.809	48.874	13.973	0.589	5.47E-02	False
LRPPRC	25.799	44.015	12.756	0.586	5.57E-02	False
ADCK2	4.730	10.413	4.130	0.454	5.59E-02	False
PRKCZ	2.580	6.971	3.154	0.370	5.59E-02	False
PRKCQ	2.580	6.962	3.151	0.371	5.62E-02	False
STK3	6.450	13.711	5.023	0.470	5.63E-02	False
BRD2	3.010	8.691	3.650	0.346	5.65E-02	False
STK25	15.480	28.121	8.751	0.550	5.67E-02	False
EGFR	0.000	3.129	1.931	0.242	5.71E-02	False
MAPK6	20.210	35.968	10.735	0.562	5.72E-02	False
PKMYT1	172.426	272.907	69.544	0.632	5.72E-02	False
SRPK2	0.000	3.109	1.924	0.243	5.81E-02	False
POMK	3.440	8.614	3.628	0.399	5.86E-02	False
MAP4K5	2.150	6.875	3.126	0.313	5.89E-02	False
MLKL	0.860	3.092	1.918	0.278	5.89E-02	False
PRKG2	10.750	19.995	6.670	0.538	5.92E-02	False
DGUOK	15.480	27.902	8.695	0.555	5.92E-02	False
MARK4	0.430	3.085	1.915	0.139	5.93E-02	False

MAPK7	1.290	5.060	2.575	0.255	5.93E-02	False
SLK	2.580	6.860	3.121	0.376	5.93E-02	False
MAPKAPK2	2.580	6.853	3.120	0.376	5.95E-02	False
SPHK1	353.882	551.152	138.434	0.642	5.97E-02	False
STK31	64.929	103.895	27.664	0.625	6.03E-02	False
AGK	3.870	8.551	3.610	0.453	6.03E-02	False
ARAF	20.210	35.591	10.640	0.568	6.08E-02	False
PAK6	0.000	3.055	1.904	0.247	6.08E-02	False
Non-Targeting_Control_009	61.059	99.033	26.457	0.617	6.11E-02	False
CSNK1A1L	1.720	5.007	2.558	0.344	6.13E-02	False
TLK1	1.290	4.989	2.552	0.259	6.20E-02	False
TBK1	0.000	3.028	1.895	0.248	6.22E-02	False
MAP2K7	0.000	3.014	1.890	0.249	6.29E-02	False
CHKB	10.750	19.733	6.602	0.545	6.33E-02	False
Non-Targeting_Control_059	75.678	119.774	31.604	0.632	6.41E-02	False
PRKAB2	9.890	18.081	6.173	0.547	6.46E-02	False
GTF2H1	8.170	16.470	5.752	0.496	6.51E-02	False
DGKB	3.010	8.374	3.560	0.359	6.55E-02	False
FGGY	15.480	27.338	8.552	0.566	6.62E-02	False
CDK19	68.369	108.262	28.748	0.632	6.71E-02	False
MARK4	45.579	73.175	20.032	0.623	6.73E-02	False
CAMK1G	954.579	1453.901	361.903	0.657	6.77E-02	False
FYN	34.829	56.305	15.829	0.619	6.79E-02	False
BRD4	0.000	2.921	1.856	0.255	6.81E-02	False
C17orf75	1.720	4.838	2.504	0.356	6.84E-02	False
JAK2	9.030	17.805	6.102	0.507	6.96E-02	False
MAP4K1	6.880	13.081	4.855	0.526	6.98E-02	False
PTK2B	13.760	23.991	7.698	0.574	6.98E-02	False
PHKG1	0.000	2.884	1.842	0.257	7.03E-02	False
DGKG	1.290	4.791	2.489	0.269	7.05E-02	False
FLT4	3.010	8.213	3.514	0.366	7.06E-02	False
SGK2	109.648	169.294	43.880	0.648	7.07E-02	False
TGFBR2	7.740	14.599	5.259	0.530	7.12E-02	False
CDK6	0.000	2.867	1.836	0.259	7.13E-02	False
DGKG	1.290	4.772	2.483	0.270	7.14E-02	False
MELK	0.430	2.857	1.832	0.151	7.19E-02	False
RPS6KB2	8.170	16.131	5.663	0.506	7.19E-02	False
NEK1	30.099	49.633	14.162	0.606	7.26E-02	False
SH3BP5L	7.740	14.537	5.243	0.532	7.26E-02	False
STK33	18.490	31.401	9.582	0.589	7.31E-02	False
CDKL2	12.040	22.159	7.228	0.543	7.46E-02	False
JAK1	9.030	17.551	6.035	0.514	7.46E-02	False
PRKAR2B	0.430	2.813	1.816	0.153	7.46E-02	False
DDR1	509.539	766.673	191.787	0.665	7.47E-02	False
TGFBR1	53.319	83.920	22.703	0.635	7.48E-02	False
MYT1	64.069	100.396	26.795	0.638	7.49E-02	False
DYRK2	18.920	31.250	9.544	0.605	7.51E-02	False
PANK3	3.870	8.077	3.475	0.479	7.52E-02	False
ALPK2	4.730	9.687	3.929	0.488	7.55E-02	False
PGK2	0.000	2.794	1.809	0.264	7.59E-02	False
PRPS1L1	0.000	2.794	1.809	0.264	7.59E-02	False
MERTK	529.318	794.702	198.725	0.666	7.59E-02	False
PAPSS2	8.170	15.946	5.615	0.512	7.59E-02	False
STK10	2.580	6.389	2.982	0.404	7.64E-02	False
TPK1	4.730	9.654	3.920	0.490	7.65E-02	False
MAP3K19	4.730	9.647	3.918	0.490	7.67E-02	False
NRK	71.808	110.390	29.275	0.651	7.70E-02	False
MAP2K6	0.000	2.776	1.802	0.265	7.70E-02	False
ADCK4	0.000	2.776	1.802	0.265	7.70E-02	False
MAP2K3	5.590	11.217	4.350	0.498	7.72E-02	False
PRKACA	16.770	28.046	8.732	0.598	7.76E-02	False
OXSR1	184.896	278.131	70.838	0.665	7.83E-02	False
PIP4K2C	0.430	2.755	1.795	0.156	7.84E-02	False
NME6	0.430	2.748	1.792	0.156	7.89E-02	False
AK1	247.245	371.335	93.916	0.666	7.89E-02	False
FGR	17.630	29.437	9.085	0.599	7.92E-02	False
CALM3	2.150	6.322	2.961	0.340	7.92E-02	False
DGKI	10.320	18.836	6.370	0.548	7.97E-02	False
SHPK	1.290	4.593	2.426	0.281	8.01E-02	False
PI4K2B	5.160	11.117	4.323	0.464	8.02E-02	False
MYO3A	34.399	54.640	15.413	0.630	8.10E-02	False
PNKP	12.040	21.780	7.131	0.553	8.13E-02	False
TAF1L	78.258	119.603	31.561	0.654	8.20E-02	False

TTBK1	6.020	12.605	4.726	0.478	8.21E-02	False
LRRK2	39.559	61.910	17.227	0.639	8.22E-02	False
ETNK2	21.070	35.163	10.532	0.599	8.26E-02	False
PACSIN1	4.300	9.467	3.868	0.454	8.27E-02	False
MELK	6.450	12.537	4.708	0.514	8.41E-02	False
STK17B	105.348	158.825	41.285	0.663	8.42E-02	False
CDK5	5.160	10.951	4.278	0.471	8.53E-02	False
FER	0.000	2.652	1.756	0.274	8.57E-02	False
BMPR2	39.129	61.412	17.103	0.637	8.61E-02	False
PGM2L1	1.290	4.481	2.390	0.288	8.62E-02	False
PIP4K2B	17.200	28.967	8.966	0.594	8.62E-02	False
PPIP5K1	37.839	58.427	16.358	0.648	8.65E-02	False
BAZ1B	161.676	239.876	61.364	0.674	8.71E-02	False
Non-Targeting_Control_072	353.022	519.833	130.680	0.679	8.80E-02	False
PIK3CG	11.180	19.923	6.652	0.561	8.84E-02	False
ULK2	133.297	198.118	51.021	0.673	8.93E-02	False
GUCY2D	81.698	122.233	32.213	0.668	8.97E-02	False
TRIM24	20.210	33.152	10.025	0.610	8.98E-02	False
AKT3	2.580	6.079	2.889	0.424	9.03E-02	False
UCKL1	0.430	2.581	1.729	0.167	9.11E-02	False
STK26	455.790	663.498	166.245	0.687	9.25E-02	False
MKNK1	5.590	10.720	4.215	0.521	9.29E-02	False
DTYMK	0.860	2.557	1.720	0.336	9.30E-02	False
MAP3K5	36.979	56.037	15.762	0.660	9.49E-02	False
ACVR1B	209.835	305.611	77.643	0.687	9.54E-02	False
MAST1	4.730	9.107	3.767	0.519	9.59E-02	False
HK3	28.379	44.251	12.815	0.641	9.71E-02	False
NTPCR	0.430	2.503	1.699	0.172	9.74E-02	False
PIK3R3	1.720	4.289	2.327	0.401	9.75E-02	False
LRRK2	1.720	4.283	2.325	0.402	9.80E-02	False
DAPK2	327.653	473.689	119.257	0.692	9.80E-02	False
Non-Targeting_Control_015	505.239	728.741	182.396	0.693	9.84E-02	False
MAP3K6	0.000	2.489	1.694	0.287	9.86E-02	False
GLYCTK	0.000	2.489	1.694	0.287	9.86E-02	False
PKLR	7.310	13.558	4.982	0.539	9.87E-02	False
NRBP2	0.000	2.487	1.693	0.287	9.88E-02	False
LATS1	15.480	25.269	8.024	0.613	9.93E-02	False
GSAP	8.600	15.016	5.370	0.573	9.94E-02	False
ROCK1	4.300	8.997	3.736	0.478	1.00E-01	False
DGKK	491.479	706.046	176.778	0.696	1.01E-01	False
NME1	3.440	7.445	3.293	0.462	1.01E-01	False
RPS6KA5	254.124	366.716	92.772	0.693	1.02E-01	False
PRKAR2B	0.430	2.453	1.680	0.175	1.02E-01	False
CDKL3	576.617	826.191	206.520	0.698	1.02E-01	False
SBK2	1280.082	1828.746	454.691	0.700	1.03E-01	False
PRKAG1	55.899	82.287	22.297	0.679	1.03E-01	False
PEAK1	4.730	8.916	3.713	0.531	1.04E-01	False
SH3BP5L	3.440	7.373	3.272	0.467	1.04E-01	False
FN3K	9.030	16.307	5.710	0.554	1.05E-01	False
MYO3B	3.440	7.365	3.269	0.467	1.05E-01	False
TEX14	2.580	5.790	2.801	0.446	1.05E-01	False
ADCK1	134.587	194.171	50.043	0.693	1.06E-01	False
BMPR1B	13.330	22.029	7.195	0.605	1.07E-01	False
MAP3K10	78.258	114.414	30.274	0.684	1.07E-01	False
CERK	15.910	24.814	7.908	0.641	1.08E-01	False
HIPK2	18.490	29.090	8.997	0.636	1.09E-01	False
CHEK1	0.000	2.376	1.650	0.296	1.09E-01	False
MAP3K2	0.860	2.369	1.647	0.363	1.09E-01	False
TAOK2	18.490	29.026	8.980	0.637	1.10E-01	False
PFKFB4	66.649	96.859	25.917	0.688	1.10E-01	False
FLT3	23.649	36.073	10.761	0.656	1.11E-01	False
TXK	7.310	13.164	4.877	0.555	1.11E-01	False
PI4KB	10.750	17.476	6.016	0.615	1.12E-01	False
TAF1	0.000	2.325	1.630	0.301	1.14E-01	False
SNRK	0.000	2.298	1.619	0.303	1.16E-01	False
PRKAR1B	443.750	621.024	155.731	0.715	1.18E-01	False
PRKCH	17.200	27.175	8.510	0.633	1.18E-01	False
ALPK3	0.860	2.274	1.610	0.378	1.19E-01	False
Non-Targeting_Control_042	1.290	3.981	2.225	0.324	1.19E-01	False
CDK2	0.000	2.268	1.607	0.306	1.20E-01	False
MAST1	0.000	2.257	1.603	0.307	1.21E-01	False
DYRK1A	0.000	2.247	1.599	0.308	1.22E-01	False
PLK4	0.000	2.247	1.599	0.308	1.22E-01	False

DGKI	14.190	22.798	7.392	0.622	1.22E-01	False
CDK10	147.057	207.439	53.330	0.709	1.22E-01	False
JAK1	9.460	15.658	5.539	0.604	1.24E-01	False
PIK3CA	1.290	3.903	2.199	0.331	1.25E-01	False
ALPK2	0.860	2.208	1.583	0.390	1.26E-01	False
RIPK3	202.956	282.026	71.802	0.720	1.26E-01	False
FASTKD5	563.288	778.377	194.684	0.724	1.27E-01	False
EPHA10	6.880	11.305	4.374	0.609	1.27E-01	False
SIK2	137.597	192.169	49.547	0.716	1.27E-01	False
AURKC	65.789	93.034	24.967	0.707	1.27E-01	False
BTK	362.912	500.991	126.016	0.724	1.28E-01	False
EEF2K	79.118	112.072	29.693	0.706	1.29E-01	False
PNKP	64.929	91.347	24.548	0.711	1.30E-01	False
UHMK1	5.160	9.796	3.959	0.527	1.31E-01	False
PRPS2	582.637	800.061	200.052	0.728	1.31E-01	False
HIPK3	5.160	9.777	3.954	0.528	1.32E-01	False
CDC42BPG	3.010	6.864	3.123	0.439	1.32E-01	False
PLK2	20.210	30.699	9.404	0.658	1.32E-01	False
FPGT-TNNI3K	286.804	394.699	99.701	0.727	1.32E-01	False
DOLK	17.630	26.535	8.347	0.664	1.32E-01	False
TTBK1	112.228	156.644	40.744	0.716	1.33E-01	False
STK35	4.300	8.301	3.539	0.518	1.33E-01	False
MAPK14	0.000	2.144	1.557	0.318	1.33E-01	False
GK	0.000	2.144	1.557	0.318	1.33E-01	False
KIT	0.000	2.144	1.557	0.318	1.33E-01	False
PI4KA	33.969	48.290	13.827	0.703	1.36E-01	False
CKMT1A	0.000	2.120	1.548	0.321	1.36E-01	False
STK40	1.290	3.775	2.156	0.342	1.36E-01	False
NEK4	37.409	53.718	15.183	0.696	1.37E-01	False
RPS6KC1	11.180	18.075	6.172	0.619	1.37E-01	False
STK39	3.440	6.782	3.099	0.507	1.37E-01	False
DGKB	0.000	2.113	1.545	0.321	1.37E-01	False
Non-Targeting_Control_094	297.984	406.061	102.514	0.734	1.39E-01	False
EPHA4	110.938	152.574	39.736	0.727	1.39E-01	False
PPIP5K2	103.628	143.044	37.374	0.724	1.39E-01	False
Non-Targeting_Control_023	14.190	22.130	7.221	0.641	1.39E-01	False
SHPK	53.319	74.977	20.480	0.711	1.42E-01	False
ARAF	0.000	2.073	1.528	0.325	1.42E-01	False
FASTKD5	2.580	5.220	2.625	0.494	1.43E-01	False
STYK1	2.150	5.198	2.618	0.414	1.45E-01	False
JAK1	0.000	2.052	1.520	0.328	1.45E-01	False
GK5	71.808	98.854	26.412	0.726	1.46E-01	False
Non-Targeting_Control_001	21.930	31.416	9.586	0.698	1.46E-01	False
MAK	75.248	103.994	27.688	0.724	1.47E-01	False
PRKCA	27.949	39.423	11.604	0.709	1.48E-01	False
SNX16	0.430	2.022	1.507	0.213	1.49E-01	False
CAMKK2	10.750	16.347	5.720	0.658	1.49E-01	False
PHKG2	11.180	17.710	6.077	0.631	1.49E-01	False
RP2	0.000	2.019	1.506	0.331	1.49E-01	False
DGKD	0.000	2.019	1.506	0.331	1.49E-01	False
RIOK3	0.000	2.015	1.505	0.332	1.50E-01	False
DYRK2	10.750	16.316	5.712	0.659	1.50E-01	False
TGFBR3	0.430	2.005	1.500	0.214	1.51E-01	False
PTK6	1.290	3.614	2.101	0.357	1.51E-01	False
INSRR	0.000	2.002	1.499	0.333	1.51E-01	False
FGGY	116.957	158.094	41.104	0.740	1.52E-01	False
WNK4	0.000	1.991	1.495	0.334	1.53E-01	False
KHK	0.860	1.991	1.495	0.432	1.53E-01	False
SLK	11.180	17.492	6.020	0.639	1.57E-01	False
MARK4	0.000	1.955	1.480	0.338	1.58E-01	False
ACVR2B	1.720	3.546	2.078	0.485	1.58E-01	False
MAGI3	1.720	3.543	2.076	0.485	1.58E-01	False
Non-Targeting_Control_076	6.880	10.642	4.193	0.646	1.59E-01	False
RPS6KL1	1.290	3.539	2.075	0.364	1.59E-01	False
GUCY2F	20.640	29.439	9.085	0.701	1.60E-01	False
TAF9	383.552	509.191	128.046	0.753	1.61E-01	False
CDK19	5.160	9.215	3.797	0.560	1.61E-01	False
FN3KRP	0.430	1.928	1.468	0.223	1.62E-01	False
STK38L	5.590	9.210	3.796	0.607	1.62E-01	False
PRKAG2	6.450	10.576	4.175	0.610	1.62E-01	False
KHK	0.000	1.925	1.467	0.342	1.62E-01	False
HIPK2	14.190	21.365	7.024	0.664	1.62E-01	False
GK5	104.488	140.303	36.694	0.745	1.63E-01	False

PRKRA	886.641	1169.147	291.416	0.758	1.64E-01	False
BRSK2	72.238	97.850	26.163	0.738	1.65E-01	False
CKM	19.350	27.908	8.697	0.693	1.65E-01	False
ACVR1B	0.860	1.901	1.457	0.452	1.66E-01	False
PRKACA	12.040	18.567	6.300	0.648	1.66E-01	False
CAMKV	38.699	52.916	14.983	0.731	1.67E-01	False
ACVR1C	2.580	4.929	2.533	0.523	1.67E-01	False
ULK3	2.150	4.916	2.529	0.437	1.68E-01	False
Non-Targeting_Control_012	21.070	30.414	9.332	0.693	1.69E-01	False
BMPR1B	4.730	7.709	3.369	0.614	1.69E-01	False
SIK3	11.610	17.156	5.932	0.677	1.69E-01	False
CSNK1G1	21.070	30.361	9.319	0.694	1.70E-01	False
Non-Targeting_Control_089	132.867	175.712	45.470	0.756	1.70E-01	False
MAP3K12	96.748	128.600	33.793	0.752	1.70E-01	False
ETNK2	0.430	1.868	1.443	0.230	1.71E-01	False
STK25	4.730	7.682	3.361	0.616	1.71E-01	False
CIB1	0.000	1.864	1.441	0.349	1.71E-01	False
EPHA4	1.720	3.415	2.032	0.504	1.72E-01	False
ADK	2.150	4.868	2.514	0.442	1.72E-01	False
TAOK2	3.870	6.272	2.947	0.617	1.73E-01	False
MAST3	6.020	10.367	4.117	0.581	1.74E-01	False
PKMYT1	0.000	1.847	1.434	0.351	1.74E-01	False
LYN	21.500	30.209	9.281	0.712	1.74E-01	False
IRAK3	116.957	154.113	40.117	0.759	1.74E-01	False
ITPK1	0.000	1.844	1.433	0.352	1.74E-01	False
PIK3C3	41.279	56.328	15.835	0.733	1.75E-01	False
PDGFRB	30.959	41.943	12.237	0.738	1.75E-01	False
WNK4	0.000	1.834	1.428	0.353	1.76E-01	False
GRK7	3.440	6.224	2.932	0.553	1.77E-01	False
NTRK2	7.310	11.652	4.469	0.627	1.77E-01	False
LIMK1	0.000	1.820	1.422	0.355	1.78E-01	False
TAB1	1.290	3.358	2.012	0.384	1.79E-01	False
TRPM6	179.736	235.037	60.166	0.765	1.79E-01	False
CDK5R1	52.459	70.304	19.317	0.746	1.79E-01	False
MYT1	12.900	18.219	6.210	0.708	1.79E-01	False
MAP3K8	325.073	423.659	106.871	0.767	1.80E-01	False
NEK7	4.300	7.546	3.322	0.570	1.81E-01	False
CDK13	0.000	1.803	1.415	0.357	1.81E-01	False
CDK12	0.000	1.803	1.415	0.357	1.81E-01	False
PGK2	173.716	226.498	58.051	0.767	1.82E-01	False
TRIM27	18.920	25.988	8.208	0.728	1.82E-01	False
C8orf44-SGK3	45.149	60.971	16.993	0.741	1.83E-01	False
IPMK	1.720	3.307	1.994	0.520	1.85E-01	False
DLG3	3044.764	3922.356	972.932	0.776	1.85E-01	False
TRIM33	2.150	4.731	2.470	0.454	1.85E-01	False
STK19	0.000	1.766	1.399	0.361	1.87E-01	False
CDK18	0.000	1.760	1.396	0.362	1.88E-01	False
ICK	0.000	1.760	1.396	0.362	1.88E-01	False
Non-Targeting_Control_053	0.000	1.756	1.395	0.363	1.89E-01	False
PTK7	33.539	45.100	13.028	0.744	1.89E-01	False
MAP3K6	3.870	6.072	2.886	0.637	1.89E-01	False
CSNK1E	0.000	1.749	1.392	0.364	1.90E-01	False
PSKH2	0.000	1.749	1.392	0.364	1.90E-01	False
MPP2	657.456	844.701	211.102	0.778	1.90E-01	False
TKFC	1.720	3.260	1.978	0.528	1.90E-01	False
Non-Targeting_Control_085	0.430	1.742	1.389	0.247	1.91E-01	False
CLK1	218.005	281.889	71.768	0.773	1.91E-01	False
PKD2	21.500	29.455	9.089	0.730	1.94E-01	False
MAPK14	6.450	10.023	4.022	0.644	1.94E-01	False
PIK3R1	48.159	63.908	17.724	0.754	1.96E-01	False
CDK13	24.079	33.207	10.039	0.725	1.97E-01	False
ERBB4	1431.009	1824.746	453.701	0.784	1.97E-01	False
STK24	769.253	980.858	244.807	0.784	1.98E-01	False
BLK	15.480	21.621	7.090	0.716	1.98E-01	False
AK2	405.481	517.773	130.170	0.783	1.98E-01	False
RP2	8.600	12.573	4.718	0.684	1.98E-01	False
PRPS1	12.900	17.749	6.087	0.727	1.98E-01	False
BRAF	40.419	53.477	15.123	0.756	1.99E-01	False
Non-Targeting_Control_022	59.339	77.619	21.137	0.764	2.00E-01	False
CAMK4	0.000	1.676	1.360	0.374	2.03E-01	False
PLK1	0.000	1.676	1.360	0.374	2.03E-01	False
FGFRL1	0.000	1.676	1.360	0.374	2.03E-01	False
NRBP2	0.000	1.676	1.360	0.374	2.03E-01	False

RPS6KA2	8.170	12.473	4.691	0.655	2.04E-01	False
ALPK1	2.150	4.539	2.408	0.474	2.05E-01	False
STK26	655.306	829.044	207.226	0.790	2.06E-01	False
LYN	74.388	95.917	25.683	0.776	2.07E-01	False
PRPF4B	0.000	1.649	1.347	0.377	2.08E-01	False
STK36	6.450	9.786	3.956	0.659	2.09E-01	False
MPP6	3.440	5.845	2.818	0.589	2.09E-01	False
CDK3	6.880	9.777	3.954	0.704	2.10E-01	False
PAK6	5.160	8.446	3.580	0.611	2.12E-01	False
DGKE	239.505	302.465	76.864	0.792	2.12E-01	False
EPHB4	0.000	1.622	1.335	0.381	2.13E-01	False
JAK2	3.010	5.806	2.805	0.518	2.13E-01	False
PLK2	156.517	197.714	50.921	0.792	2.16E-01	False
Non-Targeting_Control_077	85.138	108.751	28.869	0.783	2.17E-01	False
FASTKD1	2.150	4.425	2.372	0.486	2.18E-01	False
MAP2K5	1.720	3.052	1.903	0.564	2.18E-01	False
PCK1	73.098	93.696	25.132	0.780	2.18E-01	False
PRKCA	316.043	397.021	100.276	0.796	2.18E-01	False
NME9	5.590	8.369	3.558	0.668	2.18E-01	False
ROR1	0.430	1.595	1.323	0.270	2.18E-01	False
TGFBR1	0.000	1.595	1.323	0.385	2.18E-01	False
EPHA6	5.160	8.361	3.556	0.617	2.19E-01	False
UCK1	2.580	4.401	2.364	0.586	2.20E-01	False
GSK3B	30.529	39.815	11.702	0.767	2.20E-01	False
FGFR4	24.939	32.270	9.802	0.773	2.22E-01	False
BCR	139.317	175.398	45.392	0.794	2.22E-01	False
PDK1	4.300	7.016	3.167	0.613	2.23E-01	False
PFKFB1	250.685	313.247	79.533	0.800	2.23E-01	False
CMPK1	3.440	5.703	2.774	0.603	2.23E-01	False
ATMIN	25.799	33.447	10.099	0.771	2.24E-01	False
PKN2	0.000	1.561	1.308	0.390	2.25E-01	False
TPD52L3	12.470	17.096	5.916	0.729	2.27E-01	False
MAP3K3	61.059	77.836	21.191	0.784	2.29E-01	False
FGFR1	0.000	1.541	1.299	0.394	2.29E-01	False
PAPSS2	0.000	1.541	1.299	0.394	2.29E-01	False
RP2	651.436	804.504	201.151	0.810	2.33E-01	False
RIOK2	316.043	391.646	98.945	0.807	2.33E-01	False
BCKDK	18.060	24.439	7.813	0.739	2.33E-01	False
BTK	440.740	543.524	136.545	0.811	2.35E-01	False
PIK3C2A	759.363	935.711	233.631	0.812	2.35E-01	False
PANK3	0.430	1.514	1.286	0.284	2.35E-01	False
DLG3	0.000	1.514	1.286	0.398	2.35E-01	False
TAF1	0.000	1.514	1.286	0.398	2.35E-01	False
PMVK	0.000	1.514	1.286	0.398	2.35E-01	False
TRIB3	118.677	147.408	38.455	0.805	2.36E-01	False
SMG1	63.639	79.780	21.674	0.798	2.36E-01	False
EPHA2	5.160	8.134	3.491	0.634	2.37E-01	False
TBRG4	0.430	1.507	1.283	0.285	2.37E-01	False
TJP2	23.649	30.510	9.357	0.775	2.37E-01	False
SGK2	0.430	1.504	1.282	0.286	2.37E-01	False
MOK	0.000	1.504	1.282	0.399	2.37E-01	False
TRIB3	0.000	1.500	1.280	0.400	2.38E-01	False
STRADA	460.090	566.206	142.161	0.813	2.39E-01	False
NMRK2	460.090	566.202	142.160	0.813	2.39E-01	False
POMK	44.289	56.258	15.817	0.787	2.39E-01	False
CSNK1E	0.000	1.497	1.278	0.400	2.39E-01	False
CHKA	1.290	2.907	1.851	0.444	2.39E-01	False
PTK6	0.000	1.494	1.277	0.401	2.40E-01	False
UCK1	0.000	1.494	1.277	0.401	2.40E-01	False
NEK4	28.809	36.521	10.874	0.789	2.41E-01	False
PDK1	1.290	2.890	1.845	0.446	2.41E-01	False
PFKFB2	48.159	60.983	16.996	0.790	2.42E-01	False
CDK7	4.300	6.754	3.090	0.637	2.46E-01	False
TTBK1	1.290	2.857	1.832	0.452	2.47E-01	False
SH3BP4	25.369	32.508	9.862	0.780	2.51E-01	False
STK17B	12.040	16.614	5.790	0.725	2.51E-01	False
SHPK	7.740	10.446	4.139	0.741	2.52E-01	False
STK32B	888.791	1078.813	269.055	0.824	2.52E-01	False
CALM1	1.290	2.823	1.820	0.457	2.52E-01	False
PLK3	9.890	12.910	4.809	0.766	2.52E-01	False
ACVR2A	75719.632	91699.837	22700.795	0.826	2.53E-01	False
AK2	9.460	12.873	4.799	0.735	2.54E-01	False
PRKAR2A	14.620	18.981	6.408	0.770	2.55E-01	False

LATS1	75.248	92.870	24.927	0.810	2.55E-01	False
PRKD2	14.620	18.970	6.405	0.771	2.56E-01	False
UCK2	1409.079	1704.034	423.821	0.827	2.56E-01	False
HIPK1	46.439	57.667	16.169	0.805	2.58E-01	False
NEK6	1.290	2.776	1.802	0.465	2.60E-01	False
HIPK3	6.880	9.094	3.763	0.757	2.60E-01	False
ERN2	79.978	97.269	26.019	0.822	2.61E-01	False
PIM2	9.030	12.761	4.769	0.708	2.61E-01	False
PIK3R4	0.000	1.397	1.231	0.417	2.62E-01	False
NEK10	0.000	1.397	1.231	0.417	2.62E-01	False
SRMS	87.288	106.741	28.370	0.818	2.62E-01	False
EEF2K	75.678	92.304	24.786	0.820	2.62E-01	False
ABCC1	50.309	62.231	17.307	0.808	2.62E-01	False
PTK2B	113.088	137.760	36.064	0.821	2.64E-01	False
DCLK1	7.310	10.272	4.091	0.712	2.64E-01	False
ACVR1	103.628	125.533	33.032	0.826	2.66E-01	False
PFKFB4	7.310	10.236	4.081	0.714	2.67E-01	False
EPHB2	6.020	8.993	3.735	0.669	2.68E-01	False
PAPSS2	7.310	10.215	4.075	0.716	2.69E-01	False
SGK494	0.000	1.366	1.217	0.423	2.69E-01	False
PRKAR2B	18.920	23.477	7.566	0.806	2.70E-01	False
IRAK4	28.809	35.467	10.609	0.812	2.70E-01	False
TEX14	12.900	16.244	5.693	0.794	2.71E-01	False
PRKCD	8.170	11.400	4.400	0.717	2.71E-01	False
FASTKD2	30.099	37.764	11.187	0.797	2.73E-01	False
PKN1	18.060	23.361	7.537	0.773	2.75E-01	False
FLT3	0.000	1.343	1.205	0.427	2.75E-01	False
TBRG4	63.209	77.000	20.983	0.821	2.75E-01	False
PRKG2	0.000	1.336	1.202	0.428	2.77E-01	False
RP2	0.000	1.336	1.202	0.428	2.77E-01	False
SCYL3	0.000	1.336	1.202	0.428	2.77E-01	False
MUSK	0.860	1.336	1.202	0.644	2.77E-01	False
BCKDK	9.030	12.505	4.700	0.722	2.78E-01	False
RIPK4	4026.862	4771.460	1183.114	0.844	2.80E-01	False
ETNK1	423.111	503.066	126.530	0.841	2.81E-01	False
MAK	1125.716	1333.645	332.136	0.844	2.81E-01	False
GALK2	0.000	1.316	1.192	0.432	2.82E-01	False
CSF1R	119.967	142.794	37.312	0.840	2.82E-01	False
RPS6KB1	2.580	3.917	2.204	0.659	2.82E-01	False
CDK4	4.300	6.387	2.981	0.673	2.83E-01	False
ITK	32.679	39.695	11.672	0.823	2.86E-01	False
JAK3	27.089	33.768	10.180	0.802	2.86E-01	False
PRPS2	36.119	44.380	12.848	0.814	2.87E-01	False
FGR	7.740	9.970	4.008	0.776	2.88E-01	False
TSSK2	19.780	24.233	7.760	0.816	2.88E-01	False
CRKL	0.000	1.289	1.179	0.437	2.89E-01	False
GUCY2C	0.000	1.289	1.179	0.437	2.89E-01	False
SRC	0.000	1.289	1.179	0.437	2.89E-01	False
PKN3	5.590	7.530	3.317	0.742	2.90E-01	False
YES1	36.979	44.220	12.807	0.836	2.91E-01	False
CDK4	4.300	6.312	2.958	0.681	2.91E-01	False
PANK1	15.480	19.415	6.520	0.797	2.92E-01	False
MPP3	119.537	141.623	37.021	0.844	2.92E-01	False
TRIO	18.060	22.949	7.431	0.787	2.93E-01	False
KIAA1804	0.000	1.275	1.172	0.440	2.93E-01	False
CDKL4	0.000	1.275	1.172	0.440	2.93E-01	False
LRRK2	119.107	141.505	36.992	0.842	2.94E-01	False
PDK4	15684.769	18387.545	4553.552	0.853	2.94E-01	False
MORN1	47.729	57.006	16.004	0.837	2.94E-01	False
PIP5KL1	12.470	15.831	5.584	0.788	2.94E-01	False
RAF1	7908.388	9268.815	2296.361	0.853	2.94E-01	False
STK24	101.048	120.177	31.703	0.841	2.96E-01	False
EPHA8	0.000	1.262	1.166	0.442	2.97E-01	False
TRRAP	0.000	1.262	1.166	0.442	2.97E-01	False
CDC7	0.000	1.262	1.166	0.442	2.97E-01	False
RIOK1	0.000	1.262	1.166	0.442	2.97E-01	False
MAPK15	0.000	1.262	1.166	0.442	2.97E-01	False
DCAKD	131.577	155.138	40.371	0.848	2.97E-01	False
MAPK14	0.000	1.255	1.162	0.443	2.99E-01	False
PIK3CG	0.000	1.255	1.162	0.443	2.99E-01	False
DAPK3	0.430	1.251	1.161	0.344	2.99E-01	False
ITPKC	0.430	1.251	1.161	0.344	2.99E-01	False
BRDT	0.000	1.251	1.161	0.444	2.99E-01	False

PRKDC	0.000	1.251	1.161	0.444	2.99E-01	False
FASTK	0.000	1.251	1.161	0.444	2.99E-01	False
NEK8	48.159	57.899	16.227	0.832	3.00E-01	False
CDK15	1.290	2.550	1.717	0.506	3.00E-01	False
PNCK	5.590	7.433	3.289	0.752	3.00E-01	False
CSF1R	0.000	1.248	1.159	0.445	3.00E-01	False
STK33	6.020	8.624	3.631	0.698	3.00E-01	False
PFKFB1	39.559	47.333	13.587	0.836	3.01E-01	False
CSNK1G2	144.047	169.849	44.017	0.848	3.01E-01	False
MARK2	0.000	1.245	1.157	0.446	3.01E-01	False
MAPK1	0.000	1.245	1.157	0.446	3.01E-01	False
LAMTOR3	0.000	1.245	1.157	0.446	3.01E-01	False
GLYCTK	0.000	1.245	1.157	0.446	3.01E-01	False
TRIM28	0.860	1.245	1.157	0.691	3.01E-01	False
AK5	0.860	1.245	1.157	0.691	3.01E-01	False
PHKG2	784.303	915.089	228.526	0.857	3.03E-01	False
RPS6KA4	76.538	90.374	24.307	0.847	3.03E-01	False
SH3BP4	2.150	3.775	2.156	0.570	3.03E-01	False
MAP2K6	16.770	20.356	6.764	0.824	3.04E-01	False
ULK3	2.150	3.768	2.154	0.571	3.04E-01	False
PDPK1	5.160	7.388	3.276	0.698	3.05E-01	False
ALK	15027.313	17476.611	4328.065	0.860	3.05E-01	False
AGK	1.290	2.523	1.707	0.511	3.05E-01	False
Non-Targeting_Control_090	4.730	6.187	2.921	0.764	3.05E-01	False
TYK2	21.070	26.171	8.255	0.805	3.05E-01	False
TRIO	20.640	24.985	7.952	0.826	3.06E-01	False
ADCK5	27.519	33.112	10.014	0.831	3.07E-01	False
OSR1	11.180	14.437	5.216	0.774	3.07E-01	False
CDK14	56.759	66.776	18.439	0.850	3.08E-01	False
ADK	17.200	21.414	7.036	0.803	3.09E-01	False
IGFN1	52.029	62.058	17.264	0.838	3.10E-01	False
ATR	1.290	2.496	1.696	0.517	3.10E-01	False
MPP4	3.010	4.943	2.538	0.609	3.10E-01	False
RFK	13.760	16.719	5.818	0.823	3.11E-01	False
FN3KRP	1.290	2.489	1.694	0.518	3.12E-01	False
CDC42BPA	88.578	103.604	27.592	0.855	3.12E-01	False
PIK3C2B	116.097	135.972	35.620	0.854	3.12E-01	False
RIPK4	30.099	36.431	10.851	0.826	3.13E-01	False
TEC	155.657	180.963	46.771	0.860	3.13E-01	False
MAPK10	74.388	87.258	23.533	0.853	3.14E-01	False
MAPK1	9.030	11.992	4.561	0.753	3.14E-01	False
LATS2	27.519	32.868	9.953	0.837	3.15E-01	False
CLK3	8.600	10.802	4.237	0.796	3.16E-01	False
SGK2	6.450	8.435	3.577	0.765	3.18E-01	False
PINK1	2877.927	3308.518	820.986	0.870	3.21E-01	False
BMX	7.310	9.554	3.892	0.765	3.23E-01	False
KSR1	350.442	401.914	101.487	0.872	3.28E-01	False
HIPK4	6.020	8.331	3.547	0.723	3.28E-01	False
Non-Targeting_Control_046	25.369	30.170	9.271	0.841	3.30E-01	False
MAK	290.244	332.821	84.380	0.872	3.30E-01	False
TPR	125.557	144.323	37.691	0.870	3.31E-01	False
ACVR1	1719.963	1962.568	487.817	0.876	3.31E-01	False
RFK	27.949	32.381	9.830	0.863	3.32E-01	False
MAST3	6.880	8.279	3.533	0.831	3.33E-01	False
PRKAA2	343.563	392.270	99.099	0.876	3.34E-01	False
CPNE3	2.580	3.583	2.090	0.720	3.34E-01	False
ADCK1	27.089	32.299	9.809	0.839	3.35E-01	False
PIKFYVE	13845.269	15736.496	3897.328	0.880	3.36E-01	False
HK1	43.859	50.434	14.363	0.870	3.38E-01	False
INSR	18.060	21.981	7.182	0.822	3.38E-01	False
MAPK9	6.880	8.233	3.519	0.836	3.38E-01	False
PKN3	113.518	129.921	34.120	0.874	3.38E-01	False
RYK	38.269	44.694	12.926	0.856	3.39E-01	False
EPHA1	0.000	1.117	1.093	0.472	3.39E-01	False
GRK6	0.000	1.117	1.093	0.472	3.39E-01	False
MAP3K4	0.000	1.117	1.093	0.472	3.39E-01	False
MAP3K2	0.000	1.117	1.093	0.472	3.39E-01	False
WNK4	0.000	1.117	1.093	0.472	3.39E-01	False
Non-Targeting_Control_050	0.000	1.117	1.093	0.472	3.39E-01	False
PFKL	69.658	79.824	21.685	0.873	3.40E-01	False
STKLD1	185.756	211.282	54.282	0.879	3.40E-01	False
PFKFB3	2.150	3.533	2.073	0.609	3.42E-01	False
ADCK3	208.545	236.846	60.614	0.881	3.43E-01	False

FGFRL1	371.942	420.564	106.104	0.884	3.45E-01	False
TLK1	13.760	16.128	5.663	0.853	3.46E-01	False
TBK1	0.000	1.090	1.079	0.478	3.48E-01	False
SH3BP4	0.860	1.090	1.079	0.789	3.48E-01	False
NTPCR	0.860	1.087	1.077	0.791	3.49E-01	False
MAP2K5	4.300	5.818	2.809	0.739	3.49E-01	False
HSPB8	14.620	17.200	5.944	0.850	3.50E-01	False
ALK	12.040	14.934	5.348	0.806	3.50E-01	False
ATR	13.330	16.065	5.646	0.830	3.50E-01	False
RIPK1	45.149	52.138	14.788	0.866	3.51E-01	False
Non-Targeting_Control_039	14.620	17.176	5.937	0.851	3.52E-01	False
ACTR2	43.859	49.777	14.198	0.881	3.54E-01	False
NEK10	11.180	13.747	5.033	0.813	3.54E-01	False
NPR2	81.698	92.394	24.808	0.884	3.55E-01	False
TNNI3K	220.155	248.278	63.445	0.887	3.55E-01	False
PRKD3	284.654	319.867	81.173	0.890	3.56E-01	False
PHKA1	337.113	379.190	95.861	0.889	3.56E-01	False
STK10	0.000	1.063	1.064	0.485	3.57E-01	False
CDK11A	0.000	1.063	1.064	0.485	3.57E-01	False
CKS1B	1.290	2.271	1.608	0.568	3.57E-01	False
DGKI	5.590	6.897	3.132	0.810	3.57E-01	False
MAP4K1	11916.761	13334.184	3302.676	0.894	3.59E-01	False
PI4K2B	0.430	1.057	1.061	0.407	3.59E-01	False
SCYL3	7.740	9.132	3.774	0.848	3.61E-01	False
PRPS1L1	49.019	56.108	15.780	0.874	3.63E-01	False
SRM	41.709	47.151	13.542	0.885	3.64E-01	False
ALPK1	342.703	382.271	96.624	0.896	3.66E-01	False
MAP3K12	28.379	32.581	9.880	0.871	3.66E-01	False
TAF1	73.958	82.707	22.402	0.894	3.66E-01	False
RPS6KC1	0.430	1.036	1.050	0.415	3.66E-01	False
MAPK4	7.310	9.078	3.759	0.805	3.66E-01	False
MAP4K4	10.750	12.449	4.685	0.864	3.67E-01	False
GALK1	10.320	12.420	4.677	0.831	3.69E-01	False
MAGI1	0.430	1.026	1.044	0.419	3.70E-01	False
MARK1	0.000	1.026	1.044	0.494	3.70E-01	False
ERBB3	869.441	966.271	241.196	0.900	3.70E-01	False
UHMK1	41.279	46.892	13.477	0.880	3.71E-01	False
EPHA5	16.770	19.089	6.436	0.878	3.71E-01	False
SH3BP5L	332.813	369.418	93.442	0.901	3.72E-01	False
NADK2	1208.704	1339.008	333.463	0.903	3.74E-01	False
MOS	917.600	1016.725	253.685	0.903	3.74E-01	False
RIOK1	76.968	85.537	23.105	0.900	3.74E-01	False
TTBK2	6.020	7.876	3.417	0.764	3.74E-01	False
PRKRA	154.797	171.744	44.487	0.901	3.75E-01	False
NRBP1	0.430	1.009	1.035	0.426	3.76E-01	False
HK2	0.000	1.009	1.035	0.498	3.76E-01	False
BRD3	0.000	1.009	1.035	0.498	3.76E-01	False
PIP4K2B	0.000	1.009	1.035	0.498	3.76E-01	False
CASK	0.000	1.009	1.035	0.498	3.76E-01	False
SCYL1	0.000	1.009	1.035	0.498	3.76E-01	False
MAP4K2	0.000	1.006	1.033	0.499	3.77E-01	False
TESK1	0.000	1.006	1.033	0.499	3.77E-01	False
NME7	6.020	7.851	3.410	0.767	3.77E-01	False
NEK2	8.600	10.084	4.039	0.853	3.77E-01	False
OSR1	4.300	5.601	2.743	0.768	3.77E-01	False
NEK11	3.440	4.469	2.386	0.770	3.78E-01	False
PDXK	0.430	1.003	1.032	0.429	3.78E-01	False
STK26	0.000	1.003	1.032	0.499	3.78E-01	False
ILK	0.860	1.003	1.032	0.858	3.78E-01	False
SGK3	0.860	1.003	1.032	0.858	3.78E-01	False
MAGI3	214.135	237.648	60.812	0.901	3.78E-01	False
LMTK2	0.430	0.999	1.030	0.430	3.79E-01	False
PLK4	0.000	0.999	1.030	0.500	3.79E-01	False
RIOK1	7.740	8.946	3.721	0.865	3.79E-01	False
NEK2	0.000	0.996	1.028	0.501	3.80E-01	False
TRIM24	0.000	0.996	1.028	0.501	3.80E-01	False
MYLK4	0.000	0.996	1.028	0.501	3.80E-01	False
PIM3	0.000	0.996	1.028	0.501	3.80E-01	False
SYK	0.860	0.996	1.028	0.864	3.80E-01	False
CDK15	792.043	874.439	218.463	0.906	3.81E-01	False
NME7	59.769	66.295	18.319	0.902	3.82E-01	False
STK32A	1443.479	1589.023	395.351	0.908	3.83E-01	False
CROT	354.742	390.737	98.720	0.908	3.84E-01	False

ADRBK2	66.219	73.815	20.191	0.897	3.86E-01	False
EPHA3	57.189	63.924	17.729	0.895	3.86E-01	False
NPR1	804.512	883.092	220.605	0.911	3.88E-01	False
UCK1	14.190	16.551	5.774	0.857	3.91E-01	False
FER	23.649	26.406	8.314	0.896	3.92E-01	False
PFKFB4	47.729	52.672	14.922	0.906	3.92E-01	False
TSSK1B	3.870	4.371	2.354	0.885	3.93E-01	False
CAMK2A	9.890	10.985	4.287	0.900	3.96E-01	False
CDC42BPG	354.312	387.476	97.912	0.914	3.96E-01	False
AAK1	396.021	433.137	109.217	0.914	3.97E-01	False
ADK	68.369	75.405	20.586	0.907	3.97E-01	False
DCLK3	7.310	8.748	3.666	0.836	3.99E-01	False
PTK2	133.727	146.023	38.112	0.916	4.00E-01	False
SBK1	16.340	18.559	6.298	0.880	4.02E-01	False
SMG1	12.470	14.175	5.147	0.880	4.03E-01	False
MATK	101.908	110.889	29.399	0.919	4.03E-01	False
PRKCZ	19.350	21.778	7.130	0.889	4.04E-01	False
NEK11	73.528	80.396	21.827	0.915	4.05E-01	False
MAP3K10	10.750	11.968	4.554	0.898	4.05E-01	False
Non-Targeting_Control_079	77.398	84.673	22.890	0.914	4.06E-01	False
IKBKE	182.316	198.423	51.096	0.919	4.06E-01	False
KIT	21.070	23.883	7.670	0.882	4.08E-01	False
PEAK1	77.828	84.491	22.845	0.921	4.09E-01	False
PANK2	1108.516	1199.350	298.892	0.924	4.10E-01	False
SRMS	333.243	361.059	91.372	0.923	4.10E-01	False
FRK	9.030	10.800	4.236	0.836	4.12E-01	False
MEX3B	1.720	2.025	1.509	0.849	4.15E-01	False
CDKL2	239.935	258.410	65.954	0.929	4.17E-01	False
MVK	5.590	6.403	2.986	0.873	4.17E-01	False
ULK4	349.152	376.641	95.230	0.927	4.17E-01	False
PRPS2	82.988	89.360	24.055	0.929	4.17E-01	False
WNK2	8549.074	9197.229	2278.641	0.930	4.18E-01	False
SRPK1	942.539	1014.341	253.095	0.929	4.18E-01	False
PANK4	136.307	147.365	38.445	0.925	4.18E-01	False
ROCK1	13.760	15.041	5.376	0.915	4.19E-01	False
CDKL3	19.780	21.505	7.060	0.920	4.19E-01	False
CSNK1G3	6.450	7.466	3.299	0.864	4.20E-01	False
LIMK1	32.679	35.463	10.608	0.921	4.20E-01	False
STK33	10.320	11.779	4.503	0.876	4.21E-01	False
TWF1	1011.338	1085.625	270.741	0.932	4.22E-01	False
RPS6KA3	82.128	89.039	23.975	0.922	4.22E-01	False
HCK	5.590	6.356	2.972	0.879	4.23E-01	False
ABL2	5.590	6.331	2.964	0.883	4.26E-01	False
TGFBR2	8.600	9.545	3.889	0.901	4.28E-01	False
PCK2	1013.488	1083.107	270.118	0.936	4.28E-01	False
TGFBR3	2.580	3.055	1.904	0.844	4.31E-01	False
ZAP70	3.010	4.135	2.277	0.728	4.32E-01	False
GUCY2F	3.440	4.132	2.275	0.833	4.32E-01	False
CERKL	30.529	32.994	9.985	0.925	4.32E-01	False
ABL2	298.843	318.528	80.841	0.938	4.33E-01	False
PKD1	94.598	101.133	26.978	0.935	4.33E-01	False
ATM	17.200	19.097	6.438	0.901	4.34E-01	False
PMVK	96.318	103.127	27.473	0.934	4.35E-01	False
KSR2	4.730	5.186	2.614	0.912	4.36E-01	False
GSK3A	44.289	47.766	13.695	0.927	4.37E-01	False
TYRO3	79.548	84.931	22.954	0.937	4.37E-01	False
ERN1	3.440	4.099	2.265	0.839	4.38E-01	False
Non-Targeting_Control_068	3417.566	3627.660	899.985	0.942	4.38E-01	False
CDKL5	38.269	41.279	12.070	0.927	4.40E-01	False
ADRBK2	33.539	35.975	10.737	0.932	4.40E-01	False
DLG1	8.170	9.418	3.854	0.867	4.41E-01	False
Non-Targeting_Control_097	1370.810	1452.308	361.509	0.944	4.41E-01	False
MAP2K4	0.000	0.838	0.939	0.544	4.42E-01	False
WEE1	0.000	0.838	0.939	0.544	4.42E-01	False
SMG1	0.000	0.838	0.939	0.544	4.42E-01	False
SCYL3	0.000	0.838	0.939	0.544	4.42E-01	False
FER	5.590	6.211	2.928	0.900	4.42E-01	False
CAMK1D	3.870	4.071	2.255	0.951	4.42E-01	False
RPS6KA6	1192.364	1261.986	314.397	0.945	4.43E-01	False
RAB11FIP5	161.676	171.253	44.365	0.944	4.44E-01	False
LIMK2	31.819	33.742	10.174	0.943	4.44E-01	False
MKNK2	1190.214	1258.150	313.448	0.946	4.46E-01	False
MAPKAPK3	301.853	318.921	80.938	0.946	4.46E-01	False

PRKG1	3.870	4.044	2.246	0.957	4.47E-01	False
ALPK2	1.720	1.901	1.457	0.905	4.47E-01	False
Non-Targeting_Control_071	252.834	266.722	68.013	0.948	4.48E-01	False
TIE1	59.339	63.157	17.538	0.940	4.49E-01	False
PRKD1	15.050	16.740	5.823	0.899	4.49E-01	False
GRK5	909.860	959.147	239.432	0.949	4.49E-01	False
CKS2	10.320	11.435	4.410	0.902	4.51E-01	False
LTK	0.000	0.811	0.923	0.552	4.53E-01	False
PANK3	0.000	0.811	0.923	0.552	4.53E-01	False
PTK2	0.000	0.808	0.921	0.553	4.55E-01	False
PTK7	20.640	21.873	7.154	0.944	4.57E-01	False
PSKH1	6.020	7.133	3.202	0.844	4.59E-01	False
XYLB	3.440	3.967	2.221	0.867	4.60E-01	False
KIT	86.858	90.763	24.403	0.957	4.63E-01	False
MAP3K14	10.750	11.298	4.373	0.951	4.63E-01	False
MKNK1	1096.046	1145.245	285.499	0.957	4.64E-01	False
PIP5KL1	16.770	17.554	6.036	0.955	4.64E-01	False
ACVR2B	0.000	0.784	0.906	0.561	4.65E-01	False
CSNK1A1	0.000	0.784	0.906	0.561	4.65E-01	False
IDNK	0.000	0.784	0.906	0.561	4.65E-01	False
PLXNA4	0.860	0.784	0.906	1.097	4.65E-01	False
YES1	2.580	2.890	1.845	0.893	4.65E-01	False
AK8	4.730	4.980	2.549	0.950	4.67E-01	False
PIK3C2G	0.000	0.777	0.902	0.563	4.68E-01	False
TK2	0.000	0.777	0.902	0.563	4.68E-01	False
GNE	0.000	0.777	0.902	0.563	4.68E-01	False
PXK	0.000	0.777	0.902	0.563	4.68E-01	False
GSK3A	872.021	908.413	226.873	0.960	4.69E-01	False
NRBP1	25.799	26.827	8.422	0.962	4.70E-01	False
MKNK1	27.089	28.902	8.949	0.937	4.71E-01	False
PDK3	8.170	9.125	3.772	0.895	4.71E-01	False
STYK1	750.764	780.492	195.207	0.962	4.71E-01	False
PI4KB	8.600	9.118	3.770	0.943	4.72E-01	False
MYO3A	27.519	28.845	8.934	0.954	4.73E-01	False
PAPSS1	14.620	15.340	5.455	0.953	4.73E-01	False
PIP5K1C	98.898	102.545	27.329	0.964	4.73E-01	False
DCLK1	47.299	49.592	14.152	0.954	4.74E-01	False
HCK	1274.062	1321.811	329.206	0.964	4.75E-01	False
RAB11FIP5	8.170	9.084	3.760	0.899	4.75E-01	False
DGKK	146.627	152.060	39.608	0.964	4.76E-01	False
MAPK15	19.350	20.475	6.794	0.945	4.77E-01	False
SPEG	0.430	0.757	0.890	0.568	4.77E-01	False
ADRBK2	0.000	0.757	0.890	0.569	4.77E-01	False
CKB	0.000	0.757	0.890	0.569	4.77E-01	False
PLK3	0.000	0.757	0.890	0.569	4.77E-01	False
KDR	0.000	0.757	0.890	0.569	4.77E-01	False
PRKAR1A	0.000	0.757	0.890	0.569	4.77E-01	False
PTK2	0.000	0.757	0.890	0.569	4.77E-01	False
NME5	0.000	0.757	0.890	0.569	4.77E-01	False
MAP4K3	0.000	0.757	0.890	0.569	4.77E-01	False
TAOK3	0.000	0.757	0.890	0.569	4.77E-01	False
Non-Targeting_Control_032	0.000	0.757	0.890	0.569	4.77E-01	False
STK4	8.600	9.054	3.752	0.950	4.78E-01	False
PDK2	0.000	0.750	0.886	0.571	4.80E-01	False
FGR	0.430	0.750	0.886	0.573	4.80E-01	False
MAP3K6	0.430	0.750	0.886	0.573	4.80E-01	False
DYRK2	0.860	0.750	0.886	1.146	4.80E-01	False
GK5	1008.328	1042.540	260.075	0.967	4.80E-01	False
TLK1	22.790	23.489	7.569	0.970	4.82E-01	False
CSNK1E	287.664	297.115	75.539	0.968	4.82E-01	False
AK1	0.000	0.747	0.884	0.572	4.82E-01	False
MST1	0.000	0.747	0.884	0.572	4.82E-01	False
PKD1	0.000	0.747	0.884	0.572	4.82E-01	False
PRKACA	0.000	0.747	0.884	0.572	4.82E-01	False
PIP5K1C	0.000	0.747	0.884	0.572	4.82E-01	False
PI4K2B	0.000	0.747	0.884	0.572	4.82E-01	False
LRRK1	0.000	0.747	0.884	0.572	4.82E-01	False
VRK2	0.860	0.747	0.884	1.152	4.82E-01	False
RPS6KC1	0.860	0.747	0.884	1.152	4.82E-01	False
MAST2	2680.562	2760.796	685.406	0.971	4.86E-01	False
FASTKD5	2.580	2.794	1.809	0.924	4.86E-01	False
FPGT-TNNI3K	1.290	1.760	1.396	0.733	4.87E-01	False
PIK3C2A	1.720	1.756	1.395	0.979	4.88E-01	False

FASTKD2	23.649	24.376	7.796	0.970	4.89E-01	False
CHEK2	111.368	114.736	30.354	0.971	4.90E-01	False
CDK6	4.730	4.828	2.501	0.980	4.90E-01	False
Non-Targeting_Control_052	518.999	532.508	133.818	0.975	4.91E-01	False
SLK	37.409	38.688	11.419	0.967	4.92E-01	False
KIAA1804	436.870	448.060	112.912	0.975	4.92E-01	False
STK32C	44.289	45.863	13.219	0.966	4.92E-01	False
PANK1	821.282	842.845	210.643	0.974	4.92E-01	False
CDK14	891.371	914.605	228.406	0.975	4.92E-01	False
DGKQ	9.890	9.942	4.000	0.995	4.93E-01	False
TRIM24	2.150	2.762	1.797	0.778	4.93E-01	False
WEE2	16.340	17.120	5.923	0.954	4.93E-01	False
MARK1	11.610	11.991	4.561	0.968	4.93E-01	False
ACVR2B	12.470	13.013	4.836	0.958	4.93E-01	False
Non-Targeting_Control_025	68.799	70.373	19.334	0.978	4.94E-01	False
AK3	208.975	213.727	54.887	0.978	4.95E-01	False
BUB1	2.150	2.752	1.793	0.781	4.95E-01	False
PRKD3	18.060	19.131	6.446	0.944	4.95E-01	False
ACVRL1	9.030	9.901	3.989	0.912	4.97E-01	False
Non-Targeting_Control_049	52.889	53.832	15.212	0.982	4.98E-01	False
YES1	2.150	2.738	1.788	0.785	4.98E-01	False
ACVR1	0.000	0.000	0.000	1.000	5.00E-01	False
ACVR2A	0.000	0.000	0.000	1.000	5.00E-01	False
AK4	0.000	0.000	0.000	1.000	5.00E-01	False
AKT2	0.000	0.000	0.000	1.000	5.00E-01	False
AMHR2	0.000	0.000	0.000	1.000	5.00E-01	False
ARAF	0.000	0.000	0.000	1.000	5.00E-01	False
ATM	0.000	0.000	0.000	1.000	5.00E-01	False
ATM	0.000	0.000	0.000	1.000	5.00E-01	False
ATR	0.000	0.000	0.000	1.000	5.00E-01	False
BLK	0.000	0.000	0.000	1.000	5.00E-01	False
BMPR1A	0.000	0.000	0.000	1.000	5.00E-01	False
BMPR1B	0.000	0.000	0.000	1.000	5.00E-01	False
BRAF	0.000	0.000	0.000	1.000	5.00E-01	False
BUB1	0.000	0.000	0.000	1.000	5.00E-01	False
BUB1	0.000	0.000	0.000	1.000	5.00E-01	False
BUB1B	0.000	0.000	0.000	1.000	5.00E-01	False
BUB1B	0.000	0.000	0.000	1.000	5.00E-01	False
BUB1B	0.000	0.000	0.000	1.000	5.00E-01	False
CAD	0.000	0.000	0.000	1.000	5.00E-01	False
CAD	0.000	0.000	0.000	1.000	5.00E-01	False
CALM1	0.000	0.000	0.000	1.000	5.00E-01	False
CALM1	0.000	0.000	0.000	1.000	5.00E-01	False
CAMK2B	0.000	0.000	0.000	1.000	5.00E-01	False
CAMK2G	0.000	0.000	0.000	1.000	5.00E-01	False
CAMK2G	0.000	0.000	0.000	1.000	5.00E-01	False
CDK1	0.000	0.000	0.000	1.000	5.00E-01	False
CDK1	0.000	0.000	0.000	1.000	5.00E-01	False
CDK1	0.000	0.000	0.000	1.000	5.00E-01	False
CDK1	0.000	0.000	0.000	1.000	5.00E-01	False
CDK11B	0.000	0.000	0.000	1.000	5.00E-01	False
CDK11B	0.000	0.000	0.000	1.000	5.00E-01	False
CDK6	0.000	0.000	0.000	1.000	5.00E-01	False
CDK7	0.000	0.000	0.000	1.000	5.00E-01	False
CDK9	0.000	0.000	0.000	1.000	5.00E-01	False
CDK9	0.000	0.000	0.000	1.000	5.00E-01	False
CHEK1	0.000	0.000	0.000	1.000	5.00E-01	False
CHEK1	0.000	0.000	0.000	1.000	5.00E-01	False
CHEK1	0.000	0.000	0.000	1.000	5.00E-01	False
CHKA	0.000	0.000	0.000	1.000	5.00E-01	False
CHUK	0.000	0.000	0.000	1.000	5.00E-01	False
CHUK	0.000	0.000	0.000	1.000	5.00E-01	False
CKB	0.000	0.000	0.000	1.000	5.00E-01	False
CKM	0.000	0.000	0.000	1.000	5.00E-01	False
CKM	0.000	0.000	0.000	1.000	5.00E-01	False
CKMT1B	0.000	0.000	0.000	1.000	5.00E-01	False
CKMT2	0.000	0.000	0.000	1.000	5.00E-01	False
CKS1B	0.000	0.000	0.000	1.000	5.00E-01	False
CKS2	0.000	0.000	0.000	1.000	5.00E-01	False
CLK1	0.000	0.000	0.000	1.000	5.00E-01	False
CLK3	0.000	0.000	0.000	1.000	5.00E-01	False
CLK3	0.000	0.000	0.000	1.000	5.00E-01	False
PLK3	0.000	0.000	0.000	1.000	5.00E-01	False

MAPK14	0.000	0.000	0.000	1.000	5.00E-01	False
CSF1R	0.000	0.000	0.000	1.000	5.00E-01	False
CSNK1A1	0.000	0.000	0.000	1.000	5.00E-01	False
CSNK1A1	0.000	0.000	0.000	1.000	5.00E-01	False
CSNK1D	0.000	0.000	0.000	1.000	5.00E-01	False
CSNK2A1	0.000	0.000	0.000	1.000	5.00E-01	False
CSNK2B	0.000	0.000	0.000	1.000	5.00E-01	False
CSNK2B	0.000	0.000	0.000	1.000	5.00E-01	False
DGKA	0.000	0.000	0.000	1.000	5.00E-01	False
DGKG	0.000	0.000	0.000	1.000	5.00E-01	False
DGUOK	0.000	0.000	0.000	1.000	5.00E-01	False
DLG1	0.000	0.000	0.000	1.000	5.00E-01	False
DLG1	0.000	0.000	0.000	1.000	5.00E-01	False
DLG3	0.000	0.000	0.000	1.000	5.00E-01	False
DTYMK	0.000	0.000	0.000	1.000	5.00E-01	False
DYRK1A	0.000	0.000	0.000	1.000	5.00E-01	False
DYRK1A	0.000	0.000	0.000	1.000	5.00E-01	False
EGFR	0.000	0.000	0.000	1.000	5.00E-01	False
EPHA5	0.000	0.000	0.000	1.000	5.00E-01	False
EPHB1	0.000	0.000	0.000	1.000	5.00E-01	False
ERN1	0.000	0.000	0.000	1.000	5.00E-01	False
FGFR2	0.000	0.000	0.000	1.000	5.00E-01	False
FLT4	0.000	0.000	0.000	1.000	5.00E-01	False
FXN	0.000	0.000	0.000	1.000	5.00E-01	False
FXN	0.000	0.000	0.000	1.000	5.00E-01	False
MTOR	0.000	0.000	0.000	1.000	5.00E-01	False
MTOR	0.000	0.000	0.000	1.000	5.00E-01	False
GAK	0.000	0.000	0.000	1.000	5.00E-01	False
GALK1	0.000	0.000	0.000	1.000	5.00E-01	False
GK	0.000	0.000	0.000	1.000	5.00E-01	False
GK2	0.000	0.000	0.000	1.000	5.00E-01	False
GRK5	0.000	0.000	0.000	1.000	5.00E-01	False
GSK3A	0.000	0.000	0.000	1.000	5.00E-01	False
GSK3B	0.000	0.000	0.000	1.000	5.00E-01	False
GTF2H1	0.000	0.000	0.000	1.000	5.00E-01	False
GTF2H1	0.000	0.000	0.000	1.000	5.00E-01	False
GUCY2C	0.000	0.000	0.000	1.000	5.00E-01	False
GUCY2F	0.000	0.000	0.000	1.000	5.00E-01	False
GUK1	0.000	0.000	0.000	1.000	5.00E-01	False
GUK1	0.000	0.000	0.000	1.000	5.00E-01	False
GUCY2D	0.000	0.000	0.000	1.000	5.00E-01	False
HUS1	0.000	0.000	0.000	1.000	5.00E-01	False
IGF1R	0.000	0.000	0.000	1.000	5.00E-01	False
IKBKB	0.000	0.000	0.000	1.000	5.00E-01	False
ILK	0.000	0.000	0.000	1.000	5.00E-01	False
ILK	0.000	0.000	0.000	1.000	5.00E-01	False
IRAK1	0.000	0.000	0.000	1.000	5.00E-01	False
ITPK1	0.000	0.000	0.000	1.000	5.00E-01	False
ITPK1	0.000	0.000	0.000	1.000	5.00E-01	False
ITPK1	0.000	0.000	0.000	1.000	5.00E-01	False
ITPKB	0.000	0.000	0.000	1.000	5.00E-01	False
JAK3	0.000	0.000	0.000	1.000	5.00E-01	False
KIT	0.000	0.000	0.000	1.000	5.00E-01	False
LCK	0.000	0.000	0.000	1.000	5.00E-01	False
LTK	0.000	0.000	0.000	1.000	5.00E-01	False
LTK	0.000	0.000	0.000	1.000	5.00E-01	False
LTK	0.000	0.000	0.000	1.000	5.00E-01	False
MATK	0.000	0.000	0.000	1.000	5.00E-01	False
MAP3K1	0.000	0.000	0.000	1.000	5.00E-01	False
MAP3K9	0.000	0.000	0.000	1.000	5.00E-01	False
MOS	0.000	0.000	0.000	1.000	5.00E-01	False
MPP1	0.000	0.000	0.000	1.000	5.00E-01	False
MPP3	0.000	0.000	0.000	1.000	5.00E-01	False
ABCC1	0.000	0.000	0.000	1.000	5.00E-01	False
MVK	0.000	0.000	0.000	1.000	5.00E-01	False
MVK	0.000	0.000	0.000	1.000	5.00E-01	False
NME3	0.000	0.000	0.000	1.000	5.00E-01	False
NME4	0.000	0.000	0.000	1.000	5.00E-01	False
NPR1	0.000	0.000	0.000	1.000	5.00E-01	False
NPR2	0.000	0.000	0.000	1.000	5.00E-01	False
NTRK3	0.000	0.000	0.000	1.000	5.00E-01	False
NTRK3	0.000	0.000	0.000	1.000	5.00E-01	False
PAK3	0.000	0.000	0.000	1.000	5.00E-01	False

PCK1	0.000	0.000	0.000	1.000	5.00E-01	False
PDGFRA	0.000	0.000	0.000	1.000	5.00E-01	False
PDGFRA	0.000	0.000	0.000	1.000	5.00E-01	False
PDK1	0.000	0.000	0.000	1.000	5.00E-01	False
PDK2	0.000	0.000	0.000	1.000	5.00E-01	False
PDPK1	0.000	0.000	0.000	1.000	5.00E-01	False
PFKL	0.000	0.000	0.000	1.000	5.00E-01	False
PGK1	0.000	0.000	0.000	1.000	5.00E-01	False
PGK1	0.000	0.000	0.000	1.000	5.00E-01	False
PHKA1	0.000	0.000	0.000	1.000	5.00E-01	False
PIK3C2A	0.000	0.000	0.000	1.000	5.00E-01	False
PIK3C3	0.000	0.000	0.000	1.000	5.00E-01	False
PIK3C3	0.000	0.000	0.000	1.000	5.00E-01	False
PI4KB	0.000	0.000	0.000	1.000	5.00E-01	False
PKM	0.000	0.000	0.000	1.000	5.00E-01	False
PKM	0.000	0.000	0.000	1.000	5.00E-01	False
PLAU	0.000	0.000	0.000	1.000	5.00E-01	False
PLK1	0.000	0.000	0.000	1.000	5.00E-01	False
PLK1	0.000	0.000	0.000	1.000	5.00E-01	False
EXOSC10	0.000	0.000	0.000	1.000	5.00E-01	False
EXOSC10	0.000	0.000	0.000	1.000	5.00E-01	False
PRKAB2	0.000	0.000	0.000	1.000	5.00E-01	False
PRKACB	0.000	0.000	0.000	1.000	5.00E-01	False
PRKAR1A	0.000	0.000	0.000	1.000	5.00E-01	False
PRKAR2A	0.000	0.000	0.000	1.000	5.00E-01	False
PRKCA	0.000	0.000	0.000	1.000	5.00E-01	False
PRKCE	0.000	0.000	0.000	1.000	5.00E-01	False
PRKCH	0.000	0.000	0.000	1.000	5.00E-01	False
PRKCI	0.000	0.000	0.000	1.000	5.00E-01	False
PKN1	0.000	0.000	0.000	1.000	5.00E-01	False
PKN2	0.000	0.000	0.000	1.000	5.00E-01	False
PRKD1	0.000	0.000	0.000	1.000	5.00E-01	False
PRKCZ	0.000	0.000	0.000	1.000	5.00E-01	False
PRKDC	0.000	0.000	0.000	1.000	5.00E-01	False
PRKG2	0.000	0.000	0.000	1.000	5.00E-01	False
PRKG2	0.000	0.000	0.000	1.000	5.00E-01	False
MAPK6	0.000	0.000	0.000	1.000	5.00E-01	False
MAPK6	0.000	0.000	0.000	1.000	5.00E-01	False
MAPK8	0.000	0.000	0.000	1.000	5.00E-01	False
MAPK13	0.000	0.000	0.000	1.000	5.00E-01	False
MAP2K3	0.000	0.000	0.000	1.000	5.00E-01	False
MAP2K3	0.000	0.000	0.000	1.000	5.00E-01	False
MAP2K6	0.000	0.000	0.000	1.000	5.00E-01	False
MAP2K7	0.000	0.000	0.000	1.000	5.00E-01	False
PRKX	0.000	0.000	0.000	1.000	5.00E-01	False
PSKH1	0.000	0.000	0.000	1.000	5.00E-01	False
PTK2	0.000	0.000	0.000	1.000	5.00E-01	False
ALDH18A1	0.000	0.000	0.000	1.000	5.00E-01	False
MOK	0.000	0.000	0.000	1.000	5.00E-01	False
RAF1	0.000	0.000	0.000	1.000	5.00E-01	False
RET	0.000	0.000	0.000	1.000	5.00E-01	False
TRIM27	0.000	0.000	0.000	1.000	5.00E-01	False
BRD2	0.000	0.000	0.000	1.000	5.00E-01	False
BRD2	0.000	0.000	0.000	1.000	5.00E-01	False
RPS6KB1	0.000	0.000	0.000	1.000	5.00E-01	False
RPS6KB2	0.000	0.000	0.000	1.000	5.00E-01	False
RPS6KB2	0.000	0.000	0.000	1.000	5.00E-01	False
MAPK12	0.000	0.000	0.000	1.000	5.00E-01	False
MAPK12	0.000	0.000	0.000	1.000	5.00E-01	False
SRPK1	0.000	0.000	0.000	1.000	5.00E-01	False
STK3	0.000	0.000	0.000	1.000	5.00E-01	False
AURKA	0.000	0.000	0.000	1.000	5.00E-01	False
AURKA	0.000	0.000	0.000	1.000	5.00E-01	False
STK11	0.000	0.000	0.000	1.000	5.00E-01	False
MAP3K7	0.000	0.000	0.000	1.000	5.00E-01	False
MAP3K7	0.000	0.000	0.000	1.000	5.00E-01	False
MAP3K7	0.000	0.000	0.000	1.000	5.00E-01	False
TEK	0.000	0.000	0.000	1.000	5.00E-01	False
TTK	0.000	0.000	0.000	1.000	5.00E-01	False
TTN	0.000	0.000	0.000	1.000	5.00E-01	False
VRK1	0.000	0.000	0.000	1.000	5.00E-01	False
WEE1	0.000	0.000	0.000	1.000	5.00E-01	False
MAP3K12	0.000	0.000	0.000	1.000	5.00E-01	False

TRRAP	0.000	0.000	0.000	1.000	5.00E-01	False
TRRAP	0.000	0.000	0.000	1.000	5.00E-01	False
CDC7	0.000	0.000	0.000	1.000	5.00E-01	False
CDC7	0.000	0.000	0.000	1.000	5.00E-01	False
CDC7	0.000	0.000	0.000	1.000	5.00E-01	False
PIP5K1B	0.000	0.000	0.000	1.000	5.00E-01	False
PIP5K1B	0.000	0.000	0.000	1.000	5.00E-01	False
DYRK3	0.000	0.000	0.000	1.000	5.00E-01	False
MAPKAPK5	0.000	0.000	0.000	1.000	5.00E-01	False
CASK	0.000	0.000	0.000	1.000	5.00E-01	False
CASK	0.000	0.000	0.000	1.000	5.00E-01	False
LAMTOR3	0.000	0.000	0.000	1.000	5.00E-01	False
TRIM24	0.000	0.000	0.000	1.000	5.00E-01	False
PRPF4B	0.000	0.000	0.000	1.000	5.00E-01	False
MAP3K14	0.000	0.000	0.000	1.000	5.00E-01	False
MAP3K14	0.000	0.000	0.000	1.000	5.00E-01	False
BRSK2	0.000	0.000	0.000	1.000	5.00E-01	False
BAZ1B	0.000	0.000	0.000	1.000	5.00E-01	False
PAPSS1	0.000	0.000	0.000	1.000	5.00E-01	False
PKMYT1	0.000	0.000	0.000	1.000	5.00E-01	False
PKMYT1	0.000	0.000	0.000	1.000	5.00E-01	False
AURKB	0.000	0.000	0.000	1.000	5.00E-01	False
RPS6KA5	0.000	0.000	0.000	1.000	5.00E-01	False
TAOK2	0.000	0.000	0.000	1.000	5.00E-01	False
TJP2	0.000	0.000	0.000	1.000	5.00E-01	False
ROCK2	0.000	0.000	0.000	1.000	5.00E-01	False
CDC42BPB	0.000	0.000	0.000	1.000	5.00E-01	False
MELK	0.000	0.000	0.000	1.000	5.00E-01	False
TLK1	0.000	0.000	0.000	1.000	5.00E-01	False
GNE	0.000	0.000	0.000	1.000	5.00E-01	False
COL4A3BP	0.000	0.000	0.000	1.000	5.00E-01	False
COL4A3BP	0.000	0.000	0.000	1.000	5.00E-01	False
COL4A3BP	0.000	0.000	0.000	1.000	5.00E-01	False
ACTR2	0.000	0.000	0.000	1.000	5.00E-01	False
TRIM28	0.000	0.000	0.000	1.000	5.00E-01	False
TRIM28	0.000	0.000	0.000	1.000	5.00E-01	False
NME6	0.000	0.000	0.000	1.000	5.00E-01	False
NME6	0.000	0.000	0.000	1.000	5.00E-01	False
NME6	0.000	0.000	0.000	1.000	5.00E-01	False
TRIB1	0.000	0.000	0.000	1.000	5.00E-01	False
TRIB1	0.000	0.000	0.000	1.000	5.00E-01	False
CIB1	0.000	0.000	0.000	1.000	5.00E-01	False
CIB1	0.000	0.000	0.000	1.000	5.00E-01	False
PMVK	0.000	0.000	0.000	1.000	5.00E-01	False
PMVK	0.000	0.000	0.000	1.000	5.00E-01	False
PLK4	0.000	0.000	0.000	1.000	5.00E-01	False
MAP3K2	0.000	0.000	0.000	1.000	5.00E-01	False
MAP3K2	0.000	0.000	0.000	1.000	5.00E-01	False
CLP1	0.000	0.000	0.000	1.000	5.00E-01	False
CLP1	0.000	0.000	0.000	1.000	5.00E-01	False
CLP1	0.000	0.000	0.000	1.000	5.00E-01	False
TLK2	0.000	0.000	0.000	1.000	5.00E-01	False
IRAK3	0.000	0.000	0.000	1.000	5.00E-01	False
PNKP	0.000	0.000	0.000	1.000	5.00E-01	False
PNKP	0.000	0.000	0.000	1.000	5.00E-01	False
SEPHS2	0.000	0.000	0.000	1.000	5.00E-01	False
MAST2	0.000	0.000	0.000	1.000	5.00E-01	False
PASK	0.000	0.000	0.000	1.000	5.00E-01	False
PPIP5K2	0.000	0.000	0.000	1.000	5.00E-01	False
PPIP5K2	0.000	0.000	0.000	1.000	5.00E-01	False
ATMIN	0.000	0.000	0.000	1.000	5.00E-01	False
BRD4	0.000	0.000	0.000	1.000	5.00E-01	False
CDK20	0.000	0.000	0.000	1.000	5.00E-01	False
CDK20	0.000	0.000	0.000	1.000	5.00E-01	False
DAPK2	0.000	0.000	0.000	1.000	5.00E-01	False
TSSK2	0.000	0.000	0.000	1.000	5.00E-01	False
NUP62	0.000	0.000	0.000	1.000	5.00E-01	False
DSTYK	0.000	0.000	0.000	1.000	5.00E-01	False
AK5	0.000	0.000	0.000	1.000	5.00E-01	False
EIF2AK1	0.000	0.000	0.000	1.000	5.00E-01	False
STK36	0.000	0.000	0.000	1.000	5.00E-01	False
TRIB2	0.000	0.000	0.000	1.000	5.00E-01	False
EEF2K	0.000	0.000	0.000	1.000	5.00E-01	False

NRBP1	0.000	0.000	0.000	1.000	5.00E-01	False
HUNK	0.000	0.000	0.000	1.000	5.00E-01	False
TNNI3K	0.000	0.000	0.000	1.000	5.00E-01	False
NME8	0.000	0.000	0.000	1.000	5.00E-01	False
MPP6	0.000	0.000	0.000	1.000	5.00E-01	False
NLK	0.000	0.000	0.000	1.000	5.00E-01	False
CMPK1	0.000	0.000	0.000	1.000	5.00E-01	False
CDK12	0.000	0.000	0.000	1.000	5.00E-01	False
FGFRL1	0.000	0.000	0.000	1.000	5.00E-01	False
TRPM7	0.000	0.000	0.000	1.000	5.00E-01	False
TRPM7	0.000	0.000	0.000	1.000	5.00E-01	False
TRPM7	0.000	0.000	0.000	1.000	5.00E-01	False
SNRK	0.000	0.000	0.000	1.000	5.00E-01	False
SNRK	0.000	0.000	0.000	1.000	5.00E-01	False
ULK4	0.000	0.000	0.000	1.000	5.00E-01	False
ULK4	0.000	0.000	0.000	1.000	5.00E-01	False
ETNK2	0.000	0.000	0.000	1.000	5.00E-01	False
FGGY	0.000	0.000	0.000	1.000	5.00E-01	False
RFK	0.000	0.000	0.000	1.000	5.00E-01	False
STK32B	0.000	0.000	0.000	1.000	5.00E-01	False
NAGK	0.000	0.000	0.000	1.000	5.00E-01	False
N4BP2	0.000	0.000	0.000	1.000	5.00E-01	False
AGK	0.000	0.000	0.000	1.000	5.00E-01	False
PBK	0.000	0.000	0.000	1.000	5.00E-01	False
TEX14	0.000	0.000	0.000	1.000	5.00E-01	False
STK31	0.000	0.000	0.000	1.000	5.00E-01	False
PAK7	0.000	0.000	0.000	1.000	5.00E-01	False
CAMK1G	0.000	0.000	0.000	1.000	5.00E-01	False
SCYL1	0.000	0.000	0.000	1.000	5.00E-01	False
MARK4	0.000	0.000	0.000	1.000	5.00E-01	False
RBKS	0.000	0.000	0.000	1.000	5.00E-01	False
SNX16	0.000	0.000	0.000	1.000	5.00E-01	False
C17orf75	0.000	0.000	0.000	1.000	5.00E-01	False
CERK	0.000	0.000	0.000	1.000	5.00E-01	False
WNK1	0.000	0.000	0.000	1.000	5.00E-01	False
WNK4	0.000	0.000	0.000	1.000	5.00E-01	False
LRRK1	0.000	0.000	0.000	1.000	5.00E-01	False
NOL9	0.000	0.000	0.000	1.000	5.00E-01	False
NOL9	0.000	0.000	0.000	1.000	5.00E-01	False
DCAKD	0.000	0.000	0.000	1.000	5.00E-01	False
DCAKD	0.000	0.000	0.000	1.000	5.00E-01	False
ADCK4	0.000	0.000	0.000	1.000	5.00E-01	False
COASY	0.000	0.000	0.000	1.000	5.00E-01	False
COASY	0.000	0.000	0.000	1.000	5.00E-01	False
COASY	0.000	0.000	0.000	1.000	5.00E-01	False
NUAK2	0.000	0.000	0.000	1.000	5.00E-01	False
RIOK1	0.000	0.000	0.000	1.000	5.00E-01	False
GSG2	0.000	0.000	0.000	1.000	5.00E-01	False
GSG2	0.000	0.000	0.000	1.000	5.00E-01	False
TSSK6	0.000	0.000	0.000	1.000	5.00E-01	False
MEX3B	0.000	0.000	0.000	1.000	5.00E-01	False
NTPCR	0.000	0.000	0.000	1.000	5.00E-01	False
CARD11	0.000	0.000	0.000	1.000	5.00E-01	False
BRSK1	0.000	0.000	0.000	1.000	5.00E-01	False
MASTL	0.000	0.000	0.000	1.000	5.00E-01	False
MASTL	0.000	0.000	0.000	1.000	5.00E-01	False
MYLK2	0.000	0.000	0.000	1.000	5.00E-01	False
ADCK2	0.000	0.000	0.000	1.000	5.00E-01	False
ADCK2	0.000	0.000	0.000	1.000	5.00E-01	False
XRCC6BP1	0.000	0.000	0.000	1.000	5.00E-01	False
PLXNA4	0.000	0.000	0.000	1.000	5.00E-01	False
STRADA	0.000	0.000	0.000	1.000	5.00E-01	False
TBCK	0.000	0.000	0.000	1.000	5.00E-01	False
TP53RK	0.000	0.000	0.000	1.000	5.00E-01	False
TP53RK	0.000	0.000	0.000	1.000	5.00E-01	False
LMTK3	0.000	0.000	0.000	1.000	5.00E-01	False
SLAMF6	0.000	0.000	0.000	1.000	5.00E-01	False
SGK494	0.000	0.000	0.000	1.000	5.00E-01	False
PLK5	0.000	0.000	0.000	1.000	5.00E-01	False
UHMK1	0.000	0.000	0.000	1.000	5.00E-01	False
GRK7	0.000	0.000	0.000	1.000	5.00E-01	False
NADK2	0.000	0.000	0.000	1.000	5.00E-01	False
DGKK	0.000	0.000	0.000	1.000	5.00E-01	False

PNCK	0.000	0.000	0.000	1.000	5.00E-01	False
NEK7	0.000	0.000	0.000	1.000	5.00E-01	False
NEK7	0.000	0.000	0.000	1.000	5.00E-01	False
STK35	0.000	0.000	0.000	1.000	5.00E-01	False
MPP7	0.000	0.000	0.000	1.000	5.00E-01	False
SIK1	0.000	0.000	0.000	1.000	5.00E-01	False
SGK223	0.000	0.000	0.000	1.000	5.00E-01	False
DGKH	0.000	0.000	0.000	1.000	5.00E-01	False
STKLD1	0.000	0.000	0.000	1.000	5.00E-01	False
FUK	0.000	0.000	0.000	1.000	5.00E-01	False
STK32A	0.000	0.000	0.000	1.000	5.00E-01	False
ADCK5	0.000	0.000	0.000	1.000	5.00E-01	False
HIPK1	0.000	0.000	0.000	1.000	5.00E-01	False
HIPK1	0.000	0.000	0.000	1.000	5.00E-01	False
MAGI3	0.000	0.000	0.000	1.000	5.00E-01	False
PGM2L1	0.000	0.000	0.000	1.000	5.00E-01	False
TSSK4	0.000	0.000	0.000	1.000	5.00E-01	False
EPHA6	0.000	0.000	0.000	1.000	5.00E-01	False
NME9	0.000	0.000	0.000	1.000	5.00E-01	False
CERKL	0.000	0.000	0.000	1.000	5.00E-01	False
MAST4	0.000	0.000	0.000	1.000	5.00E-01	False
MAP3K15	0.000	0.000	0.000	1.000	5.00E-01	False
PIM3	0.000	0.000	0.000	1.000	5.00E-01	False
EIF2AK4	0.000	0.000	0.000	1.000	5.00E-01	False
WEE2	0.000	0.000	0.000	1.000	5.00E-01	False
CKMT1A	0.000	0.000	0.000	1.000	5.00E-01	False
NME1-NME2	0.000	0.000	0.000	1.000	5.00E-01	False
NME1-NME2	0.000	0.000	0.000	1.000	5.00E-01	False
Non-Targeting_Control_027	0.000	0.000	0.000	1.000	5.00E-01	False
Non-Targeting_Control_033	0.000	0.000	0.000	1.000	5.00E-01	False
Non-Targeting_Control_057	0.000	0.000	0.000	1.000	5.00E-01	False
Non-Targeting_Control_064	0.000	0.000	0.000	1.000	5.00E-01	False
Non-Targeting_Control_084	0.000	0.000	0.000	1.000	5.00E-01	False
CHUK	0.430	0.000	0.000	1.430	5.00E-01	False
DMPK	0.430	0.000	0.000	1.430	5.00E-01	False
GRK4	0.430	0.000	0.000	1.430	5.00E-01	False
STK11	0.430	0.000	0.000	1.430	5.00E-01	False
TYK2	0.430	0.000	0.000	1.430	5.00E-01	False
EIF2AK1	0.430	0.000	0.000	1.430	5.00E-01	False
CROT	0.430	0.000	0.000	1.430	5.00E-01	False
ADCK3	0.430	0.000	0.000	1.430	5.00E-01	False
FN3KRP	0.430	0.000	0.000	1.430	5.00E-01	False
FPGT-TNNI3K	0.430	0.000	0.000	1.430	5.00E-01	False
DYRK1A	0.860	0.000	0.000	1.860	5.00E-01	False
DDR2	0.860	0.000	0.000	1.860	5.00E-01	False
PAK1	0.860	0.000	0.000	1.860	5.00E-01	False
PRKCQ	0.860	0.000	0.000	1.860	5.00E-01	False
NUAK1	0.860	0.000	0.000	1.860	5.00E-01	False
TWF2	0.860	0.000	0.000	1.860	5.00E-01	False
MPP6	0.860	0.000	0.000	1.860	5.00E-01	False
SCYL1	0.860	0.000	0.000	1.860	5.00E-01	False
Non-Targeting_Control_058	0.860	0.000	0.000	1.860	5.00E-01	False
PRKAB1	11.180	11.910	4.539	0.939	5.00E-01	False
NUP62	34.829	35.380	10.587	0.984	5.00E-01	False
VRK2	16.340	16.987	5.888	0.962	5.02E-01	False
HK3	190.486	194.367	50.092	0.980	5.02E-01	False
RIOK2	5.590	5.773	2.796	0.968	5.02E-01	False
NRBP1	13.760	13.914	5.077	0.989	5.03E-01	False
ERBB4	111.798	113.652	30.085	0.984	5.04E-01	False
BMX	646.276	658.102	164.910	0.982	5.05E-01	False
NEK1	76.538	77.990	21.229	0.981	5.05E-01	False
MAP3K19	10.320	10.824	4.243	0.953	5.06E-01	False
Non-Targeting_Control_038	74.818	75.794	20.683	0.987	5.08E-01	False
EPHA3	853.961	865.210	216.179	0.987	5.11E-01	False
NMRK1	8.170	8.731	3.661	0.936	5.13E-01	False
INSR	192.636	195.022	50.254	0.988	5.13E-01	False
SHPK	62.349	63.351	17.586	0.984	5.14E-01	False
MAPK13	70.088	71.394	19.588	0.982	5.15E-01	False
AK9	2.150	2.658	1.758	0.809	5.16E-01	False
MERTK	6.450	6.682	3.069	0.965	5.16E-01	False
ADCK3	883.631	891.853	222.774	0.991	5.18E-01	False
TBCK	78.688	79.230	21.538	0.993	5.20E-01	False
MOS	6.450	6.653	3.060	0.970	5.20E-01	False

CCL2	985.109	993.185	247.858	0.992	5.21E-01	False
STK35	25.799	25.639	8.119	1.006	5.27E-01	False
PASK	34.399	34.653	10.404	0.993	5.28E-01	False
Non-Targeting_Control_054	782.583	784.837	196.283	0.997	5.28E-01	False
PLXNA3	389.142	390.647	98.697	0.996	5.28E-01	False
PGK2	748.184	750.521	187.788	0.997	5.29E-01	False
CERK	44.289	44.632	12.911	0.992	5.29E-01	False
ADK	2.150	2.598	1.735	0.828	5.30E-01	False
BMP2K	112.658	112.658	29.838	1.000	5.31E-01	False
GRK5	109.648	109.533	29.063	1.001	5.33E-01	False
Non-Targeting_Control_055	144.047	144.520	37.739	0.997	5.33E-01	False
PDIK1L	138.457	138.504	36.248	1.000	5.33E-01	False
PDPK1	3.870	3.571	2.086	1.084	5.34E-01	False
MAST1	178.876	178.354	46.124	1.003	5.34E-01	False
STK10	283.794	283.219	72.098	1.002	5.34E-01	False
SNX16	3.010	3.563	2.083	0.845	5.35E-01	False
FUK	7.740	7.530	3.317	1.028	5.35E-01	False
PDXK	15.050	15.480	5.492	0.972	5.37E-01	False
MAPKAPK5	125.987	125.138	32.934	1.007	5.37E-01	False
IP6K3	3.440	3.543	2.076	0.971	5.39E-01	False
PRKCE	91.158	91.113	24.490	1.000	5.39E-01	False
RYK	4.300	4.519	2.402	0.951	5.41E-01	False
MAST4	8.600	8.465	3.586	1.016	5.42E-01	False
FGFR3	11.180	11.435	4.410	0.978	5.42E-01	False
INSR	9.030	9.452	3.863	0.955	5.42E-01	False
EPHA7	3.870	3.522	2.069	1.099	5.43E-01	False
HKDC1	25.799	25.295	8.031	1.020	5.44E-01	False
GALK2	184.036	183.126	47.307	1.005	5.44E-01	False
PIK3C2G	2.150	2.533	1.711	0.849	5.45E-01	False
NRK	276.484	274.217	69.869	1.008	5.46E-01	False
MORN2	3.440	3.499	2.061	0.983	5.48E-01	False
TGFBR1	200.806	198.522	51.121	1.012	5.48E-01	False
MARK2	121.687	120.275	31.728	1.012	5.48E-01	False
EGFR	73.528	72.521	19.869	1.014	5.53E-01	False
LRGUK	148.777	146.570	38.247	1.015	5.53E-01	False
TPK1	19.780	19.195	6.463	1.030	5.53E-01	False
HKDC1	59.339	58.620	16.407	1.012	5.54E-01	False
MAP4K5	7.740	7.372	3.271	1.050	5.54E-01	False
HK2	839.772	828.025	206.974	1.014	5.55E-01	False
EPHB6	4.730	4.435	2.375	1.067	5.55E-01	False
WNK3	7.740	7.365	3.269	1.051	5.55E-01	False
Non-Targeting_Control_045	38.699	37.876	11.215	1.022	5.55E-01	False
CSNK1G2	446.760	440.171	110.959	1.015	5.55E-01	False
GCK	112.658	110.829	29.384	1.017	5.55E-01	False
IDNK	1.720	1.531	1.294	1.124	5.55E-01	False
PRKD1	2.580	2.489	1.694	1.036	5.56E-01	False
CDKL4	83.418	82.154	22.264	1.015	5.57E-01	False
IRAK2	168.986	165.858	43.028	1.019	5.57E-01	False
ULK4	78.688	77.141	21.018	1.020	5.59E-01	False
STK32C	122.547	120.407	31.760	1.018	5.59E-01	False
MAPK3	206.825	203.034	52.239	1.019	5.59E-01	False
PIK3CG	536.628	527.607	132.605	1.017	5.59E-01	False
PIM2	3.870	3.425	2.036	1.130	5.62E-01	False
MAPK9	24.939	23.947	7.687	1.041	5.62E-01	False
MAP3K15	12.040	12.139	4.601	0.992	5.67E-01	False
GAK	1.290	1.494	1.277	0.864	5.67E-01	False
STK19	101.908	99.115	26.477	1.028	5.68E-01	False
AKT2	433.431	423.597	106.855	1.023	5.69E-01	False
PAK1	6.450	6.280	2.949	1.027	5.70E-01	False
MAGI1	18.060	17.947	6.139	1.006	5.70E-01	False
MAGI1	5.590	5.309	2.652	1.053	5.70E-01	False
UCKL1	30.099	29.614	9.129	1.016	5.72E-01	False
IP6K1	79.978	77.402	21.083	1.033	5.72E-01	False
LMTK2	3.440	3.368	2.016	1.021	5.73E-01	False
PHKA2	95.028	92.802	24.910	1.024	5.75E-01	False
CDK7	0.000	0.559	0.760	0.642	5.77E-01	False
EPHA2	0.000	0.559	0.760	0.642	5.77E-01	False
MET	0.000	0.559	0.760	0.642	5.77E-01	False
BRD3	0.000	0.559	0.760	0.642	5.77E-01	False
HSPB8	0.000	0.559	0.760	0.642	5.77E-01	False
WNK2	0.000	0.559	0.760	0.642	5.77E-01	False
PIP5K1B	0.430	0.559	0.760	0.770	5.77E-01	False
ZAK	262.724	254.944	65.096	1.031	5.78E-01	False

RPS6KA6	227.895	220.808	56.641	1.032	5.79E-01	False
NME4	59.769	57.511	16.130	1.039	5.81E-01	False
TNK2	18.920	17.766	6.091	1.065	5.82E-01	False
IRAK4	459.660	445.330	112.236	1.032	5.82E-01	False
AK2	10.750	10.030	4.024	1.072	5.82E-01	False
MORN2	319.913	309.308	78.558	1.034	5.83E-01	False
Non-Targeting_Control_041	12.470	11.941	4.547	1.044	5.84E-01	False
NTRK2	55.039	53.456	15.118	1.030	5.86E-01	False
IPPK	1349.311	1304.619	324.951	1.034	5.87E-01	False
ADCK1	694.865	671.095	168.126	1.035	5.87E-01	False
FGFR3	295.834	285.311	72.616	1.037	5.88E-01	False
ROR1	8.170	8.049	3.467	1.015	5.89E-01	False
DTYMK	22.360	21.437	7.042	1.043	5.93E-01	False
SBK2	61.489	58.955	16.490	1.043	5.93E-01	False
SCYL3	81.268	78.181	21.277	1.039	5.94E-01	False
PDGFRB	2.150	2.332	1.632	0.922	5.94E-01	False
PDGFRB	92.018	88.743	23.901	1.037	5.94E-01	False
CKM	0.000	0.528	0.738	0.654	5.95E-01	False
SLK	0.000	0.528	0.738	0.654	5.95E-01	False
SNRK	0.000	0.528	0.738	0.654	5.95E-01	False
NUP62	0.430	0.528	0.738	0.814	5.95E-01	False
CDK5R1	718.944	690.361	172.896	1.041	5.96E-01	False
TXK	110.938	105.799	28.136	1.049	5.98E-01	False
TAOK1	2941.996	2821.409	700.410	1.043	5.99E-01	False
ITPKB	88.148	84.434	22.831	1.044	6.02E-01	False
NPR1	275.624	263.410	67.192	1.046	6.02E-01	False
TESK2	19.780	18.387	6.253	1.076	6.03E-01	False
SGK494	15.910	14.559	5.249	1.093	6.04E-01	False
PDK4	132.437	126.286	33.219	1.049	6.05E-01	False
IDNK	2.150	2.284	1.614	0.941	6.05E-01	False
PAK7	187.906	178.782	46.230	1.051	6.06E-01	False
MAP2K4	649.716	619.797	155.427	1.048	6.07E-01	False
MAP2K2	2.580	2.278	1.611	1.133	6.07E-01	False
PFKFB3	10.750	9.779	3.954	1.099	6.07E-01	False
EIF2AK2	2.150	2.274	1.610	0.945	6.08E-01	False
ADRBK1	0.000	0.505	0.721	0.665	6.08E-01	False
CSNK1A1	0.000	0.505	0.721	0.665	6.08E-01	False
NME2	0.000	0.505	0.721	0.665	6.08E-01	False
PI4KA	0.000	0.505	0.721	0.665	6.08E-01	False
PLAU	0.000	0.505	0.721	0.665	6.08E-01	False
RPS6KA1	0.000	0.505	0.721	0.665	6.08E-01	False
RPS6KA3	0.000	0.505	0.721	0.665	6.08E-01	False
TBRG4	0.000	0.505	0.721	0.665	6.08E-01	False
BCKDK	0.000	0.505	0.721	0.665	6.08E-01	False
TLK2	0.000	0.505	0.721	0.665	6.08E-01	False
CDC42BPG	0.000	0.505	0.721	0.665	6.08E-01	False
TRIB3	0.000	0.505	0.721	0.665	6.08E-01	False
STK40	0.000	0.505	0.721	0.665	6.08E-01	False
BRSK1	0.000	0.505	0.721	0.665	6.08E-01	False
STK32C	0.000	0.505	0.721	0.665	6.08E-01	False
EPHA2	0.430	0.505	0.721	0.852	6.08E-01	False
MAGI3	1.720	1.366	1.217	1.259	6.09E-01	False
PKN3	0.000	0.501	0.719	0.666	6.10E-01	False
FASTKD5	0.000	0.501	0.719	0.666	6.10E-01	False
NOL9	0.000	0.501	0.719	0.666	6.10E-01	False
SBK3	3485.504	3318.780	823.526	1.050	6.11E-01	False
MPP1	135.877	128.545	33.779	1.057	6.12E-01	False
CAMK2D	0.000	0.498	0.716	0.668	6.12E-01	False
NEK1	0.000	0.498	0.716	0.668	6.12E-01	False
BRD2	0.000	0.498	0.716	0.668	6.12E-01	False
LRPPRC	0.000	0.498	0.716	0.668	6.12E-01	False
SMG1	0.000	0.498	0.716	0.668	6.12E-01	False
DSTYK	0.000	0.498	0.716	0.668	6.12E-01	False
PANK1	0.000	0.498	0.716	0.668	6.12E-01	False
CIB4	0.000	0.498	0.716	0.668	6.12E-01	False
GRK6	0.430	0.498	0.716	0.864	6.12E-01	False
PIM1	0.430	0.498	0.716	0.864	6.12E-01	False
SGK494	3.870	3.149	1.938	1.229	6.17E-01	False
SRC	7.740	6.860	3.121	1.128	6.18E-01	False
PIP4K2A	70.518	66.371	18.338	1.062	6.19E-01	False
Non-Targeting_Control_016	37.839	35.128	10.523	1.077	6.20E-01	False
EPHB2	40.419	37.809	11.198	1.069	6.25E-01	False
OXSR1	50.739	47.195	13.553	1.075	6.26E-01	False

MPP5	9.460	8.644	3.636	1.094	6.27E-01	False
RPS6KA5	53.749	49.976	14.248	1.075	6.28E-01	False
KIAA1804	9.030	8.596	3.623	1.050	6.32E-01	False
Non-Targeting_Control_040	22.360	20.653	6.840	1.083	6.37E-01	False
PIP5K1C	26.229	24.381	7.798	1.076	6.37E-01	False
ADPGK	83.418	77.575	21.126	1.075	6.39E-01	False
CLK2	1.290	1.275	1.172	1.012	6.39E-01	False
SRPK2	336.253	313.716	79.650	1.072	6.41E-01	False
PFKM	436.441	406.856	102.711	1.073	6.42E-01	False
TEK	1155.815	1077.593	268.753	1.073	6.43E-01	False
TSSK4	202.956	188.400	48.613	1.077	6.43E-01	False
RIPK3	15.480	14.046	5.112	1.102	6.43E-01	False
PRKAA2	3.010	3.021	1.892	0.996	6.43E-01	False
RAF1	5.160	4.826	2.500	1.069	6.44E-01	False
DCK	1.720	1.262	1.166	1.363	6.44E-01	False
PRKAR1B	5.160	4.814	2.497	1.072	6.46E-01	False
TBCK	2.580	2.117	1.546	1.219	6.47E-01	False
STRADB	1.290	1.251	1.161	1.031	6.48E-01	False
MUSK	40265.614	37442.406	9270.267	1.075	6.48E-01	False
SRM	13232.962	12299.315	3046.511	1.076	6.48E-01	False
MAP3K3	1.290	1.245	1.157	1.036	6.50E-01	False
CLP1	633.806	587.305	147.384	1.079	6.51E-01	False
PHKG2	11.180	10.252	4.086	1.091	6.51E-01	False
TWF1	4.300	3.873	2.189	1.110	6.52E-01	False
TNIK	29.239	26.842	8.425	1.089	6.52E-01	False
ITK	421.821	389.915	98.516	1.082	6.53E-01	False
GRK4	96.318	88.785	23.912	1.085	6.54E-01	False
TAB1	9.890	8.391	3.564	1.179	6.54E-01	False
VRK2	7.310	6.555	3.031	1.115	6.56E-01	False
MET	187.476	172.811	44.751	1.085	6.56E-01	False
PGK1	7.740	6.548	3.029	1.182	6.57E-01	False
PHKA1	277.774	255.361	65.199	1.088	6.60E-01	False
PRKAB2	440.310	405.708	102.426	1.085	6.60E-01	False
MST1R	5.160	4.704	2.462	1.097	6.63E-01	False
DYRK4	7.310	6.500	3.015	1.125	6.63E-01	False
ROS1	145.767	133.329	34.965	1.093	6.63E-01	False
BAZ1B	781.293	718.585	179.882	1.087	6.64E-01	False
MORN1	9.030	8.293	3.537	1.089	6.65E-01	False
PTK2B	447.620	410.840	103.697	1.090	6.65E-01	False
STK38L	157.377	144.031	37.618	1.093	6.66E-01	False
KHK	8.170	7.364	3.269	1.109	6.67E-01	False
AK9	2.150	2.035	1.513	1.056	6.68E-01	False
TNIK	18.490	16.428	5.741	1.126	6.69E-01	False
Non-Targeting_Control_093	2.150	2.022	1.507	1.063	6.71E-01	False
GUCY2F	1017.788	930.820	232.420	1.093	6.72E-01	False
CSNK2B	2.150	2.019	1.506	1.065	6.72E-01	False
KSR1	2.150	1.998	1.498	1.076	6.77E-01	False
WNK3	42.999	38.106	11.273	1.128	6.78E-01	False
CAMK2G	51.169	46.186	13.300	1.108	6.81E-01	False
CAMKV	46.439	41.519	12.130	1.119	6.85E-01	False
LRRK2	1213.004	1100.004	274.300	1.103	6.85E-01	False
CDC42BPB	313.033	283.450	72.155	1.104	6.86E-01	False
CSNK1G1	15.910	13.459	4.956	1.182	6.87E-01	False
PRKAA2	64.929	57.708	16.179	1.125	6.87E-01	False
SIK1	341.843	308.168	78.276	1.109	6.89E-01	False
NME3	10.750	8.939	3.720	1.203	6.91E-01	False
DCLK2	3.010	2.786	1.806	1.081	6.92E-01	False
UCK1	792.473	714.675	178.915	1.109	6.92E-01	False
MAK	1218.163	1098.624	273.959	1.109	6.93E-01	False
EPHB6	118.247	106.146	28.223	1.114	6.93E-01	False
TSSK4	37.839	33.126	10.018	1.142	6.93E-01	False
PIK3R3	23.219	20.532	6.809	1.131	6.94E-01	False
CDKL5	64.069	57.422	16.108	1.116	6.94E-01	False
RPS6KA6	12.900	10.679	4.203	1.208	6.94E-01	False
PBK	10.750	8.880	3.703	1.211	6.96E-01	False
FLT3	13.760	11.535	4.437	1.193	6.97E-01	False
MPP3	132.007	118.366	31.254	1.115	6.98E-01	False
AKT1	27.519	24.020	7.705	1.146	6.98E-01	False
MAST3	933.940	837.341	209.280	1.115	7.00E-01	False
AATK	3.870	2.745	1.791	1.410	7.00E-01	False
RFK	27.089	23.946	7.686	1.131	7.02E-01	False
MYLK3	11.610	9.661	3.922	1.202	7.06E-01	False
NEK4	147.057	131.053	34.401	1.122	7.06E-01	False

STK17A	164.256	146.162	38.147	1.124	7.07E-01	False
CAMK1	273.474	243.524	62.268	1.123	7.07E-01	False
TIE1	2026.546	1809.147	449.840	1.120	7.08E-01	False
MAP3K10	32.679	28.248	8.783	1.157	7.09E-01	False
DGKQ	69.658	61.151	17.038	1.139	7.10E-01	False
CKMT1B	27.089	23.764	7.640	1.140	7.10E-01	False
LATS2	6.450	5.246	2.633	1.230	7.12E-01	False
FUK	13.330	11.350	4.387	1.174	7.12E-01	False
CLK4	558.558	496.361	124.870	1.125	7.12E-01	False
BCR	80.838	70.785	19.437	1.142	7.13E-01	False
ATM	4.730	3.524	2.070	1.342	7.13E-01	False
LATS2	677.665	601.357	150.862	1.127	7.15E-01	False
SBK3	9.460	7.808	3.398	1.211	7.16E-01	False
TEK	28.809	24.495	7.827	1.176	7.17E-01	False
BMP2K	39.559	34.194	10.288	1.157	7.18E-01	False
PRKAG1	525.879	464.814	117.059	1.131	7.19E-01	False
BRSK2	54.179	47.421	13.609	1.142	7.20E-01	False
GK	196.506	172.883	44.769	1.137	7.22E-01	False
STK17B	6.020	5.161	2.606	1.166	7.23E-01	False
MPP4	534.048	471.282	118.661	1.133	7.23E-01	False
MAP3K8	76.538	66.650	18.407	1.148	7.24E-01	False
MPP3	81.268	71.034	19.499	1.144	7.25E-01	False
PIK3C2B	27.089	23.392	7.545	1.158	7.28E-01	False
WNK1	345.282	303.272	77.063	1.139	7.28E-01	False
GUK1	11.180	9.415	3.853	1.187	7.29E-01	False
MAP3K1	105.778	91.907	24.687	1.151	7.29E-01	False
PIP5K1C	12.040	10.272	4.091	1.172	7.29E-01	False
GALK2	153.077	134.046	35.143	1.142	7.30E-01	False
MAP3K4	2.150	1.787	1.408	1.203	7.32E-01	False
SIK2	1136.895	997.107	248.829	1.140	7.32E-01	False
DYRK4	45.149	39.014	11.501	1.157	7.33E-01	False
PRKACG	10.750	8.500	3.595	1.265	7.33E-01	False
PAK1	203.386	177.408	45.890	1.146	7.34E-01	False
MYO3B	328.513	286.821	72.990	1.145	7.35E-01	False
SYK	39.559	33.679	10.158	1.175	7.36E-01	False
CSNK1A1L	1.290	0.999	1.030	1.291	7.36E-01	False
SPEG	67.939	58.114	16.280	1.169	7.37E-01	False
GRK6	3.870	2.567	1.724	1.507	7.37E-01	False
TWF1	1.290	0.996	1.028	1.296	7.37E-01	False
SRM	1.290	0.996	1.028	1.296	7.37E-01	False
CDK11A	3.010	2.560	1.721	1.176	7.38E-01	False
HK1	116.957	100.721	26.876	1.161	7.40E-01	False
CLK1	23.219	19.558	6.557	1.187	7.45E-01	False
RPS6KA5	332.813	287.847	73.244	1.156	7.46E-01	False
ETNK1	902.550	782.901	195.804	1.153	7.47E-01	False
ERBB2	4.300	3.324	2.000	1.294	7.48E-01	False
TPK1	213.705	184.036	47.532	1.161	7.49E-01	False
FGFR3	1.290	0.000	0.000	2.290	7.50E-01	False
NEK2	1.290	0.000	0.000	2.290	7.50E-01	False
PI4KA	1.290	0.000	0.000	2.290	7.50E-01	False
PRKDC	1.290	0.000	0.000	2.290	7.50E-01	False
CCL2	1.290	0.000	0.000	2.290	7.50E-01	False
TTN	1.290	0.000	0.000	2.290	7.50E-01	False
DOLK	1.290	0.000	0.000	2.290	7.50E-01	False
TRIM33	1.290	0.000	0.000	2.290	7.50E-01	False
CHUK	1.720	0.000	0.000	2.720	7.50E-01	False
ERN1	1.720	0.000	0.000	2.720	7.50E-01	False
PIK3C2A	1.720	0.000	0.000	2.720	7.50E-01	False
MAPK10	1.720	0.000	0.000	2.720	7.50E-01	False
FASTKD2	1.720	0.000	0.000	2.720	7.50E-01	False
SEPHS2	1.720	0.000	0.000	2.720	7.50E-01	False
MELK	4.730	3.307	1.994	1.430	7.51E-01	False
PRKAR1A	84.708	72.140	19.774	1.174	7.51E-01	False
EPHA5	5.590	4.122	2.272	1.356	7.52E-01	False
DGKH	4.730	3.298	1.991	1.434	7.52E-01	False
NUAK2	130.287	111.758	29.615	1.166	7.53E-01	False
STK32C	445.040	383.827	97.009	1.159	7.54E-01	False
PPIP5K2	67.079	57.199	16.052	1.173	7.56E-01	False
PIM3	4.730	3.267	1.980	1.448	7.58E-01	False
DYRK1B	67.509	57.096	16.026	1.182	7.58E-01	False
FES	0.000	0.279	0.533	0.782	7.58E-01	False
PRKACG	0.000	0.279	0.533	0.782	7.58E-01	False
MAPK13	0.000	0.279	0.533	0.782	7.58E-01	False

MAP2K5	0.000	0.279	0.533	0.782	7.58E-01	False
TGFBR2	0.000	0.279	0.533	0.782	7.58E-01	False
CDK10	0.000	0.279	0.533	0.782	7.58E-01	False
RIPK1	0.000	0.279	0.533	0.782	7.58E-01	False
RPS6KA4	0.000	0.279	0.533	0.782	7.58E-01	False
GNE	0.000	0.279	0.533	0.782	7.58E-01	False
ACTR2	0.000	0.279	0.533	0.782	7.58E-01	False
AAK1	0.000	0.279	0.533	0.782	7.58E-01	False
LMTK2	0.000	0.279	0.533	0.782	7.58E-01	False
SEPHS2	0.000	0.279	0.533	0.782	7.58E-01	False
SCYL1	0.000	0.279	0.533	0.782	7.58E-01	False
FLT1	0.860	0.279	0.533	3.078	7.58E-01	False
TK1	0.860	0.279	0.533	3.078	7.58E-01	False
SPEG	47.729	39.831	11.706	1.198	7.60E-01	False
CD2	1213.434	1039.869	259.414	1.167	7.63E-01	False
NME5	4643.039	3981.077	987.468	1.166	7.64E-01	False
MLKL	66.649	55.946	15.739	1.191	7.64E-01	False
CDK12	28.809	23.441	7.557	1.229	7.64E-01	False
CDK10	4907.913	4203.310	1042.478	1.168	7.65E-01	False
TAB1	66.219	55.791	15.701	1.187	7.67E-01	False
STYK1	2.580	1.646	1.346	1.568	7.67E-01	False
STK40	7.310	5.632	2.753	1.298	7.70E-01	False
TAOK3	7.740	5.632	2.752	1.374	7.70E-01	False
EPHB1	308.733	262.099	66.868	1.178	7.70E-01	False
GSK3B	13.760	10.561	4.171	1.303	7.75E-01	False
CLK2	8.600	6.392	2.983	1.345	7.77E-01	False
RPS6KA4	61.489	51.081	14.524	1.204	7.77E-01	False
NIM1K	539.208	456.655	115.040	1.181	7.77E-01	False
CSNK2A2	29.239	23.971	7.693	1.220	7.77E-01	False
TTK	12.040	9.691	3.930	1.242	7.77E-01	False
ABL1	13.760	10.521	4.160	1.308	7.78E-01	False
AXL	0.000	0.252	0.506	0.799	7.78E-01	False
BLK	0.000	0.252	0.506	0.799	7.78E-01	False
CDK11B	0.000	0.252	0.506	0.799	7.78E-01	False
CDK3	0.000	0.252	0.506	0.799	7.78E-01	False
CKS1B	0.000	0.252	0.506	0.799	7.78E-01	False
CSNK2A1	0.000	0.252	0.506	0.799	7.78E-01	False
PDPK1	0.000	0.252	0.506	0.799	7.78E-01	False
RET	0.000	0.252	0.506	0.799	7.78E-01	False
MAP2K4	0.000	0.252	0.506	0.799	7.78E-01	False
RIPK1	0.000	0.252	0.506	0.799	7.78E-01	False
RPS6KA4	0.000	0.252	0.506	0.799	7.78E-01	False
LMTK2	0.000	0.252	0.506	0.799	7.78E-01	False
SRPK3	0.000	0.252	0.506	0.799	7.78E-01	False
NAGK	0.000	0.252	0.506	0.799	7.78E-01	False
RIOK2	0.000	0.252	0.506	0.799	7.78E-01	False
NEK11	0.000	0.252	0.506	0.799	7.78E-01	False
XRCC6BP1	0.000	0.252	0.506	0.799	7.78E-01	False
TP53RK	0.000	0.252	0.506	0.799	7.78E-01	False
DGKI	0.430	0.252	0.506	1.704	7.78E-01	False
PLK2	0.430	0.252	0.506	1.704	7.78E-01	False
CSNK1G1	0.430	0.252	0.506	1.704	7.78E-01	False
DGKE	0.860	0.252	0.506	3.408	7.78E-01	False
AMHR2	3.870	2.352	1.640	1.645	7.80E-01	False
CDK9	0.000	0.249	0.503	0.801	7.81E-01	False
ERBB2	0.000	0.249	0.503	0.801	7.81E-01	False
FRK	0.000	0.249	0.503	0.801	7.81E-01	False
MKNK2	0.000	0.249	0.503	0.801	7.81E-01	False
HUS1	0.000	0.249	0.503	0.801	7.81E-01	False
MVK	0.000	0.249	0.503	0.801	7.81E-01	False
PIK3C2G	0.000	0.249	0.503	0.801	7.81E-01	False
PRKAA1	0.000	0.249	0.503	0.801	7.81E-01	False
PRKDC	0.000	0.249	0.503	0.801	7.81E-01	False
MOK	0.000	0.249	0.503	0.801	7.81E-01	False
RYK	0.000	0.249	0.503	0.801	7.81E-01	False
AURKC	0.000	0.249	0.503	0.801	7.81E-01	False
TEC	0.000	0.249	0.503	0.801	7.81E-01	False
TNK1	0.000	0.249	0.503	0.801	7.81E-01	False
TSSK6	0.000	0.249	0.503	0.801	7.81E-01	False
STRADA	0.000	0.249	0.503	0.801	7.81E-01	False
LMTK3	0.000	0.249	0.503	0.801	7.81E-01	False
AK7	0.000	0.249	0.503	0.801	7.81E-01	False
MAPK15	0.000	0.249	0.503	0.801	7.81E-01	False

PAN3	0.000	0.249	0.503	0.801	7.81E-01	False
NOL9	0.430	0.249	0.503	1.727	7.81E-01	False
EXOSC10	0.860	0.249	0.503	3.455	7.81E-01	False
TIE1	0.860	0.249	0.503	3.455	7.81E-01	False
CHEK2	57.619	47.440	13.614	1.215	7.82E-01	False
DMPK	259.284	217.988	55.943	1.189	7.83E-01	False
PRKAG3	12.900	9.621	3.911	1.341	7.83E-01	False
PFKFB1	543.078	457.525	115.255	1.187	7.84E-01	False
PRKCQ	57.619	47.352	13.592	1.217	7.84E-01	False
IGF1R	67.079	55.657	15.667	1.205	7.87E-01	False
Non-Targeting_Control_002	83.418	69.059	19.007	1.208	7.88E-01	False
FASTKD1	7.310	5.476	2.704	1.335	7.88E-01	False
PAK4	14.190	11.210	4.349	1.266	7.88E-01	False
JAK2	13.330	10.374	4.119	1.285	7.89E-01	False
LMTK3	793.763	664.960	166.608	1.194	7.91E-01	False
PRPS1L1	7.740	5.448	2.696	1.421	7.91E-01	False
PSKH2	28.379	22.747	7.379	1.248	7.93E-01	False
LRGUK	768.823	642.588	161.069	1.196	7.93E-01	False
CDKL4	1.290	0.838	0.939	1.539	7.94E-01	False
MAPK15	9.890	7.025	3.170	1.408	7.95E-01	False
PIK3R1	3.010	2.271	1.608	1.325	7.96E-01	False
TYRO3	39.559	31.805	9.684	1.244	7.97E-01	False
FGFRL1	39.989	31.802	9.683	1.257	7.97E-01	False
AXL	979.089	816.320	204.077	1.199	7.98E-01	False
GSK3B	2185.642	1822.128	453.053	1.200	7.98E-01	False
NEK7	96.318	79.195	21.529	1.216	7.99E-01	False
MAP3K11	210.695	174.158	45.085	1.210	7.99E-01	False
LATS1	2.150	1.514	1.286	1.420	8.00E-01	False
Non-Targeting_Control_075	2.580	1.514	1.286	1.704	8.00E-01	False
CDK5	45.149	36.673	10.912	1.231	8.00E-01	False
AKT1	3.870	2.251	1.600	1.720	8.00E-01	False
NME2	174.146	143.964	37.602	1.210	8.01E-01	False
PCK1	8.170	6.160	2.913	1.326	8.01E-01	False
Non-Targeting_Control_056	9.030	6.941	3.145	1.301	8.03E-01	False
RYK	11.180	8.553	3.611	1.307	8.03E-01	False
ADPGK	7.310	5.316	2.655	1.375	8.06E-01	False
AK3	353.022	291.603	74.174	1.211	8.06E-01	False
TAOK1	15.910	11.749	4.495	1.354	8.07E-01	False
TRPM7	47.299	38.058	11.261	1.243	8.07E-01	False
FXN	37.409	29.797	9.176	1.255	8.08E-01	False
TBCK	34.829	27.318	8.547	1.275	8.08E-01	False
MARK3	455.360	374.959	94.813	1.214	8.10E-01	False
EPHA2	1090.886	898.681	224.464	1.214	8.11E-01	False
CHKB	141.897	115.374	30.512	1.230	8.12E-01	False
DGKA	337.973	277.034	70.566	1.220	8.12E-01	False
MAST4	98.038	79.886	21.701	1.227	8.12E-01	False
PHKB	1127.435	927.402	231.574	1.216	8.13E-01	False
VRK1	76.968	61.577	17.144	1.250	8.15E-01	False
PRKAG3	1.290	0.777	0.902	1.660	8.15E-01	False
PIP4K2A	1681.263	1379.089	343.384	1.219	8.17E-01	False
ROR1	26.229	20.457	6.790	1.282	8.20E-01	False
PKLR	206.825	167.716	43.488	1.233	8.20E-01	False
EIF2AK4	75.248	60.458	16.865	1.245	8.20E-01	False
PGM2L1	32.679	25.321	8.038	1.291	8.20E-01	False
PRKAA1	754.634	616.304	154.563	1.224	8.20E-01	False
PHKA2	674.225	550.287	138.220	1.225	8.21E-01	False
FN3K	20.210	15.526	5.504	1.302	8.23E-01	False
CALM3	1.290	0.754	0.888	1.712	8.23E-01	False
TP53RK	1.290	0.754	0.888	1.712	8.23E-01	False
NEK5	1.290	0.750	0.886	1.720	8.25E-01	False
SRPK3	21.500	16.278	5.702	1.321	8.26E-01	False
PI4KB	1.720	0.747	0.884	2.303	8.26E-01	False
IRAK2	6.880	4.358	2.350	1.579	8.27E-01	False
MAP4K4	24.509	18.669	6.327	1.313	8.27E-01	False
Non-Targeting_Control_082	15.480	11.439	4.411	1.353	8.27E-01	False
POMK	17.630	13.015	4.837	1.355	8.28E-01	False
FES	87.718	69.613	19.145	1.260	8.29E-01	False
PPIP5K1	64.499	50.939	14.489	1.266	8.30E-01	False
PIK3R1	4.730	2.823	1.820	1.676	8.30E-01	False
IP6K2	985.969	796.839	199.254	1.237	8.32E-01	False
PTK7	200.376	160.887	41.796	1.245	8.32E-01	False
HIPK1	97.178	77.492	21.105	1.254	8.32E-01	False
PIK3CA	26.229	20.133	6.706	1.303	8.33E-01	False

RPS6KA3	229.615	183.930	47.506	1.248	8.35E-01	False
PRKAR1A	51.169	40.188	11.796	1.273	8.35E-01	False
NIM1K	623.916	501.891	126.239	1.243	8.36E-01	False
TRPM6	6.020	4.273	2.322	1.409	8.37E-01	False
EPHA4	5.590	3.520	2.069	1.588	8.37E-01	False
ERBB4	37.409	28.851	8.936	1.297	8.37E-01	False
TTBK1	676.805	544.030	136.671	1.244	8.37E-01	False
AK9	8.600	5.798	2.803	1.483	8.37E-01	False
TGFBR2	15.050	11.268	4.364	1.336	8.38E-01	False
PKD1	68.799	53.732	15.187	1.280	8.38E-01	False
MAST2	245.955	196.206	50.547	1.254	8.38E-01	False
PRKX	3.010	2.046	1.517	1.471	8.39E-01	False
IP6K3	22.360	16.763	5.829	1.334	8.40E-01	False
AATK	2.580	1.343	1.205	1.921	8.41E-01	False
NME2	31.389	23.907	7.676	1.313	8.41E-01	False
MAP3K5	24.509	18.320	6.236	1.338	8.42E-01	False
SBK2	26.229	19.896	6.645	1.318	8.42E-01	False
TWF2	579.197	463.201	116.660	1.250	8.43E-01	False
ULK1	7.310	4.974	2.548	1.470	8.43E-01	False
NEK9	20.210	15.121	5.397	1.336	8.43E-01	False
NTRK3	1062.937	850.239	212.473	1.250	8.43E-01	False
NME1	23.219	17.459	6.011	1.330	8.45E-01	False
ADCK4	27.519	20.627	6.834	1.334	8.45E-01	False
CAMKV	30.529	22.999	7.444	1.327	8.45E-01	False
DGKA	161.676	127.535	33.528	1.268	8.46E-01	False
EPHA7	2583.384	2063.540	512.811	1.252	8.46E-01	False
PRKCZ	31.819	23.752	7.637	1.340	8.47E-01	False
AKT3	6.450	4.169	2.288	1.547	8.49E-01	False
CAMK1D	10.750	7.197	3.220	1.494	8.49E-01	False
WNK2	91.158	71.250	19.553	1.279	8.51E-01	False
GCK	15.480	11.032	4.300	1.403	8.52E-01	False
SBK1	202.096	159.115	41.357	1.270	8.53E-01	False
PDXK	84.708	65.442	18.107	1.294	8.54E-01	False
PIP4K2C	10.750	7.133	3.202	1.507	8.54E-01	False
PRPF4B	6.880	4.115	2.270	1.672	8.55E-01	False
BMP2K	15.480	10.967	4.282	1.412	8.56E-01	False
BMPR1B	117.817	91.422	24.567	1.289	8.56E-01	False
MINK1	99.758	77.143	21.018	1.293	8.56E-01	False
FASTKD2	19.350	14.030	5.108	1.379	8.58E-01	False
NTRK3	43.859	32.828	9.943	1.336	8.58E-01	False
Non-Targeting_Control_035	24.509	17.907	6.128	1.369	8.59E-01	False
ANKK1	21.070	15.562	5.514	1.354	8.59E-01	False
CKMT2	69.658	53.125	15.035	1.311	8.61E-01	False
CAMK1	282.504	220.799	56.639	1.279	8.61E-01	False
AK7	4.730	2.611	1.740	1.812	8.62E-01	False
SIK2	20.640	14.716	5.290	1.403	8.62E-01	False
CDK20	25.369	18.576	6.302	1.366	8.63E-01	False
Non-Targeting_Control_020	91.158	70.179	19.286	1.299	8.64E-01	False
GUCY2D	23.649	16.972	5.884	1.393	8.65E-01	False
NAGK	116.097	89.677	24.134	1.295	8.65E-01	False
PFKFB2	17.630	12.326	4.651	1.430	8.66E-01	False
HK3	10.320	6.975	3.155	1.480	8.67E-01	False
PAK6	202.096	156.762	40.774	1.289	8.67E-01	False
STK17A	2875.347	2247.017	558.228	1.280	8.68E-01	False
UCK2	42.999	31.634	9.641	1.359	8.68E-01	False
PGK1	45.579	33.965	10.230	1.342	8.68E-01	False
HUS1	5.160	3.267	1.980	1.580	8.69E-01	False
NRBP2	3.010	1.874	1.446	1.606	8.69E-01	False
ABL2	37.409	27.612	8.621	1.355	8.72E-01	False
MAP4K4	48.159	36.116	10.772	1.333	8.72E-01	False
RIPK2	415.371	321.566	81.593	1.292	8.72E-01	False
AKT2	18.490	12.947	4.819	1.428	8.73E-01	False
PIK3C2G	88.148	67.109	18.522	1.314	8.73E-01	False
LAMTOR3	391.291	302.660	76.912	1.293	8.73E-01	False
SRPK1	149.637	114.495	30.294	1.307	8.73E-01	False
PAK2	7.740	4.660	2.448	1.661	8.74E-01	False
ACVR1	28.379	20.562	6.817	1.380	8.75E-01	False
CSNK1E	2.150	0.000	0.000	3.150	8.75E-01	False
MPP2	2.150	0.000	0.000	3.150	8.75E-01	False
CIT	2.150	0.000	0.000	3.150	8.75E-01	False
PXK	2.150	0.000	0.000	3.150	8.75E-01	False
STK31	2.150	0.000	0.000	3.150	8.75E-01	False
FYN	2.580	0.000	0.000	3.580	8.75E-01	False

AK3	2.580	0.000	0.000	3.580	8.75E-01	False
RBKS	2.580	0.000	0.000	3.580	8.75E-01	False
Non-Targeting_Control_073	19.780	13.656	5.008	1.448	8.75E-01	False
ALK	3.010	1.834	1.428	1.641	8.76E-01	False
NTRK2	2003.756	1549.672	385.610	1.293	8.77E-01	False
DGKD	15.480	10.564	4.172	1.465	8.78E-01	False
LRGUK	13.760	9.051	3.751	1.520	8.78E-01	False
PKDCC	472.560	363.436	91.960	1.300	8.78E-01	False
HKDC1	15.050	10.554	4.169	1.426	8.79E-01	False
PRKCI	6.020	3.893	2.196	1.546	8.79E-01	False
CDKL3	21.070	15.038	5.375	1.401	8.81E-01	False
NIM1K	167.696	127.264	33.461	1.318	8.81E-01	False
FGFR2	44.289	32.588	9.882	1.359	8.82E-01	False
PFKP	163.826	123.999	32.651	1.321	8.82E-01	False
PKD1	410.211	313.899	79.695	1.307	8.83E-01	False
NADK	236.925	179.957	46.521	1.317	8.83E-01	False
DLG3	1286.102	985.773	246.024	1.305	8.85E-01	False
TPK1	56.329	41.630	12.158	1.353	8.85E-01	False
CDK2	3.870	1.780	1.405	2.174	8.85E-01	False
AMHR2	247.675	188.024	48.520	1.317	8.85E-01	False
PIK3R5	6.880	3.829	2.174	1.797	8.85E-01	False
AURKC	11.610	7.449	3.294	1.559	8.86E-01	False
NMRK1	33.969	24.028	7.707	1.414	8.86E-01	False
AATK	42.999	30.846	9.442	1.394	8.86E-01	False
TGFBR1	82.128	61.374	17.093	1.338	8.87E-01	False
PIK3CB	16.770	11.137	4.329	1.506	8.87E-01	False
ICK	4.730	2.423	1.668	1.952	8.88E-01	False
ACVR1C	8.170	5.229	2.628	1.562	8.88E-01	False
ICK	8.600	5.226	2.627	1.646	8.88E-01	False
TNK1	32.679	23.147	7.482	1.412	8.89E-01	False
NUP62	1.290	0.559	0.760	2.309	8.89E-01	False
NEK10	1.290	0.559	0.760	2.309	8.89E-01	False
MST1	32.679	23.134	7.478	1.413	8.89E-01	False
EIF2AK2	170.706	128.241	33.703	1.331	8.90E-01	False
UCKL1	70.518	51.992	14.752	1.356	8.90E-01	False
PRKAA2	2.580	1.117	1.093	2.309	8.91E-01	False
PRKAR1B	2.580	1.117	1.093	2.309	8.91E-01	False
PANK2	369.792	279.462	71.167	1.323	8.91E-01	False
MERTK	9.890	5.902	2.835	1.676	8.92E-01	False
TSSK6	22.360	15.505	5.499	1.442	8.92E-01	False
HK2	8.600	5.151	2.603	1.670	8.94E-01	False
PPIP5K1	36.979	25.951	8.198	1.425	8.95E-01	False
NME8	550.388	415.557	104.865	1.324	8.95E-01	False
TEK	302.283	227.087	58.196	1.331	8.96E-01	False
IP6K1	17.630	11.651	4.469	1.513	8.98E-01	False
NEK4	1.720	0.532	0.741	3.235	8.98E-01	False
PRKCH	19.780	13.120	4.865	1.508	8.98E-01	False
PLXNA3	1.290	0.528	0.738	2.442	8.99E-01	False
TPR	580.487	435.948	109.913	1.332	8.99E-01	False
Non-Targeting_Control_078	34.829	24.261	7.767	1.436	8.99E-01	False
TSSK4	390.861	292.473	74.389	1.336	8.99E-01	False
PI4KA	74.388	54.273	15.322	1.371	9.00E-01	False
IGFN1	7.740	4.367	2.353	1.772	9.00E-01	False
AXL	54.179	39.174	11.541	1.383	9.01E-01	False
CDK17	385.272	288.023	73.287	1.338	9.01E-01	False
Non-Targeting_Control_019	22.790	15.259	5.434	1.494	9.01E-01	False
ROCK2	405.051	302.697	76.921	1.338	9.02E-01	False
DCLK3	500.509	373.966	94.567	1.338	9.02E-01	False
LRPPRC	5.160	2.961	1.871	1.742	9.04E-01	False
SRPK2	6.880	3.637	2.109	1.891	9.04E-01	False
SPHK1	829.882	619.310	155.307	1.340	9.04E-01	False
PRKCB	17.200	11.494	4.426	1.496	9.05E-01	False
N4BP2	92.018	67.258	18.559	1.368	9.05E-01	False
PRKACB	9.460	5.698	2.772	1.660	9.06E-01	False
AK8	1.290	0.505	0.721	2.556	9.06E-01	False
AK5	2094.914	1561.219	388.469	1.342	9.07E-01	False
MPP5	4.730	2.264	1.606	2.089	9.08E-01	False
NME7	69.228	49.884	14.225	1.388	9.08E-01	False
MPP5	1.290	0.498	0.716	2.591	9.08E-01	False
SH3BP5	59.769	42.424	12.357	1.409	9.08E-01	False
EPHA4	11.610	7.072	3.184	1.642	9.09E-01	False
DGUOK	12.470	7.781	3.390	1.603	9.09E-01	False
TNNI3K	15.050	9.938	3.999	1.514	9.09E-01	False

GUCY2C	4.730	2.244	1.598	2.108	9.10E-01	False
PRKCD	491.909	363.097	91.876	1.355	9.10E-01	False
TYK2	1931.948	1432.611	356.633	1.349	9.11E-01	False
CSNK1A1L	85.998	61.508	17.127	1.398	9.11E-01	False
CDK3	35.689	24.517	7.832	1.456	9.11E-01	False
MAPK13	59.769	42.238	12.311	1.415	9.11E-01	False
DGKZ	27.089	18.632	6.317	1.454	9.11E-01	False
CAMK2G	7.310	4.236	2.310	1.725	9.11E-01	False
CDK15	1955.167	1449.247	360.751	1.349	9.11E-01	False
PIK3R5	58.049	41.479	12.120	1.399	9.11E-01	False
MPP7	60.199	42.872	12.470	1.404	9.12E-01	False
ERBB2	2.580	1.009	1.035	2.556	9.13E-01	False
TRIB1	2.580	1.009	1.035	2.556	9.13E-01	False
FGFR4	88.578	63.543	17.634	1.394	9.13E-01	False
FRK	10.320	6.291	2.952	1.640	9.13E-01	False
IP6K3	2.580	1.006	1.033	2.565	9.13E-01	False
MET	178.016	129.973	34.133	1.370	9.14E-01	False
EIF2AK2	934.370	688.716	172.488	1.357	9.14E-01	False
CDK16	137.167	99.573	26.591	1.378	9.14E-01	False
Non-Targeting_Control_083	159.527	115.817	30.622	1.377	9.14E-01	False
Non-Targeting_Control_021	8.170	4.876	2.517	1.675	9.15E-01	False
SPHK1	2.150	0.996	1.028	2.159	9.15E-01	False
PFKFB1	2.580	0.996	1.028	2.591	9.15E-01	False
RPS6KL1	18.920	11.955	4.551	1.583	9.15E-01	False
FXN	100.618	72.124	19.770	1.395	9.15E-01	False
MAP3K12	53.749	37.508	11.122	1.433	9.16E-01	False
MINK1	3.440	1.568	1.311	2.194	9.17E-01	False
PHKG1	284.654	207.120	53.251	1.374	9.18E-01	False
RBKS	2299.160	1684.965	419.100	1.365	9.19E-01	False
PRKCQ	1222.463	894.017	223.310	1.367	9.20E-01	False
NME2	47.299	32.824	9.942	1.441	9.20E-01	False
DAPK1	301.853	218.594	56.093	1.381	9.21E-01	False
CAMKK1	294.114	212.852	54.671	1.382	9.22E-01	False
FN3KRP	90.728	63.935	17.731	1.419	9.23E-01	False
DCK	5.160	2.763	1.797	1.867	9.23E-01	False
BMX	2005.046	1459.138	363.200	1.374	9.24E-01	False
SBK2	256.704	184.725	47.703	1.390	9.24E-01	False
EPHB6	8.600	4.736	2.472	1.816	9.24E-01	False
NADK	3.010	1.514	1.286	1.988	9.24E-01	False
MAP2K2	27.949	18.130	6.186	1.542	9.25E-01	False
PIK3C3	94.598	66.625	18.401	1.420	9.25E-01	False
PIP4K2B	196.076	140.741	36.803	1.393	9.25E-01	False
SIK1	71.808	49.914	14.233	1.439	9.25E-01	False
PTK7	43.429	29.595	9.125	1.467	9.26E-01	False
CDK20	23.649	15.218	5.423	1.554	9.26E-01	False
Non-Targeting_Control_003	398.601	287.063	73.050	1.389	9.26E-01	False
CDK16	120.827	85.195	23.020	1.418	9.27E-01	False
PIKFYVE	71.808	49.729	14.187	1.444	9.27E-01	False
CAMK2D	3.440	1.494	1.277	2.303	9.27E-01	False
HUNK	6.880	3.365	2.014	2.045	9.27E-01	False
SIK3	12.040	7.418	3.285	1.623	9.27E-01	False
AGK	263.154	188.279	48.583	1.398	9.29E-01	False
TRIM33	78.258	54.629	15.411	1.433	9.29E-01	False
PPIP5K1	2223.052	1604.335	399.141	1.386	9.29E-01	False
DCK	307.013	219.819	56.396	1.397	9.29E-01	False
PDK4	210.265	149.672	39.016	1.405	9.30E-01	False
CKMT1A	106.638	74.591	20.384	1.430	9.30E-01	False
GCK	26.659	17.164	5.934	1.553	9.31E-01	False
PFKP	103.628	72.332	19.822	1.433	9.31E-01	False
MAP3K9	1937.108	1391.191	346.380	1.392	9.32E-01	False
TRPM6	105.778	73.692	20.160	1.435	9.32E-01	False
NME1	4.300	2.039	1.514	2.109	9.33E-01	False
TRIB2	3829.067	2745.263	681.561	1.395	9.33E-01	False
TAF1L	109.218	76.279	20.804	1.432	9.34E-01	False
IRAK3	22.360	14.203	5.154	1.574	9.35E-01	False
TESK2	49.879	33.306	10.064	1.498	9.35E-01	False
MYO3B	131.577	91.751	24.649	1.434	9.35E-01	False
IRAK1	13.330	7.927	3.432	1.682	9.36E-01	False
PRKAB1	11.180	6.565	3.034	1.703	9.36E-01	False
CPNE3	545.228	387.452	97.907	1.407	9.36E-01	False
MORN2	479.440	339.881	86.128	1.411	9.36E-01	False
TAOK3	11.180	6.548	3.029	1.707	9.36E-01	False
AURKC	8.170	4.535	2.407	1.802	9.37E-01	False

SPHK2	3.010	0.000	0.000	4.010	9.38E-01	False
PAK1	11317.354	8049.247	1994.477	1.406	9.38E-01	False
COL4A3BP	74.818	50.748	14.441	1.474	9.39E-01	False
PGK2	65.359	44.344	12.839	1.474	9.39E-01	False
EPHA8	67.509	45.752	13.191	1.476	9.39E-01	False
TBK1	3.010	1.397	1.231	2.155	9.39E-01	False
MAPK11	436.870	306.877	77.956	1.424	9.40E-01	False
NADK	6.450	3.190	1.953	2.022	9.40E-01	False
BRDT	23.219	14.667	5.277	1.583	9.41E-01	False
PLK1	6.020	3.180	1.949	1.893	9.41E-01	False
IGF1R	2.150	0.838	0.939	2.565	9.43E-01	False
STK10	22.360	13.906	5.075	1.608	9.43E-01	False
TSSK1B	15.910	9.104	3.766	1.748	9.43E-01	False
PAK4	92.878	62.845	17.460	1.478	9.44E-01	False
RPS6KL1	96.318	65.564	18.137	1.469	9.44E-01	False
Non-Targeting_Control_074	1298.142	910.316	227.345	1.426	9.44E-01	False
PIP5K1A	218.865	151.193	39.393	1.448	9.45E-01	False
HCK	27.089	17.267	5.961	1.569	9.45E-01	False
DMPK	5.160	2.513	1.703	2.053	9.45E-01	False
PIK3CD	919.750	642.948	161.158	1.431	9.45E-01	False
GNE	295.834	204.909	52.703	1.444	9.45E-01	False
ULK2	38.699	24.823	7.911	1.559	9.46E-01	False
DGKZ	361.622	250.865	64.086	1.442	9.46E-01	False
CSNK1G2	6.020	3.109	1.924	1.936	9.46E-01	False
PFKL	35.259	22.732	7.375	1.551	9.46E-01	False
ERBB4	20.210	12.389	4.668	1.631	9.46E-01	False
XRCC6BP1	13.330	7.652	3.353	1.742	9.46E-01	False
STK40	5.160	2.489	1.694	2.073	9.47E-01	False
INSR	88.578	59.608	16.653	1.486	9.47E-01	False
Non-Targeting_Control_028	530.608	368.631	93.247	1.439	9.47E-01	False
CDKL2	270.464	186.356	48.107	1.451	9.48E-01	False
AK2	18.490	10.963	4.281	1.687	9.48E-01	False
RPS6KB2	34.829	21.922	7.167	1.589	9.48E-01	False
VRK1	27.949	17.043	5.903	1.640	9.49E-01	False
NTRK1	2028.266	1407.998	350.541	1.441	9.50E-01	False
DOLK	2.150	0.784	0.906	2.742	9.51E-01	False
EIF2AK3	952.429	656.820	164.592	1.450	9.52E-01	False
CSNK1G3	3.010	1.285	1.178	2.342	9.52E-01	False
PRKG1	6.450	3.018	1.891	2.137	9.52E-01	False
RIPK3	9.030	4.892	2.522	1.846	9.52E-01	False
CDK18	4.730	1.834	1.428	2.579	9.52E-01	False
CAMK4	15.480	8.785	3.676	1.762	9.53E-01	False
PRKAR2A	15.910	8.785	3.676	1.811	9.53E-01	False
TLK2	24.509	14.807	5.314	1.655	9.53E-01	False
TAF9	14.620	8.108	3.484	1.803	9.54E-01	False
MAP2K1	205.966	138.963	36.362	1.482	9.54E-01	False
MINK1	3.870	1.262	1.166	3.067	9.55E-01	False
MYLK	30.959	18.764	6.351	1.650	9.55E-01	False
MST1	9.890	4.819	2.498	2.052	9.56E-01	False
MUSK	26.229	16.032	5.637	1.636	9.56E-01	False
PKLR	2.580	0.747	0.884	3.455	9.56E-01	False
ATMIN	4.300	1.787	1.408	2.407	9.56E-01	False
MAP3K13	34.399	21.413	7.036	1.606	9.56E-01	False
CSNK2A2	33.539	20.674	6.846	1.622	9.57E-01	False
MAPK10	53.749	34.233	10.297	1.570	9.57E-01	False
PLXNA4	9545.362	6519.683	1615.858	1.464	9.58E-01	False
PKD2	21.070	12.599	4.725	1.672	9.58E-01	False
INSRR	2771.720	1889.314	469.684	1.467	9.58E-01	False
EIF2AK4	8.170	4.132	2.275	1.977	9.58E-01	False
TGFBR3	22.790	13.209	4.889	1.725	9.59E-01	False
EPHB1	63.639	40.848	11.962	1.558	9.59E-01	False
NTRK1	5.160	2.311	1.624	2.232	9.59E-01	False
ACVR2B	17.200	9.867	3.979	1.743	9.60E-01	False
PKN2	65.789	41.944	12.237	1.568	9.61E-01	False
PRKRA	34.399	21.091	6.953	1.631	9.61E-01	False
NME9	30.099	18.402	6.257	1.636	9.61E-01	False
EPHB3	44.289	27.676	8.638	1.600	9.62E-01	False
RIPK4	21.500	12.346	4.657	1.741	9.63E-01	False
KSR1	10.320	5.258	2.637	1.963	9.63E-01	False
ROR2	19.350	11.025	4.298	1.755	9.63E-01	False
NEK6	326.793	217.200	55.748	1.505	9.63E-01	False
DYRK1B	137.597	89.940	24.199	1.530	9.64E-01	False
PIP5K1A	52.889	32.771	9.928	1.614	9.65E-01	False

CSNK1G2	95.028	61.500	17.125	1.545	9.65E-01	False
Non-Targeting_Control_047	18.920	10.296	4.098	1.838	9.65E-01	False
NPR2	591.237	393.522	99.409	1.502	9.65E-01	False
ETNK1	279.494	184.456	47.636	1.515	9.65E-01	False
SPEG	42.999	25.997	8.210	1.654	9.66E-01	False
CDKL1	7.740	3.352	2.010	2.309	9.66E-01	False
NEK5	21.500	12.167	4.608	1.767	9.66E-01	False
MAP2K6	422.681	278.648	70.966	1.517	9.67E-01	False
PGM2L1	6.450	2.733	1.786	2.360	9.68E-01	False
RAB11FIP5	401.181	263.993	67.337	1.520	9.68E-01	False
TEC	27.089	15.969	5.621	1.696	9.68E-01	False
MAP3K7	32.679	19.215	6.468	1.701	9.68E-01	False
PRKCG	587.367	387.150	97.832	1.517	9.68E-01	False
MATK	2686.152	1778.112	442.157	1.511	9.69E-01	False
DAPK2	7.310	3.280	1.985	2.228	9.69E-01	False
NLK	191.346	123.709	32.580	1.547	9.70E-01	False
CDK5R1	142.327	91.304	24.538	1.559	9.70E-01	False
CDK16	8.170	3.836	2.176	2.130	9.71E-01	False
BRD3	4.300	1.585	1.319	2.713	9.71E-01	False
TAF9	7.740	3.243	1.972	2.387	9.71E-01	False
SH3BP5	696.155	454.461	114.496	1.532	9.72E-01	False
HK1	321.633	208.251	53.531	1.544	9.72E-01	False
DGKD	117.387	74.435	20.345	1.577	9.72E-01	False
PTK2B	1.720	0.252	0.506	6.816	9.72E-01	False
DGKZ	52.459	31.903	9.709	1.644	9.72E-01	False
IP6K3	101.908	63.891	17.720	1.595	9.72E-01	False
MAPKAPK2	18.490	9.935	3.998	1.861	9.72E-01	False
NEK8	426.121	276.694	70.482	1.540	9.72E-01	False
ALPK3	20.640	11.190	4.343	1.844	9.72E-01	False
CAMK2A	1.720	0.249	0.503	6.910	9.73E-01	False
HK1	41.279	24.674	7.873	1.673	9.73E-01	False
FYN	8.600	3.771	2.155	2.280	9.73E-01	False
FGFR4	509.109	330.136	83.715	1.542	9.73E-01	False
CSNK1D	12.040	6.154	2.911	1.957	9.73E-01	False
CDK11A	6.450	2.611	1.740	2.470	9.74E-01	False
RPS6KA6	10.750	4.909	2.527	2.190	9.74E-01	False
STRADB	13.760	6.722	3.081	2.047	9.74E-01	False
DAPK1	30.959	17.426	6.003	1.777	9.74E-01	False
RPS6KC1	18.920	9.801	3.961	1.930	9.75E-01	False
MYLK	578.767	372.903	94.304	1.552	9.75E-01	False
MEX3B	29.239	16.736	5.822	1.747	9.75E-01	False
ACTR2	13.330	6.689	3.071	1.993	9.75E-01	False
PRKD3	120.397	75.387	20.582	1.597	9.75E-01	False
HK2	1670.084	1079.355	269.189	1.547	9.76E-01	False
Non-Targeting_Control_063	7.310	3.109	1.924	2.351	9.76E-01	False
RPS6KA2	32.679	18.505	6.284	1.766	9.77E-01	False
EPHA6	7695.973	4964.429	1230.880	1.550	9.77E-01	False
PNCK	12.470	6.018	2.870	2.072	9.77E-01	False
MYLK4	3.010	1.003	1.032	3.002	9.77E-01	False
ABL1	74.818	45.278	13.073	1.652	9.77E-01	False
DOLK	19.780	10.274	4.092	1.925	9.77E-01	False
MAPK10	3.440	0.999	1.030	3.443	9.77E-01	False
PAK3	394.301	250.817	64.074	1.572	9.77E-01	False
Non-Targeting_Control_034	34.829	19.662	6.584	1.771	9.78E-01	False
ROCK2	33.969	19.003	6.413	1.788	9.78E-01	False
MAP2K2	32.249	18.330	6.238	1.759	9.78E-01	False
PRKCG	241.225	151.839	39.554	1.589	9.78E-01	False
NRBP2	2.150	0.559	0.760	3.848	9.79E-01	False
ACVR2A	224.025	140.777	36.812	1.591	9.79E-01	False
DCLK1	15.480	7.722	3.373	2.005	9.79E-01	False
PIK3CA	108.358	66.331	18.328	1.634	9.80E-01	False
IKBKE	51.599	29.913	9.205	1.725	9.81E-01	False
CCL2	353.882	220.773	56.633	1.603	9.81E-01	False
DYRK4	38.699	21.744	7.121	1.780	9.81E-01	False
EPHA1	312.603	194.724	50.180	1.605	9.81E-01	False
NMRK1	333.673	207.971	53.461	1.604	9.81E-01	False
OSR1	20.640	10.557	4.170	1.955	9.82E-01	False
Non-Targeting_Control_066	85.138	51.019	14.509	1.669	9.82E-01	False
GK2	15.050	7.487	3.305	2.010	9.83E-01	False
MAPKAPK3	1564.736	979.544	244.481	1.597	9.83E-01	False
HIPK2	6.020	2.365	1.646	2.545	9.83E-01	False
NTRK1	1725.552	1080.020	269.353	1.598	9.83E-01	False
PDK1	101.908	60.833	16.958	1.675	9.83E-01	False

MORN1	24.509	12.896	4.805	1.900	9.83E-01	False
TWF1	8.600	3.439	2.041	2.501	9.83E-01	False
TESK1	115.237	69.311	19.070	1.663	9.84E-01	False
Non-Targeting_Control_036	67.939	39.386	11.595	1.725	9.84E-01	False
TNK1	47.729	26.962	8.456	1.770	9.84E-01	False
PKM	287.234	176.525	45.671	1.627	9.84E-01	False
FASTKD3	199.086	121.588	32.054	1.637	9.84E-01	False
Non-Targeting_Control_100	770.543	477.281	120.146	1.614	9.84E-01	False
WEE2	12.040	5.636	2.753	2.136	9.84E-01	False
ALPK1	76.538	44.635	12.912	1.715	9.85E-01	False
RET	147.057	88.594	23.865	1.660	9.85E-01	False
NRK	95.888	56.340	15.838	1.702	9.85E-01	False
ADCK4	10.320	4.445	2.378	2.322	9.85E-01	False
MAP3K1	7.310	2.803	1.812	2.608	9.85E-01	False
CSK	44.719	24.806	7.906	1.803	9.85E-01	False
UHMK1	40.419	22.304	7.265	1.812	9.86E-01	False
Non-Targeting_Control_010	724.534	444.647	112.067	1.629	9.86E-01	False
CLK1	18.920	9.015	3.741	2.099	9.86E-01	False
CSK	51.169	28.942	8.959	1.768	9.86E-01	False
PIK3R4	22.790	11.358	4.389	2.007	9.86E-01	False
NAGK	508.679	310.181	78.774	1.640	9.86E-01	False
BMP2K	102.768	60.178	16.795	1.708	9.86E-01	False
IRAK1	13258.761	8156.333	2020.984	1.626	9.87E-01	False
PRKAB1	150.497	89.593	24.113	1.680	9.87E-01	False
MYLK	87.718	50.883	14.475	1.724	9.87E-01	False
DYRK1B	321.633	194.677	50.168	1.652	9.87E-01	False
NEK9	101.048	59.398	16.601	1.701	9.87E-01	False
PFKFB3	666.485	406.298	102.572	1.640	9.87E-01	False
ETNK2	26.229	13.680	5.015	1.917	9.87E-01	False
WEE1	8.170	3.270	1.981	2.499	9.87E-01	False
DCLK3	3.440	0.838	0.939	4.105	9.87E-01	False
CSNK1G3	3.870	0.838	0.939	4.618	9.87E-01	False
NUAK1	14.620	6.605	3.046	2.213	9.87E-01	False
PAK3	435.151	264.015	67.342	1.648	9.87E-01	False
MAP3K13	8.170	3.261	1.978	2.505	9.87E-01	False
PACSIN1	4.300	1.262	1.166	3.408	9.87E-01	False
C8orf44-SGK3	602.417	365.826	92.552	1.647	9.87E-01	False
FYN	20.210	10.033	4.025	2.014	9.88E-01	False
EPHA5	32.679	17.122	5.923	1.909	9.88E-01	False
ROR2	9.460	3.748	2.147	2.524	9.88E-01	False
FGFR2	78.258	44.693	12.926	1.751	9.89E-01	False
NEK3	4981.872	3013.955	748.072	1.653	9.89E-01	False
SGK1	107.498	61.768	17.191	1.740	9.90E-01	False
XYLB	45.579	24.564	7.844	1.856	9.90E-01	False
IRAK1	24.079	12.139	4.601	1.984	9.90E-01	False
TNK2	464.820	276.598	70.458	1.680	9.90E-01	False
ARSG	16.340	7.510	3.311	2.176	9.90E-01	False
TJP2	3.010	0.777	0.902	3.873	9.90E-01	False
MARK3	59.769	32.842	9.946	1.820	9.90E-01	False
TNK1	398.601	236.520	60.533	1.685	9.90E-01	False
EPHA8	621.336	370.258	93.649	1.678	9.90E-01	False
EPHB3	231.765	135.923	35.608	1.705	9.90E-01	False
STK19	86.428	48.849	13.966	1.769	9.90E-01	False
SH3BP5	61.919	33.916	10.218	1.826	9.90E-01	False
IRAK2	3.010	0.757	0.890	3.976	9.91E-01	False
ULK1	42.999	22.475	7.309	1.913	9.91E-01	False
VRK1	234.775	136.694	35.799	1.718	9.91E-01	False
CMPK1	8.600	3.055	1.904	2.815	9.91E-01	False
CAMK2D	1371.240	813.211	203.307	1.686	9.91E-01	False
ROS1	637.246	376.214	95.124	1.694	9.91E-01	False
ERBB2	263.584	153.597	39.989	1.716	9.91E-01	False
STK4	77.398	43.053	12.515	1.798	9.91E-01	False
ABCC1	80.838	44.786	12.949	1.805	9.91E-01	False
DYRK2	9.890	3.543	2.076	2.792	9.91E-01	False
MAP3K3	20.640	9.512	3.880	2.170	9.92E-01	False
TRPM6	73.958	40.340	11.834	1.833	9.92E-01	False
GSG2	127.707	72.125	19.770	1.771	9.92E-01	False
PRPS1L1	125.987	70.786	19.437	1.780	9.92E-01	False
NME5	6.450	1.995	1.496	3.233	9.92E-01	False
CAMKK2	1580.646	927.257	231.538	1.705	9.92E-01	False
ULK1	121.257	68.274	18.812	1.776	9.92E-01	False
NME3	576.617	335.786	85.114	1.717	9.92E-01	False
STK25	18.920	8.318	3.544	2.275	9.92E-01	False

CKMT2	123.837	69.402	19.093	1.784	9.93E-01	False
FLT4	1117.116	653.422	163.751	1.710	9.93E-01	False
STK39	960.599	560.989	140.869	1.712	9.93E-01	False
CDC42BPA	70.518	38.289	11.319	1.842	9.93E-01	False
PIP5KL1	90.728	49.861	14.219	1.820	9.93E-01	False
ABCC1	47.299	24.828	7.912	1.905	9.93E-01	False
PRPF4B	9.460	3.432	2.038	2.756	9.93E-01	False
NME7	19.350	8.801	3.680	2.199	9.93E-01	False
PRKD1	58.909	31.028	9.488	1.899	9.93E-01	False
MAP3K9	30.959	14.888	5.336	2.080	9.93E-01	False
NTRK1	63.639	33.766	10.180	1.885	9.94E-01	False
NEK9	98.898	53.965	15.245	1.833	9.94E-01	False
MERTK	236.495	133.728	35.064	1.768	9.94E-01	False
MTOR	1031.118	594.177	149.085	1.735	9.94E-01	False
TNK2	56.329	29.607	9.128	1.903	9.94E-01	False
PRKAR1B	9.890	3.338	2.005	2.963	9.94E-01	False
PRPS1	86.428	46.830	13.461	1.846	9.94E-01	False
PIP4K2B	138.027	76.795	20.932	1.797	9.94E-01	False
CAMK1	203.816	114.264	30.237	1.784	9.94E-01	False
AK8	2278.950	1312.616	326.930	1.736	9.94E-01	False
TGFBR3	32.249	15.804	5.577	2.041	9.94E-01	False
CRKL	5565.369	3186.225	790.715	1.747	9.95E-01	False
PRKCB	72.238	38.333	11.330	1.884	9.95E-01	False
MPP1	905.130	514.597	129.384	1.759	9.95E-01	False
CDK9	15.050	6.298	2.954	2.390	9.95E-01	False
ADRBK1	164.256	90.349	24.301	1.818	9.95E-01	False
MOS	8.600	2.733	1.786	3.147	9.95E-01	False
CKMT2	237.785	131.770	34.578	1.805	9.95E-01	False
MAPK3	22.360	10.013	4.019	2.233	9.95E-01	False
STK35	184.896	101.497	27.069	1.822	9.95E-01	False
PIK3R5	6.450	1.803	1.415	3.577	9.95E-01	False
MAGI1	947.269	534.565	134.327	1.772	9.95E-01	False
FASTK	39.989	19.359	6.506	2.066	9.95E-01	False
MAP3K1	126.847	68.338	18.828	1.856	9.95E-01	False
MYLK3	408.491	228.059	58.437	1.791	9.95E-01	False
KALRN	81.268	42.849	12.464	1.897	9.96E-01	False
CAMKK1	23.649	10.471	4.146	2.258	9.96E-01	False
DGKZ	30.529	14.268	5.171	2.140	9.96E-01	False
FN3K	252.834	138.885	36.343	1.820	9.96E-01	False
GRK5	6.880	1.763	1.398	3.902	9.96E-01	False
CDK17	32.679	15.322	5.450	2.133	9.96E-01	False
MASTL	62.349	31.973	9.727	1.950	9.96E-01	False
MPP6	153.937	82.949	22.462	1.856	9.96E-01	False
LIMK1	67.079	34.700	10.415	1.933	9.96E-01	False
STK38L	98.898	52.007	14.756	1.902	9.96E-01	False
MAP3K14	230.045	125.859	33.113	1.828	9.96E-01	False
MASTL	64.929	32.889	9.958	1.974	9.96E-01	False
CAMKK1	104.058	55.025	15.509	1.891	9.96E-01	False
RPS6KA2	71.378	36.628	10.901	1.949	9.96E-01	False
PLK4	124.267	66.072	18.264	1.881	9.96E-01	False
EPHA10	322.493	176.327	45.622	1.829	9.96E-01	False
BTK	8.170	2.598	1.735	3.145	9.96E-01	False
STK11	10.320	3.539	2.075	2.916	9.96E-01	False
CAMK2A	119.967	63.088	17.520	1.902	9.97E-01	False
SEPHS2	2.150	0.279	0.533	7.696	9.97E-01	False
STK36	2.150	0.279	0.533	7.696	9.97E-01	False
CDC42BPG	2.150	0.279	0.533	7.696	9.97E-01	False
PFKFB2	11.180	3.993	2.229	2.800	9.97E-01	False
SCYL2	96.748	50.190	14.302	1.928	9.97E-01	False
CAMK2A	3.010	0.559	0.760	5.387	9.97E-01	False
CLK4	3.440	0.559	0.760	6.157	9.97E-01	False
PRKAG1	3.870	0.559	0.760	6.926	9.97E-01	False
GCK	16.770	6.499	3.015	2.580	9.97E-01	False
STRADB	124.267	65.605	18.147	1.894	9.97E-01	False
PLAU	42.139	20.405	6.776	2.065	9.97E-01	False
GUCY2C	615.317	337.601	85.564	1.823	9.97E-01	False
SCYL2	24.079	10.655	4.197	2.260	9.97E-01	False
HSPB8	911.150	500.932	126.001	1.819	9.97E-01	False
PI4K2A	22.790	9.566	3.895	2.382	9.97E-01	False
PIK3R5	39.129	18.673	6.328	2.095	9.97E-01	False
PFKFB3	216.715	116.028	30.674	1.868	9.97E-01	False
NME1-NME2	29.239	13.270	4.905	2.203	9.97E-01	False
CIB4	803.223	439.668	110.834	1.827	9.97E-01	False

TKFC	46.869	22.428	7.297	2.090	9.97E-01	False
TK2	203.386	108.673	28.849	1.872	9.97E-01	False
STK3	23.219	10.037	4.026	2.313	9.97E-01	False
TK1	144.477	75.930	20.717	1.903	9.97E-01	False
PASK	18.060	7.390	3.277	2.444	9.97E-01	False
DGKH	75.678	38.013	11.249	1.991	9.97E-01	False
TRIO	181.456	95.990	25.701	1.890	9.97E-01	False
DAPK1	14.620	5.353	2.666	2.731	9.97E-01	False
DYRK4	300.563	161.135	41.857	1.865	9.97E-01	False
MAST3	89.868	45.575	13.147	1.972	9.97E-01	False
Non-Targeting_Control_087	83.848	42.242	12.312	1.985	9.97E-01	False
NUAK1	37.839	17.323	5.976	2.184	9.97E-01	False
NUAK2	2.150	0.252	0.506	8.520	9.97E-01	False
DGKE	207.685	109.574	29.073	1.895	9.97E-01	False
EIF2AK4	129.427	67.024	18.501	1.931	9.97E-01	False
CDK17	2.150	0.249	0.503	8.637	9.98E-01	False
MYLK3	46.869	22.010	7.190	2.129	9.98E-01	False
PRKD2	28.809	12.439	4.682	2.316	9.98E-01	False
ZAK	7.310	1.998	1.498	3.658	9.98E-01	False
BRD3	42.139	19.829	6.627	2.125	9.98E-01	False
TK1	3.010	0.505	0.721	5.964	9.98E-01	False
ZAP70	3.010	0.505	0.721	5.964	9.98E-01	False
DGKG	17.630	6.729	3.083	2.620	9.98E-01	False
ERN2	1237.943	667.049	167.125	1.856	9.98E-01	False
STK36	3.010	0.501	0.719	6.005	9.98E-01	False
XYLB	3.870	0.501	0.719	7.720	9.98E-01	False
PIK3R3	304.003	161.189	41.871	1.886	9.98E-01	False
STRADA	3.010	0.498	0.716	6.046	9.98E-01	False
CSK	7753.591	4187.827	1038.645	1.851	9.98E-01	False
MINK1	8310.859	4486.113	1112.481	1.853	9.98E-01	False
MST1R	9.890	2.836	1.825	3.487	9.98E-01	False
SRMS	18.490	7.178	3.215	2.576	9.98E-01	False
PDK3	68.799	33.510	10.115	2.053	9.98E-01	False
PLK3	33.539	14.878	5.333	2.254	9.98E-01	False
FLT1	311.743	163.926	42.549	1.902	9.98E-01	False
MAPKAPK5	15.050	5.639	2.755	2.669	9.98E-01	False
PSKH2	15374.315	8260.333	2046.728	1.861	9.98E-01	False
AK7	361.622	190.589	49.156	1.897	9.98E-01	False
CSNK2A2	8.170	0.000	0.000	9.170	9.98E-01	False
MAPK12	1284.382	685.007	171.570	1.875	9.98E-01	False
PSKH2	343.993	180.075	46.551	1.910	9.98E-01	False
CHKB	264.444	137.735	36.057	1.920	9.98E-01	False
ADCK3	59.769	28.330	8.804	2.110	9.98E-01	False
Non-Targeting_Control_005	36.549	16.239	5.692	2.251	9.98E-01	False
CIT	29.239	12.607	4.727	2.319	9.98E-01	False
TRIM27	5.160	1.117	1.093	4.618	9.98E-01	False
MAPK11	104.058	52.110	14.782	1.997	9.98E-01	False
TSSK3	34.399	15.167	5.409	2.268	9.98E-01	False
PASK	457.940	239.946	61.381	1.909	9.98E-01	False
PAN3	156.947	79.655	21.643	1.970	9.98E-01	False
NTPCR	238.645	122.884	32.375	1.942	9.98E-01	False
SLAMF6	4.300	0.757	0.890	5.680	9.98E-01	False
CMPK2	31.389	13.510	4.969	2.323	9.98E-01	False
PDK4	17305.403	9151.803	2267.396	1.891	9.98E-01	False
SBK1	30.529	12.948	4.819	2.358	9.98E-01	False
JAK2	44.289	20.099	6.697	2.204	9.99E-01	False
CDK19	8805.778	4637.207	1149.882	1.899	9.99E-01	False
ERBB3	1088.306	569.439	142.961	1.911	9.99E-01	False
ITPKA	68.369	32.512	9.863	2.103	9.99E-01	False
MAP4K1	210.265	106.598	28.334	1.973	9.99E-01	False
TAOK3	128.997	63.636	17.657	2.027	9.99E-01	False
PANK2	9.460	2.631	1.748	3.595	9.99E-01	False
VRK3	68.369	32.310	9.812	2.116	9.99E-01	False
MAP4K2	37.409	16.310	5.710	2.294	9.99E-01	False
ROR1	20.210	7.759	3.383	2.605	9.99E-01	False
MAP3K5	167.266	83.823	22.679	1.995	9.99E-01	False
NEK9	70.518	33.275	10.056	2.119	9.99E-01	False
PLK5	50.739	22.929	7.426	2.213	9.99E-01	False
NLK	650.146	335.811	85.120	1.936	9.99E-01	False
DGKB	7.310	1.780	1.405	4.107	9.99E-01	False
OBSCN	39.129	17.233	5.952	2.271	9.99E-01	False
GRK4	58.479	26.960	8.455	2.169	9.99E-01	False
RNASEL	13.330	4.331	2.341	3.078	9.99E-01	False

NPR2	11.180	3.412	2.031	3.277	9.99E-01	False
CHKB	279.494	140.044	36.630	1.996	9.99E-01	False
MAP2K5	253.694	126.551	33.284	2.005	9.99E-01	False
MAP3K10	64.929	29.551	9.114	2.197	9.99E-01	False
PACSIN1	676.805	343.578	87.043	1.970	9.99E-01	False
SRPK3	277.774	138.644	36.283	2.004	9.99E-01	False
CDC42BPB	41.279	17.865	6.117	2.311	9.99E-01	False
CAMK2D	100.618	47.523	13.635	2.117	9.99E-01	False
GLYCTK	27.519	10.805	4.238	2.547	9.99E-01	False
CLK4	17.630	6.012	2.868	2.933	9.99E-01	False
FN3K	18.490	6.474	3.007	2.856	9.99E-01	False
BRD4	34.829	14.210	5.156	2.451	9.99E-01	False
CSNK1A1L	782.153	394.077	99.547	1.985	9.99E-01	False
MST1R	138.887	66.546	18.382	2.087	9.99E-01	False
BRSK1	171.566	83.230	22.532	2.061	9.99E-01	False
SGK223	20406.496	10355.064	2565.244	1.971	9.99E-01	False
GK2	21.930	7.822	3.402	2.804	9.99E-01	False
ACVR2A	362.052	179.152	46.322	2.021	9.99E-01	False
ERBB3	133.297	63.211	17.551	2.109	9.99E-01	False
PAK4	116.527	54.690	15.426	2.131	9.99E-01	False
SGK3	94.598	43.652	12.665	2.167	9.99E-01	False
PSKH1	1217.304	604.755	151.703	2.013	9.99E-01	False
MAP2K2	11.610	3.160	1.942	3.674	9.99E-01	False
PFKM	20.640	7.150	3.207	2.887	9.99E-01	False
ITPKA	94.598	43.231	12.560	2.188	9.99E-01	False
NME1-NME2	23.219	8.514	3.599	2.727	9.99E-01	False
PIP5K1A	11.180	3.085	1.915	3.624	1.00E+00	False
DAPK3	69.658	30.577	9.374	2.278	1.00E+00	False
MAPKAPK2	574.897	279.416	71.156	2.057	1.00E+00	False
OBSCN	39.989	15.937	5.612	2.509	1.00E+00	False
Non-Targeting_Control_061	287.234	137.601	36.024	2.087	1.00E+00	False
STK16	80.838	35.688	10.664	2.265	1.00E+00	False
WNK3	641.976	310.024	78.735	2.071	1.00E+00	False
MAPK8	67.509	29.225	9.031	2.310	1.00E+00	False
SCYL2	15.910	4.640	2.441	3.429	1.00E+00	False
NMRK2	73.528	31.899	9.708	2.305	1.00E+00	False
GRK6	71.378	30.905	9.457	2.310	1.00E+00	False
BCR	73.958	31.778	9.678	2.327	1.00E+00	False
DMPK	8.600	1.790	1.409	4.804	1.00E+00	False
PRKCE	43.859	17.423	6.002	2.517	1.00E+00	False
JAK3	304.003	143.095	37.386	2.124	1.00E+00	False
TKFC	58.909	24.506	7.830	2.404	1.00E+00	False
FGFR2	251.975	117.367	31.007	2.147	1.00E+00	False
PKN1	77.828	33.521	10.118	2.322	1.00E+00	False
LRRK1	6.020	1.087	1.077	5.538	1.00E+00	False
PTK6	5.590	0.781	0.904	7.161	1.00E+00	False
STK4	3.010	0.279	0.533	10.775	1.00E+00	False
WEE2	54.179	22.324	7.270	2.427	1.00E+00	False
MAP3K11	78.688	33.653	10.151	2.338	1.00E+00	False
DTYMK	6.020	1.057	1.061	5.698	1.00E+00	False
ITPKB	398.601	186.354	48.107	2.139	1.00E+00	False
PKN3	195.646	89.180	24.010	2.194	1.00E+00	False
NEK2	57.189	23.595	7.597	2.424	1.00E+00	False
MAP2K7	154.367	69.388	19.089	2.225	1.00E+00	False
CDK18	15.480	4.411	2.367	3.509	1.00E+00	False
NUAK1	354.742	163.905	42.544	2.164	1.00E+00	False
CERK	6.450	1.026	1.044	6.286	1.00E+00	False
PRKCB	140.607	62.425	17.355	2.252	1.00E+00	False
BMPR2	905.130	423.531	106.839	2.137	1.00E+00	False
PRKAG2	166.836	74.528	20.368	2.239	1.00E+00	False
PFKP	76.108	32.178	9.779	2.365	1.00E+00	False
MAP3K5	321.633	146.882	38.325	2.190	1.00E+00	False
IKBKE	137.597	60.459	16.865	2.276	1.00E+00	False
DGKB	3.440	0.249	0.503	13.819	1.00E+00	False
MAP4K2	76.968	31.805	9.684	2.420	1.00E+00	False
TNNI3K	640.686	294.825	74.972	2.173	1.00E+00	False
IP6K1	46.869	17.946	6.138	2.612	1.00E+00	False
IP6K2	134.157	58.584	16.397	2.290	1.00E+00	False
TPD52L3	172.426	75.962	20.725	2.270	1.00E+00	False
MAP3K8	4875.234	2259.051	561.207	2.158	1.00E+00	False
WNK3	116.097	49.813	14.207	2.331	1.00E+00	False
CLK2	149.207	64.852	17.960	2.301	1.00E+00	False
KHK	127.277	54.686	15.425	2.327	1.00E+00	False

ACVRL1	383.122	172.370	44.642	2.223	1.00E+00	False
MAPK8	98.038	41.171	12.043	2.381	1.00E+00	False
TAOK2	86.858	35.527	10.624	2.445	1.00E+00	False
PANK3	1893.249	861.739	215.320	2.197	1.00E+00	False
PRKRA	27.089	9.071	3.757	2.986	1.00E+00	False
NMRK2	8.170	1.507	1.283	5.421	1.00E+00	False
CSK	67.509	26.727	8.396	2.526	1.00E+00	False
PIKFYVE	226.175	98.699	26.374	2.292	1.00E+00	False
STK16	246.815	107.504	28.559	2.296	1.00E+00	False
DAPK1	282.934	123.571	32.545	2.290	1.00E+00	False
DCLK3	291.534	127.160	33.435	2.293	1.00E+00	False
INSRR	885.781	394.454	99.640	2.246	1.00E+00	False
PAK2	167.266	70.791	19.438	2.363	1.00E+00	False
PRKAR2A	366.352	159.722	41.507	2.294	1.00E+00	False
CALM1	6.450	0.838	0.939	7.696	1.00E+00	False
MAP4K3	404.621	176.102	45.566	2.298	1.00E+00	False
Non-Targeting_Control_051	665.196	292.477	74.390	2.274	1.00E+00	False
IKBKB	17.200	4.613	2.432	3.729	1.00E+00	False
MAPK11	111.368	45.277	13.072	2.460	1.00E+00	False
CDK18	869.871	381.318	96.388	2.281	1.00E+00	False
RPS6KL1	2133.614	941.852	235.151	2.265	1.00E+00	False
PRKCI	30.529	9.880	3.983	3.090	1.00E+00	False
RIPK4	503.089	218.276	56.014	2.305	1.00E+00	False
MAPK4	10.750	2.012	1.503	5.343	1.00E+00	False
CSF1R	214.565	90.462	24.329	2.372	1.00E+00	False
BUB1	3988.593	1757.645	437.091	2.269	1.00E+00	False
IRAK3	97.178	38.850	11.460	2.501	1.00E+00	False
CRKL	552.108	238.749	61.085	2.313	1.00E+00	False
TNIK	30.959	9.762	3.950	3.171	1.00E+00	False
ANKK1	511.689	220.270	56.508	2.323	1.00E+00	False
CDK16	424.401	182.018	47.032	2.332	1.00E+00	False
PKDCC	17.200	4.442	2.377	3.872	1.00E+00	False
ALPK1	4609.070	2002.799	497.776	2.301	1.00E+00	False
MAPK9	948.129	405.907	102.475	2.336	1.00E+00	False
FLT4	4347.635	1876.758	466.576	2.317	1.00E+00	False
GRK7	1019.938	435.997	109.925	2.339	1.00E+00	False
NTRK2	156.087	63.169	17.540	2.471	1.00E+00	False
PFKM	658.316	279.709	71.229	2.354	1.00E+00	False
EPHA3	73.528	27.497	8.592	2.674	1.00E+00	False
PDGFRB	930.500	396.470	100.139	2.347	1.00E+00	False
NEK3	667.345	282.667	71.961	2.361	1.00E+00	False
ARSG	321.633	133.296	34.957	2.413	1.00E+00	False
PRPS1	2239.391	954.403	238.258	2.346	1.00E+00	False
PDGFRL	24.939	6.954	3.149	3.586	1.00E+00	False
CKMT1B	1004.888	425.349	107.289	2.363	1.00E+00	False
MAP3K8	179.306	72.359	19.828	2.478	1.00E+00	False
ARSG	106.208	41.096	12.024	2.584	1.00E+00	False
DGKQ	891.801	374.814	94.777	2.379	1.00E+00	False
PAPSS2	401.181	165.559	42.954	2.423	1.00E+00	False
ARAF	32.679	9.983	4.011	3.274	1.00E+00	False
SBK1	35.259	11.183	4.341	3.153	1.00E+00	False
CDC42BPB	117.387	45.301	13.078	2.591	1.00E+00	False
NEK3	59.769	21.018	6.934	2.844	1.00E+00	False
CDKL1	1504.107	631.747	158.386	2.381	1.00E+00	False
PFKM	1557.426	653.961	163.885	2.382	1.00E+00	False
EPHB6	613.167	254.194	64.910	2.412	1.00E+00	False
XRCC6BP1	26.659	7.535	3.319	3.538	1.00E+00	False
ACVRL1	165.116	65.169	18.039	2.534	1.00E+00	False
TK2	2181.343	911.758	227.702	2.392	1.00E+00	False
IPMK	78.688	28.482	8.842	2.763	1.00E+00	False
STK16	579.627	236.802	60.603	2.448	1.00E+00	False
ERN1	351.732	140.508	36.745	2.503	1.00E+00	False
LATS1	112.228	41.896	12.225	2.679	1.00E+00	False
SGK223	1240.093	505.537	127.141	2.453	1.00E+00	False
DGKE	36.549	11.086	4.315	3.297	1.00E+00	False
BTK	73.528	25.890	8.183	2.840	1.00E+00	False
N4BP2	451.920	180.831	46.738	2.499	1.00E+00	False
STK24	131.577	49.397	14.103	2.664	1.00E+00	False
KALRN	72.238	25.313	8.036	2.854	1.00E+00	False
IP6K2	17.630	3.863	2.186	4.564	1.00E+00	False
EPHB3	76.538	26.773	8.408	2.859	1.00E+00	False
BRDT	89.438	31.885	9.705	2.805	1.00E+00	False
PBK	1475.728	593.585	148.938	2.486	1.00E+00	False

MARK1	2360.219	949.265	236.986	2.486	1.00E+00	False
RPS6KA1	11.610	1.841	1.431	6.308	1.00E+00	False
MEX3B	115.237	41.800	12.201	2.757	1.00E+00	False
GTF2H1	8.600	1.003	1.032	8.578	1.00E+00	False
MLKL	518.569	203.065	52.246	2.554	1.00E+00	False
CAMK2B	221.445	83.596	22.623	2.649	1.00E+00	False
PINK1	7.310	0.747	0.884	9.789	1.00E+00	False
FGFR3	240.365	91.078	24.482	2.639	1.00E+00	False
PRKD2	598.977	232.422	59.518	2.577	1.00E+00	False
CAMK1G	5979.020	2365.324	587.513	2.528	1.00E+00	False
SRPK3	297.124	113.112	29.951	2.627	1.00E+00	False
SRMS	5754.995	2272.435	564.520	2.533	1.00E+00	False
BRAF	90.298	31.287	9.553	2.886	1.00E+00	False
CDK5	269.604	101.227	27.001	2.663	1.00E+00	False
SPHK2	218.005	81.092	22.000	2.688	1.00E+00	False
PINK1	199.516	73.506	20.114	2.714	1.00E+00	False
TRIB2	173.716	62.694	17.422	2.771	1.00E+00	False
CD2	83.418	27.899	8.694	2.990	1.00E+00	False
RBKS	19.350	4.122	2.272	4.695	1.00E+00	False
PLK5	5684.906	2192.537	544.742	2.593	1.00E+00	False
WNK1	76.968	24.917	7.935	3.089	1.00E+00	False
PIP5KL1	1063.797	404.084	102.024	2.633	1.00E+00	False
DCLK2	113.518	38.986	11.494	2.912	1.00E+00	False
SPHK2	131.577	45.759	13.193	2.875	1.00E+00	False
MPP4	104.488	35.308	10.569	2.959	1.00E+00	False
MAP4K1	75.248	24.305	7.778	3.096	1.00E+00	False
MAST1	265.304	96.302	25.779	2.755	1.00E+00	False
MAPKAPK5	1427.999	536.608	134.833	2.661	1.00E+00	False
AXL	1420.259	533.857	134.152	2.660	1.00E+00	False
CSNK2A2	98.038	32.699	9.910	2.998	1.00E+00	False
ITPKC	21.500	4.552	2.413	4.723	1.00E+00	False
PDK3	144.477	49.732	14.187	2.905	1.00E+00	False
TSSK1B	160.817	55.704	15.679	2.887	1.00E+00	False
Non-Targeting_Control_065	45.579	12.710	4.755	3.586	1.00E+00	False
PRKCD	754.204	275.311	70.140	2.739	1.00E+00	False
ADRBK1	70.088	21.541	7.069	3.254	1.00E+00	False
NADK2	25.799	5.649	2.758	4.567	1.00E+00	False
ADCK2	11156.537	4122.283	1022.421	2.706	1.00E+00	False
GALK1	73.958	22.580	7.336	3.275	1.00E+00	False
NEK10	1550.116	567.353	142.444	2.732	1.00E+00	False
NPR1	23.649	4.879	2.517	4.848	1.00E+00	False
PAK2	7.310	0.532	0.741	13.748	1.00E+00	False
PAN3	418.381	147.781	38.548	2.831	1.00E+00	False
PRKAG3	2656.052	955.816	238.608	2.779	1.00E+00	False
FGFR1	541.788	190.587	49.155	2.843	1.00E+00	False
Non-Targeting_Control_067	1440.899	509.106	128.025	2.830	1.00E+00	False
PCK2	429.131	147.269	38.421	2.914	1.00E+00	False
GSAP	173.286	56.225	15.809	3.082	1.00E+00	False
CAMK1D	321.203	107.626	28.590	2.984	1.00E+00	False
RPS6KB1	224.455	73.619	20.142	3.049	1.00E+00	False
RIPK2	140.177	44.264	12.819	3.167	1.00E+00	False
MAP3K4	49.019	12.849	4.792	3.815	1.00E+00	False
SGK2	7182.134	2505.092	622.111	2.867	1.00E+00	False
MAP2K1	88.148	26.078	8.231	3.380	1.00E+00	False
FASTK	3328.128	1150.612	286.828	2.892	1.00E+00	False
MAP4K3	100.618	30.006	9.229	3.353	1.00E+00	False
EIF2AK1	98.898	29.215	9.028	3.385	1.00E+00	False
CDK17	6.450	0.279	0.533	23.088	1.00E+00	False
PRKAG2	9780.137	3364.867	834.935	2.907	1.00E+00	False
TK2	397.311	131.724	34.567	3.016	1.00E+00	False
CDKL5	362.482	119.555	31.549	3.032	1.00E+00	False
BRSK1	505.669	168.292	43.631	3.005	1.00E+00	False
UCKL1	168.986	52.630	14.911	3.211	1.00E+00	False
Non-Targeting_Control_069	20.640	3.419	2.033	6.037	1.00E+00	False
XYLB	370.652	120.612	31.811	3.073	1.00E+00	False
MAP4K4	85.998	24.052	7.713	3.576	1.00E+00	False
C8orf44-SGK3	89.868	25.374	8.051	3.542	1.00E+00	False
PDGFRL	9397.876	3169.491	786.572	2.965	1.00E+00	False
DAPK3	556.408	182.210	47.080	3.054	1.00E+00	False
CSNK2A1	350.442	112.278	29.744	3.121	1.00E+00	False
CKS2	332.813	105.851	28.149	3.144	1.00E+00	False
KDR	269.604	84.162	22.763	3.203	1.00E+00	False
Non-Targeting_Control_060	47706.172	15802.632	3913.699	3.019	1.00E+00	False

NADK2	403.331	128.017	33.648	3.151	1.00E+00	False
STK38L	23.649	3.961	2.218	5.971	1.00E+00	False
PKM	38.269	8.341	3.550	4.588	1.00E+00	False
IGFN1	664.766	211.504	54.337	3.143	1.00E+00	False
FLT1	18.490	2.506	1.700	7.377	1.00E+00	False
DGKA	37.409	7.617	3.342	4.911	1.00E+00	False
ILK	251.545	74.284	20.307	3.386	1.00E+00	False
ITPKA	94.168	24.787	7.901	3.799	1.00E+00	False
NME9	361.192	108.527	28.813	3.328	1.00E+00	False
PAK3	662.186	202.443	52.092	3.271	1.00E+00	False
NEK6	392.151	117.741	31.099	3.331	1.00E+00	False
ULK3	92.018	23.720	7.629	3.879	1.00E+00	False
CIB1	704.755	210.975	54.206	3.340	1.00E+00	False
Non-Targeting_Control_030	1556.566	470.767	118.533	3.306	1.00E+00	False
NADK	20.640	2.591	1.733	7.967	1.00E+00	False
MAP2K7	1342.861	401.294	101.333	3.346	1.00E+00	False
TJP2	434.291	126.083	33.168	3.444	1.00E+00	False
IDNK	142.327	37.846	11.208	3.761	1.00E+00	False
AAK1	257.994	71.999	19.739	3.583	1.00E+00	False
MAP2K3	122.977	31.472	9.600	3.908	1.00E+00	False
NEK6	689.705	200.466	51.602	3.441	1.00E+00	False
ADPGK	396.021	112.858	29.888	3.509	1.00E+00	False
MYLK4	10.750	0.559	0.760	19.240	1.00E+00	False
PIP4K2A	142.327	37.089	11.017	3.837	1.00E+00	False
PI4K2A	402.041	114.111	30.199	3.523	1.00E+00	False
NEK5	138.027	35.665	10.658	3.870	1.00E+00	False
MAPK7	24.509	3.284	1.986	7.464	1.00E+00	False
Non-Targeting_Control_091	3260.619	930.806	232.417	3.503	1.00E+00	False
PRKCG	838.482	233.782	59.855	3.587	1.00E+00	False
MAPK4	37.409	6.279	2.949	5.958	1.00E+00	False
Non-Targeting_Control_024	813.542	226.518	58.056	3.592	1.00E+00	False
RAF1	52.889	10.201	4.071	5.185	1.00E+00	False
RIPK1	8.600	0.249	0.503	34.548	1.00E+00	False
PRKCI	214.995	55.223	15.559	3.893	1.00E+00	False
MAPK7	46.869	8.248	3.524	5.683	1.00E+00	False
CAMK2B	832.462	221.905	56.913	3.751	1.00E+00	False
ALDH18A1	113.948	25.462	8.074	4.475	1.00E+00	False
ULK2	294.544	73.746	20.174	3.994	1.00E+00	False
INSRR	3627.401	965.333	240.964	3.758	1.00E+00	False
PKD2	1561.726	405.873	102.467	3.848	1.00E+00	False
MAPK6	871.161	223.308	57.261	3.901	1.00E+00	False
BCR	1809.401	468.889	118.068	3.859	1.00E+00	False
MAP3K3	1745.332	445.830	112.360	3.915	1.00E+00	False
CDKL5	773.983	191.192	49.305	4.048	1.00E+00	False
FGGY	1464.548	366.110	92.622	4.000	1.00E+00	False
PHKB	693.575	170.211	44.107	4.075	1.00E+00	False
PRPS2	1094.326	271.786	69.267	4.026	1.00E+00	False
ARSG	400.321	94.916	25.435	4.218	1.00E+00	False
ZAP70	1790.481	444.656	112.069	4.027	1.00E+00	False
SGK3	2802.249	695.323	174.124	4.030	1.00E+00	False
HKDC1	1292.982	312.127	79.256	4.142	1.00E+00	False
MYLK	129.857	26.242	8.273	4.948	1.00E+00	False
PANK2	74.818	12.783	4.774	5.853	1.00E+00	False
CMPK2	35.259	4.115	2.270	8.569	1.00E+00	False
ABL1	131.577	25.832	8.168	5.094	1.00E+00	False
Non-Targeting_Control_006	654.446	149.982	39.093	4.363	1.00E+00	False
SLAMF6	37.839	4.404	2.365	8.591	1.00E+00	False
SGK1	340.123	73.859	20.202	4.605	1.00E+00	False
STK3	1340.281	306.432	77.846	4.374	1.00E+00	False
PIP5K1B	34.399	3.658	2.116	9.405	1.00E+00	False
BMPR1A	1184.624	268.413	68.431	4.413	1.00E+00	False
PIKFYVE	170.276	33.694	10.162	5.054	1.00E+00	False
ERN2	175.006	34.587	10.387	5.060	1.00E+00	False
MAPK8	240.795	49.220	14.059	4.892	1.00E+00	False
PDIK1L	3879.376	844.135	210.962	4.596	1.00E+00	False
NLK	731.844	148.213	38.655	4.938	1.00E+00	False
DDR2	314.753	63.422	17.604	4.963	1.00E+00	False
MAP3K13	309.593	60.128	16.782	5.149	1.00E+00	False
TPD52L3	397.741	76.211	20.787	5.219	1.00E+00	False
HSPB8	262.724	49.982	14.250	5.256	1.00E+00	False
PRKD2	341.413	64.275	17.816	5.312	1.00E+00	False
FGFR4	296.264	55.364	15.594	5.351	1.00E+00	False
OBSCN	10666.348	1962.198	487.725	5.436	1.00E+00	False

PRKAB2	256.704	45.803	13.204	5.605	1.00E+00	False
LMTK3	187.046	33.084	10.008	5.654	1.00E+00	False
CSNK2A1	112.228	17.455	6.010	6.430	1.00E+00	False
Non-Targeting_Control_062	194.356	29.669	9.143	6.551	1.00E+00	False
AMHR2	190.916	26.147	8.248	7.302	1.00E+00	False
TRIM28	227.465	30.300	9.304	7.507	1.00E+00	False
CAMKK2	240.795	31.246	9.543	7.706	1.00E+00	False
LATS2	138.457	17.689	6.071	7.827	1.00E+00	False
PLAU	214.135	24.065	7.717	8.898	1.00E+00	False
TYRO3	265.304	26.228	8.269	10.115	1.00E+00	False
MYO3B	241.225	23.207	7.497	10.394	1.00E+00	False
DDR1	125.987	11.558	4.443	10.900	1.00E+00	False
MAP3K19	46.009	3.129	1.931	14.703	1.00E+00	False
Non-Targeting_Control_095	148.777	9.321	3.827	15.961	1.00E+00	False
PAK4	15.480	0.498	0.716	31.093	1.00E+00	False
