# Supplementary material for: MEK1 as a Synthetic Lethal Target with Cabozantinib in Renal Cell Carcinoma: Insights from CRISPR/Cas9 Screening
Source: Genes (Basel). 2026 Jul 12;17(7):789. doi: 10.3390/genes17070789 (PMC13409911; doi:10.3390/genes17070789)
Supplement: Supplementary file 1 [file genes-17-00789-s001.zip › genes-4412660 Supplementary Table 2.pdf]

Supplementary Table 2. Significantly enriched pathways based on the putative synthetic lethal target genes for cabozantinib

Description	Genes	p-value
Central carbon metabolism in cancer	*PIK3CB, PIK3R3, PFKL, MAPK1, AKT3, FGFR1, MAP2K1*	3.47E-08
VEGF signalling pathway	*PIK3CB, PIK3R3, MAPK1, MAPKAPK3, AKT3, MAP2K1*	3.32E-07
Fc gamma R-mediated phagocytosis	*PIK3CB, PIK3R3, LIMK1, SYK, MAPK1, AKT3, MAP2K1*	3.06E-07
Prostate cancer	*PIK3CB, PIK3R3, CDK2, MAPK1, AKT3, FGFR1, MAP2K1*	3.28E-07
Yersinia infection	*PIK3CB, PIK3R3, RPS6KA2, LIMK1, IRAK4, MAPK1, AKT3, MAP2K1*	2.06E-07
Insulin signalling pathway	*PRKAR2B, PIK3CB, PIK3R3, MKNK1, PHKG1, MAPK1, AKT3, MAP2K1*	2.18E-07
HIF-1 signalling pathway	*PIK3CB, PIK3R3, PFKL, MKNK1, MAPK1, AKT3, MAP2K1*	6.81E-07
Progesterone-mediated oocyte maturation	*PIK3CB, PIK3R3, RPS6KA2, CDK2, MAPK1, AKT3, MAP2K1*	6.81E-07
Acute myeloid leukemia	*PIK3CB, PIK3R3, PIM2, MAPK1, AKT3, MAP2K1*	7.06E-07
