# Supplementary material for: MEK1 as a Synthetic Lethal Target with Cabozantinib in Renal Cell Carcinoma: Insights from CRISPR/Cas9 Screening
Source: Genes (Basel). 2026 Jul 12;17(7):789. doi: 10.3390/genes17070789 (PMC13409911; doi:10.3390/genes17070789)
Supplement: Supplementary file 1 [file genes-17-00789-s001.zip › genes-4412660 Supplementary Table 3.pdf]

Supplementary Table 3. Genes upregulated by cabozantinib treatment and downregulated by combination (cabozantinib + cobimetinib) treatment

	Control	Cabozantinib 6 hours			Cabozantinib 24 hours			Combination 6 hours			Combination 24 hours		
Gene Symbol	Counts	Counts	FDR	Log2 FC (Cabo/control)	Counts	FDR	Log2 FC (Cabo/control)	Counts	FDR	Log2 FC (Combo/Cabo)	Counts	FDR	Log2 FC (Combo/Cabo)
CTSO	41	90	0.008	1.116	285	0.000	2.659	68	0.984	-0.549	0	0.000	-11.104
FLRT1	27	36	0.605	0.397	64	0.004	1.208	27	1.000	-0.558	23	0.002	-1.640
TBCE	765	1055	0.136	0.447	837	0.731	0.097	737	0.081	-0.663	258	0.000	-1.866
SERF1A	20	60	0.002	1.563	69	0.000	1.747	40	0.756	-0.728	14	0.002	-1.937
TRIM27	20	155	0.000	2.930	88	0.000	2.171	97	0.110	-0.821	31	0.000	-1.670
GOLGA8N	20	56	0.005	1.463	99	0.000	2.267	38	0.844	-0.703	43	0.002	-1.369
CD99	1718	2521	0.053	0.537	3276	0.000	0.898	625	0.000	-2.157	262	0.000	-3.813
GTF2H4	22	143	0.000	2.677	65	0.000	1.525	101	0.476	-0.646	35	0.104	-1.059
EML5	36	51	0.419	0.485	86	0.001	1.220	30	0.480	-0.908	37	0.003	-1.383
PPP4R4	56	98	0.066	0.790	76	0.328	0.407	73	0.860	-0.569	78	0.109	-0.837
SPNS2	648	811	0.359	0.307	696	0.806	0.070	613	0.317	-0.549	450	0.015	-0.798
ABCC2	54	122	0.002	1.158	125	0.000	1.176	95	0.976	-0.505	86	0.257	-0.708
ZNF688	48	70	0.301	0.527	73	0.167	0.571	51	0.985	-0.601	47	0.298	-0.803
AQP11	18	39	0.079	1.094	53	0.001	1.518	30	1.000	-0.522	37	0.605	-0.686
RHOU	18	45	0.024	1.300	47	0.003	1.427	33	1.000	-0.591	33	0.636	-0.677
CTH	156	1576	0.000	3.319	415	0.000	1.378	886	0.000	-0.976	217	0.000	-1.104
FBXW10	25	52	0.054	1.037	61	0.004	1.250	38	1.000	-0.596	43	0.553	-0.672
GOLGA8H	28	70	0.005	1.302	47	0.000	3.882	45	0.574	-0.781	29	0.380	-0.863
RNF128	153	160	0.951	0.048	199	0.257	0.346	124	0.844	-0.512	153	0.395	-0.548
ULBP1	65	421	0.000	2.677	96	0.149	0.529	257	0.014	-0.857	58	0.119	-0.895
CUTA	312	459	0.086	0.540	777	0.000	1.283	2125	0.364	-0.507	2554	0.235	-0.513
RAB40A	45	95	0.010	1.060	125	0.000	1.438	74	1.000	-0.505	99	0.606	-0.505
ASNS	1198	5354	0.000	2.144	2354	0.000	0.941	3904	0.123	-0.601	1750	0.108	-0.597
CCDC169	21	42	0.104	0.980	73	0.000	1.758	27	0.958	-0.780	48	0.341	-0.772
CLEC20A	12	43	0.002	1.814	42	0.001	1.764	31	1.000	-0.615	31	0.758	-0.605
EEF1B2	2363	4473	0.000	0.904	2793	0.402	0.208	155	0.000	-4.995	137	0.000	-4.517
RAB3A	54	74	0.393	0.437	56	1.000	0.019	50	0.607	-0.709	41	0.649	-0.617
ARHGAP11B	96	113	0.707	0.219	45	0.000	3.820	68	0.131	-0.877	30	0.584	-0.751
CBS	390	1533	0.000	1.958	1084	0.000	1.441	1119	0.152	-0.599	858	0.283	-0.506
CCNB1IP1	254	425	0.014	0.726	642	0.000	1.304	276	0.043	-0.768	471	0.134	-0.616
DNM3	48	90	0.037	0.889	94	0.011	0.935	59	0.454	-0.753	70	0.545	-0.593
ATF6B	803	974	0.439	0.262	1485	0.000	1.042	564	0.101	-0.657	1117	0.281	-0.501
DDIT3	328	1753	0.000	2.401	1179	0.000	1.812	1180	0.032	-0.716	915	0.222	-0.535
SLC6A9	158	873	0.000	2.449	250	0.026	0.629	579	0.034	-0.737	193	0.371	-0.542
MDP1	3	71	0.000	4.492	37	0.000	3.538	27	0.003	-1.535	90	0.012	-1.016
EEFSEC	436	646	0.067	0.551	467	0.825	0.066	410	0.018	-0.801	364	0.303	-0.528
PLEK	31	49	0.275	0.642	171	0.000	2.426	25	0.256	-1.112	117	0.167	-0.716
CAPS	42	64	0.259	0.590	197	0.000	2.193	30	0.043	-1.235	128	0.077	-0.791
RDH5	45	102	0.004	1.162	98	0.003	1.088	59	0.110	-0.933	74	0.553	-0.574
TLE6	34	42	0.704	0.288	56	0.119	0.685	22	0.383	-1.074	40	0.649	-0.653
DHRS4	235	353	0.075	0.570	304	0.000	1.085	210	0.011	-0.894	241	0.412	-0.504
RDM1	34	59	0.136	0.777	56	0.079	0.772	18	0.030	-1.523	35	0.367	-0.845
MAP2	21	92	0.000	2.108	209	0.000	3.274	51	0.089	-0.994	161	0.389	-0.545
HLA-C	2113	2854	0.154	0.417	333	0.009	0.702	37	0.002	-1.456	218	0.041	-0.780
PCK2	593	2955	0.000	2.301	1628	0.000	1.424	1466	0.000	-0.993	1162	0.254	-0.513

MRNIP	40	61	0.269	0.591	103	0.000	1.329	6	0.000	-3.463	39	0.000	-1.567
INHBE	2	146	0.000	6.088	136	0.000	5.969	42	0.000	-1.939	85	0.089	-0.846
CDKN1C	6	43	0.000	2.800	88	0.000	3.814	20	0.174	-1.244	125	0.496	-0.534
CCHCR1	368	641	0.005	0.784	201	0.007	0.784	267	0.000	-1.408	158	0.466	-0.516
TIMM17B	408	434	0.880	0.073	663	0.479	0.193	118	0.000	-2.023	446	0.032	-0.741
TBC1D3L	9	45	0.000	2.290	56	0.000	2.588	15	0.011	-1.721	41	0.649	-0.617
PPP2R3B	33	116	0.000	1.793	73	0.005	1.109	46	0.000	-2.457	47	0.298	-0.803
ZBTB12	2	146	0.000	6.088	49	0.000	4.499	26	0.000	-2.628	37	0.476	-0.765
PFDN5	1024	1079	0.899	0.059	2325	0.000	1.060	126	0.000	-3.242	1484	0.005	-0.817
HMGN4	1146	1695	0.050	0.548	2878	0.006	0.631	32	0.000	-5.866	1818	0.004	-0.832
RNF187	1001	1584	0.017	0.646	2479	0.000	1.275	0	0.000	-13.704	767	0.000	-1.861
RPL17-C18orf32	200	286	0.148	0.499	233	0.549	0.187	0	0.000	-11.235	104	0.000	-1.332
