# Supplementary figures and images for: MEK1 as a Synthetic Lethal Target with Cabozantinib in Renal Cell Carcinoma: Insights from CRISPR/Cas9 Screening
Source: Genes (Basel). 2026 Jul 12;17(7):789. doi: 10.3390/genes17070789 (PMC13409911; doi:10.3390/genes17070789)

# Supplementary Figure S1

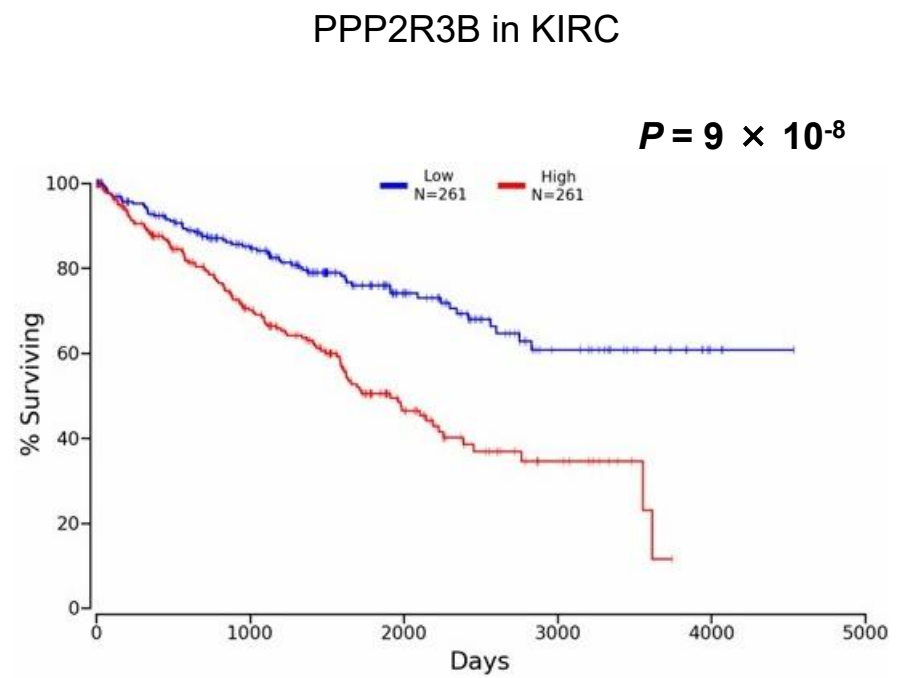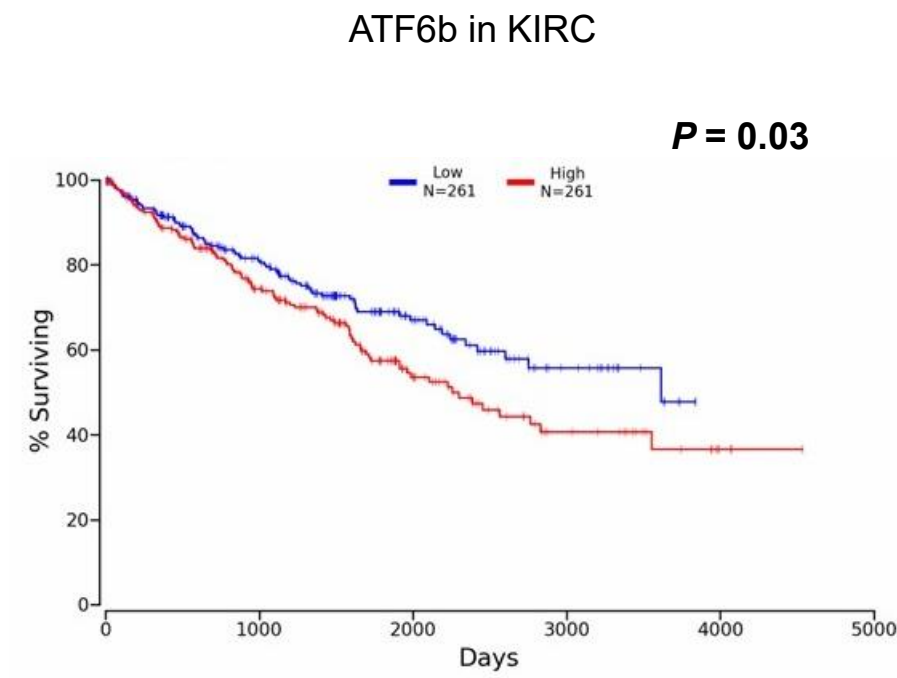

Supplementary Figure S2

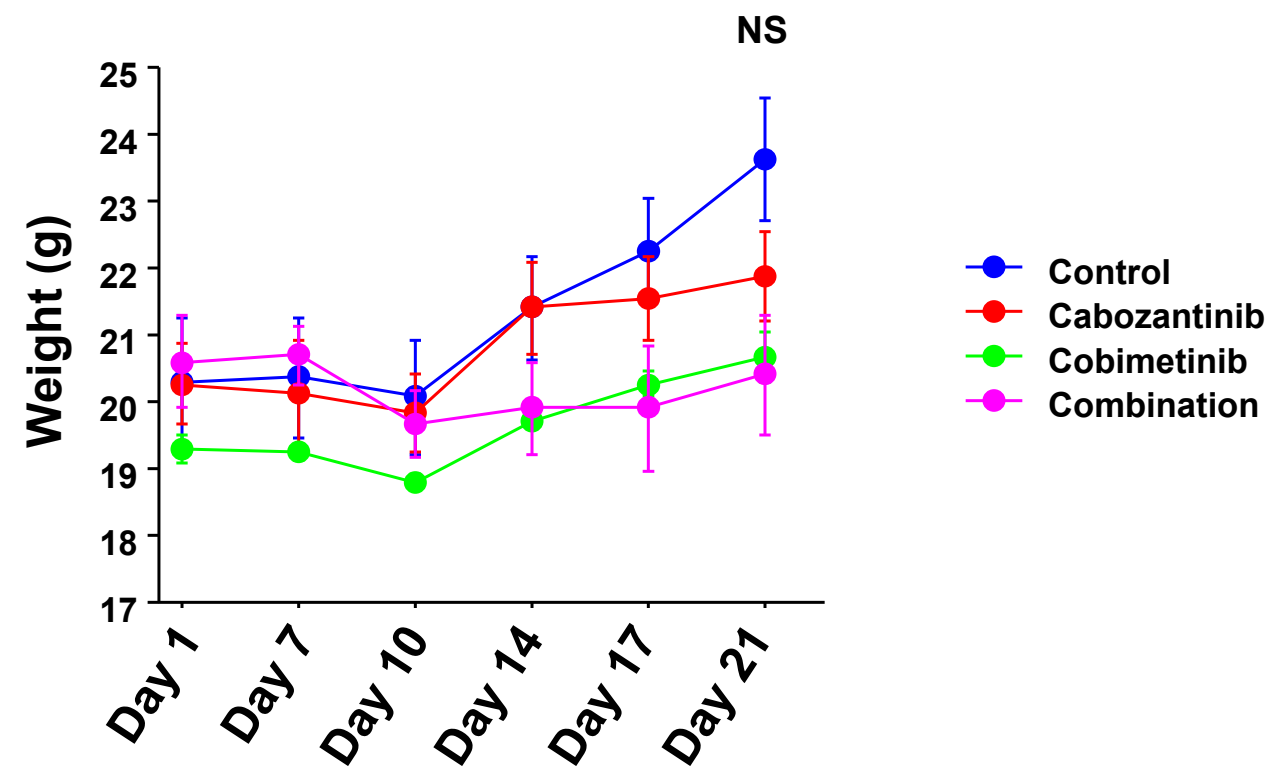

Supplement: Supplementary file 1 [file genes-17-00789-s001.zip › genes-4412660 Supplementary Figures S1 and S2.pdf]

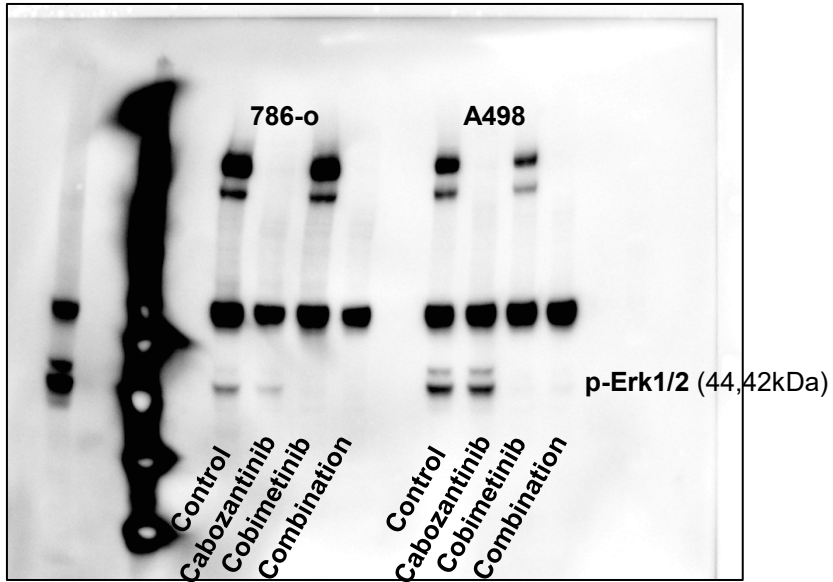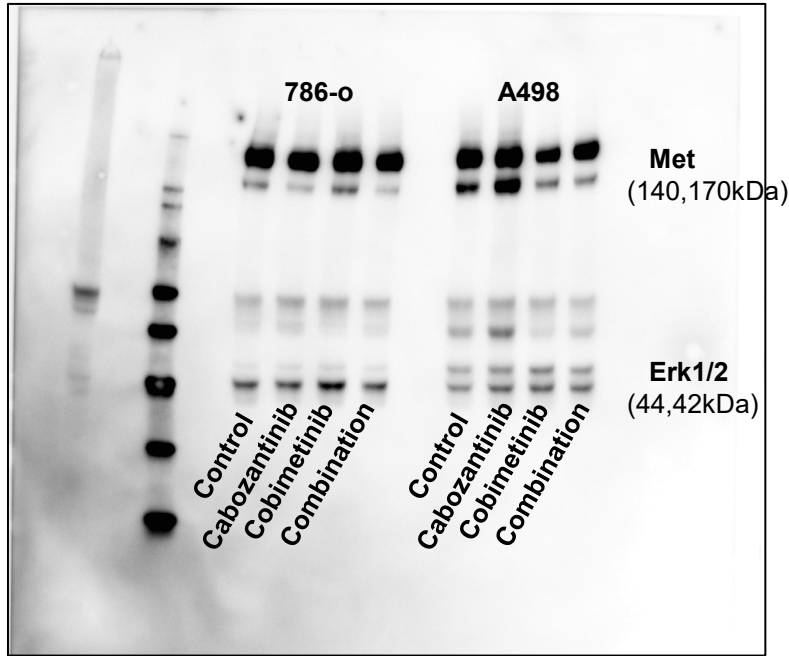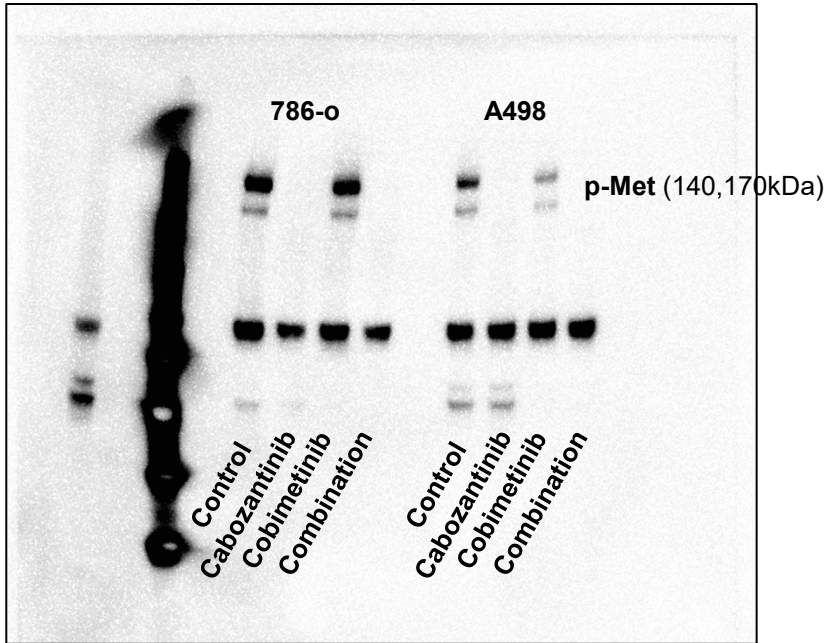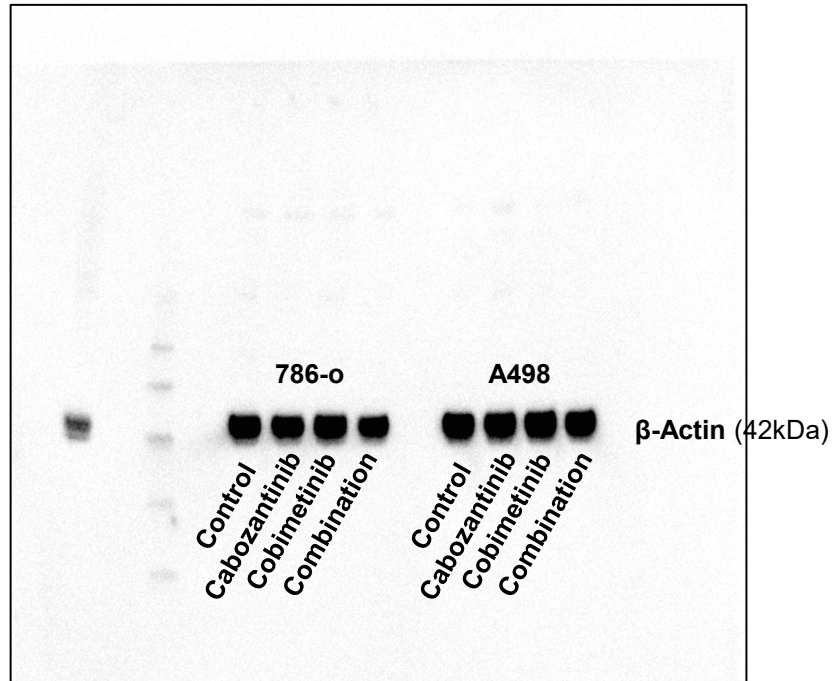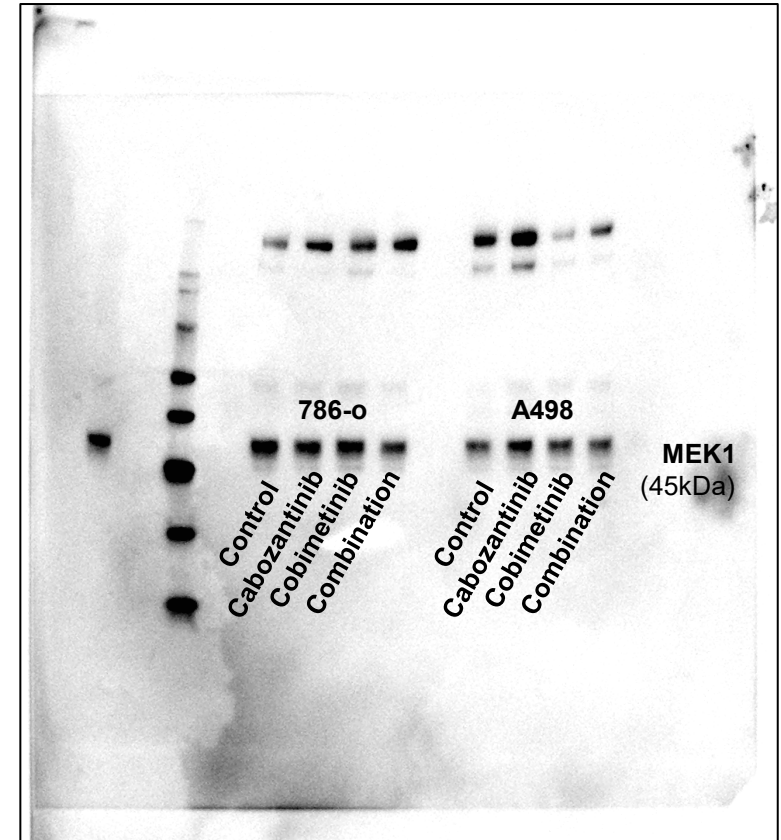

Supplement: Supplementary file 1 [file genes-17-00789-s001.zip › genes-4412660 Supplementary Figure S3. Uncropped WB.pdf]
